# Quantifying the effects of exceptional fossil preservation on the global availability of phylogenetic data in deep time

**DOI:** 10.1371/journal.pone.0297637

**Published:** 2024-02-14

**Authors:** C. Henrik Woolley, David J. Bottjer, Frank A. Corsetti, Nathan D. Smith

**Affiliations:** 1 Dinosaur Institute, Natural History Museum of Los Angeles County, Los Angeles, California, United States of America; 2 Department of Earth Sciences, University of Southern California, Los Angeles, Los Angeles, California, United States of America; Dakota State University, UNITED STATES

## Abstract

Fossil deposits with exceptional preservation (“lagerstätten”) provide important details not typically preserved in the fossil record, such that they hold an outsized influence on our understanding of biodiversity and evolution. In particular, the potential bias imparted by this so-called “lagerstätten effect” remains a critical, but underexplored aspect of reconstructing evolutionary relationships. Here, we quantify the amount of phylogenetic information available in the global fossil records of 1,327 species of non-avian theropod dinosaurs, Mesozoic birds, and fossil squamates (e.g., lizards, snakes, mosasaurs), and then compare the influence of lagerstätten deposits on phylogenetic information content and taxon selection in phylogenetic analyses to other fossil-bearing deposits. We find that groups that preserve a high amount of phylogenetic information in their global fossil record (e.g., non-avian theropods) are less vulnerable to a “lagerstätten effect” that leads to disproportionate representation of fossil taxa from one geologic unit in an evolutionary tree. Additionally, for each taxonomic group, we find comparable amounts of phylogenetic information in lagerstätten deposits, even though corresponding morphological character datasets vary greatly. Finally, we unexpectedly find that ancient sand dune deposits of the Late Cretaceous Gobi Desert of Mongolia and China exert an anomalously large influence on the phylogenetic information available in the squamate fossil record, suggesting a “lagerstätten effect” can be present in units not traditionally considered lagerstätten. These results offer a phylogenetics-based lens through which to examine the effects of exceptional fossil preservation on biological patterns through time and space, and invites further quantification of evolutionary information in the rock record.

## Introduction

Any effort to reconstruct biodiversity in Earth’s geologic past hinges on the ability to characterize and quantify the downstream effects of bias in the fossil record. Fossil data, from a cellular to a global level, is subject to various geological filters [1, 2], taphonomic filters [3], and sampling filters [4, 5], that distort our understanding of ancient life. If left unaccounted for, these biases represent significant barriers to paleobiological inquiry [6] that can obscure the myriad of anatomical, ecological, and evolutionary signals contained in the rock record.

Although the incompleteness of fossil data is a long-established reality in paleobiology [5–8], a growing number of studies have employed a variety of novel metrics to characterize and quantify fossil record biases in organismal groups [9–24]. Combined with an increasing number of sampling proxies to account for a multitude of biases in paleobiological data [22, 25–27], we are equipped with an advanced toolkit to explore outstanding questions related to biodiversity in the fossil record. However, the association between fossil record incompleteness and our ability to infer evolutionary relationships of fossil organisms remains a pressing, but underexplored question [3, 28–31].

Using established “completeness metrics” for a fossil record is one of the most straightforward ways to assess potential barriers to reconstructing the phylogeny of extinct organisms. In this study, we use the Character Completeness Metric (CCM [10]), to measure the percentage of phylogenetic characters that can be scored for a given fossil species, based on the preserved elements of a species’ anatomy. The CCM is extremely useful in quantifying both the physical completeness of the fossil record of organismal groups, but also quantifies potential barriers to accurately reconstructing their evolutionary relationships, which are essential to the frameworks that synthetic studies of biodiversity [32], biostratigraphy [33], and paleobiogeography [34] rely upon. Here, we use the CCM to quantify the amount of phylogenetic information available in the global fossil records of three prominent tetrapod groups: non-avian theropod dinosaurs (defined here as all theropods excluding Avialae [20]), Mesozoic birds (defined here as all Mesozoic taxa included in Avialae [11]), and squamates (e.g., lizards, snakes, amphisbaenians, and mosasaurs). The biases we set out to quantify in detail are **1)** the relationship between regional sampling intensity and amount of phylogenetic information preserved; **2)** the relationship between depositional environment and amount of phylogenetic information preserved.

The approach in this study allows us to quantify which depositional settings are more likely to preserve higher amounts of phylogenetic information, and which landmasses have historically produced the highest amount of phylogenetic information. In examining these two biases, we can also quantitatively visualize novel patterns in the preservation of phylogenetic data in the fossil record. Specifically, we highlight an unexpected finding: the lizard assemblage from the Upper Cretaceous aeolian deposits of the Gobi Desert (Djadokhta and Baruungoyot Formations [35–40]) preserves an extremely high amount of phylogenetic information compared to all other geologic units in the squamate fossil record, including diverse squamate assemblages found in established “lagerstätten” deposits (e.g., Solnhofen [41–44], Jehol [45–47], Messel, [48–53], Quercy Phosphorites [51, 54–57]).

“Lagerstätten” [58–60] are unusually rare confluences of deposition, taphonomy, and diagenesis that produce exceptionally preserved fossil assemblages. Lagerstätten are found across billions of years of geologic time [61] and include some of the most famous and intensely-studied fossil assemblages on the planet [62–66]. The biological information we gain from rare lagerstätten deposits often substantially exceeds what is preserved in the vast majority of “normal” fossil deposits, and as such have been identified as having an anomalous impact on studies of ecology, diversification and extinction through time [4, 6, 59, 61, 67]. Commonly referred to as the “lagerstätten effect”, the outsized influence that rare exceptionally preserved deposits have on the understanding of evolution through time can be at once a paleobiological boon to researchers [61] and a major source of bias in our understanding of the morphological evolution of a given group or groups of organisms [4, 6, 67]. The “lagerstätten effect” is famously acute in reconstructing global biodiversity changes through geologic time [6, 61, 67]. Lagerstätten often preserve such anomalously high amounts of fossil species relative to typical “baseline” sedimentary processes that estimations of global fossil diversity and abundance are more accurate when removing them from the dataset [61, 67]. This paradoxical bias has other important downstream impacts in analyzing biological and evolutionary patterns in Earth’s past. In particular, when it comes to inferring the phylogenetic relationships of organismal groups using their fossil record, the “lagerstätten effect” on phylogenetic information content and incorporation of taxa into analyses remains underexplored, and needs to be characterized to avoid bias in evolutionary interpretations [3–4, 6, 28–29, 67].

The pattern this study describes in the squamate fossil record invites us to consider whether deposits that lack traditional lagerstätten features (soft-bodied preservation, extreme abundance, etc.), such as the Djadokhta and Baruungoyot Formations in the Late Cretaceous Gobi Desert [35–40], can still have lagerstätten-level preservation of evolutionary information. These units could thus impart a phylogenetic “lagerstätten effect” [67] that overloads phylogenetic analyses with taxa from one geographic area or timeframe, potentially biasing estimates of important aspects of evolutionary history, such as biogeography [34] and lineage divergence times [6]. We find that the effect that the Djadokhta and Barrungoyot Formations have over the quality of the fossil record of squamates is similar to the well-established “lagerstätten effect” due to the exceptionally high amount of phylogenetic information in Mesozoic birds from the Jehol Biota on our understanding of avialan evolution [11, 68]. Non-avian theropods are well-represented in both the Late Cretaceous Gobi and Jehol Biota, but have a much more complete global fossil record than either squamates or Mesozoic birds, and thus are not as vulnerable to a “lagerstätten effect” on their phylogenetic history. These results offer a novel lens through which to examine the effects of exceptional preservation on phylogenetic information content in fossil groups.

## Materials & methods

### Occurrence datasets

Taxonomic, stratigraphic, and occurrence-based data from the published squamate fossil record were downloaded from the Paleobiology Database (PBDB). We limited our data to the species level, as both phylogenetic datasets used in this study (**Gauthier et al., 2012** [GEA] [69]; **Simões et al., 2018** [SEA] [70]) use species as their operational taxonomic units. The dataset includes 797 extinct species and 16,983 specimens from 469 localities that range from the Middle Triassic (Anisian) to the late Pleistocene in age. We specifically filtered out any fossil squamate material assigned to extant species, even though there are Quaternary fossils assigned to extant taxa. We mainly did this because, if the taxonomic assignments are correct, we presumably would still have 100% of the morphological data to score because the taxon is extant and thus full skeletons (and soft tissues in wet specimens) would be available. PBDB information for each species was vetted using 492 published specimen and locality descriptions (See **S1 and S3 Files**). Each occurrence entry from the PBDB was vetted using an exhaustive search and survey of all publications associated with the taxon, and, in some cases, where no geologic setting is included in the description, the geologic unit that preserves the taxa. This allowed for removal of many of out-of-date taxonomic assignments, general stratigraphic positions, duplicates, etc. present in the PBDB dataset. Additionally, for comparisons in the Gobi Desert assemblage, we incorporated previously published skeletal and character completeness assessments for 402 species of non-avian theropods [20] and 128 species of Mesozoic birds [11]. No permits were required to carry out this study; the dataset was compiled from publicly-available electronic collections databases and peer-reviewed publications (See **S1 and S3 Files**).

### Quantification & statistical analyses

**Fig 1:**
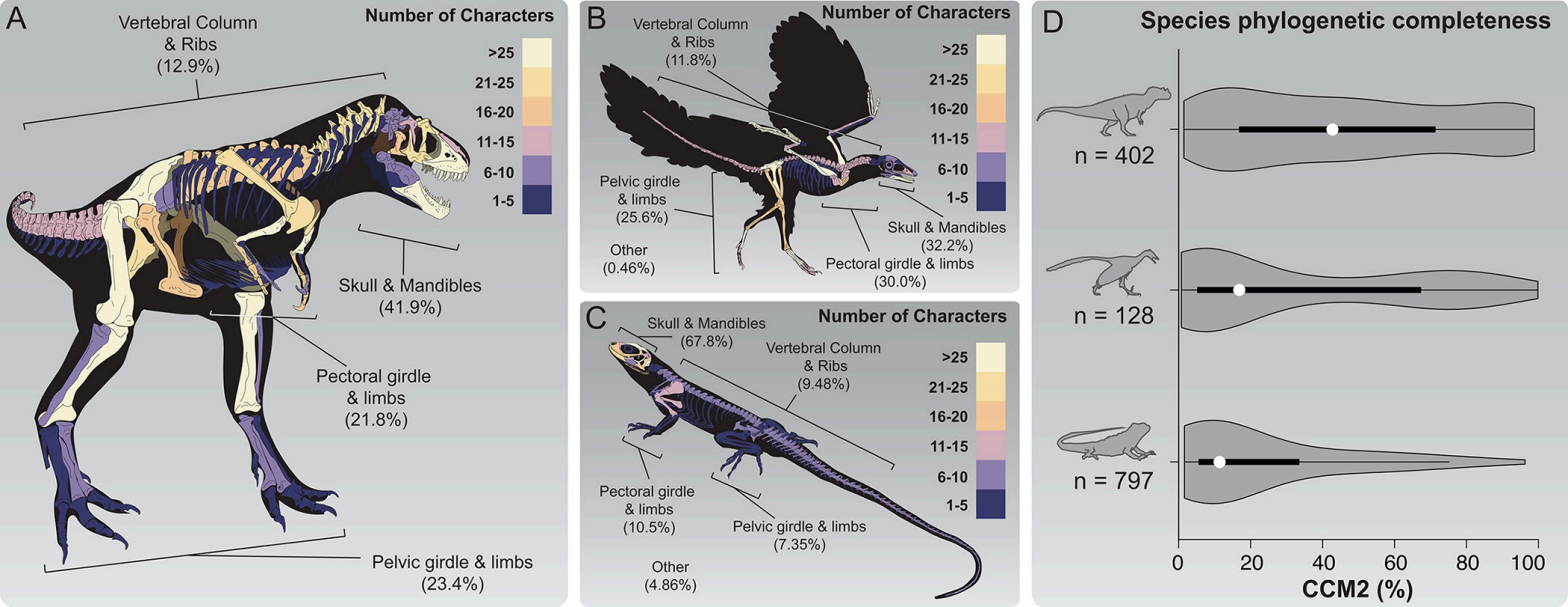
Visualization of the Character Completeness Metric (CCM2) in the fossil record of non-avian theropod dinosaurs, Mesozoic birds, and squamates. **A)** Heat map of non-avian theropod phylogenetic character density across the skeleton of an example theropod, *Teratophoneus curriei*, [71] on public display at the Natural History Museum of Utah. **B)** Heat map of avian phylogenetic character density across the skeleton of an example Mesozoic bird, *Archaeopteryx lithographica* [72], on public display at the Natural History Museum of Los Angeles County. **C)** Heat map of character density across an example squamate skeleton (*Uta stansburiana*). **D)** Summary violin plots for the phylogenetic completeness (CCM2) of described species in the global fossil record of non-avian theropods (top), Mesozoic birds (middle) and squamates (bottom). *White dot*: median; *black bar*: interquartile range; *black line*: 95% confidence interval. Silhouettes traced from publicly-available renderings at www.phylopic.org.

To assess the completeness of the global fossil record of squamates, non-avian theropods, and Mesozoic birds, we used the Character Completeness Metric 2 (CCM2 [10]), which measures the percentage of phylogenetic characters that can be scored for all specimens referred to a fossil species (**Fig 1**). We used two datasets that represent competing morphology-based hypotheses of squamate evolutionary relationships (GEA and SEA). We combined these two datasets and removed overlapping characters for a total of 860 scorable phylogenetic characters. Individual species’ completeness was scored based on the presence of individual skeletal elements for which a corresponding portion of the combined phylogenetic characters could be scored. We carried out scoring species’ CCM2 percentage by using only the characters that the species could be scored for. For instance, many fossil snake and amphisbaenian species do not possess limbs (i.e., no matter how complete the skeletal remains are, these species would always be missing 150 characters of the limbs and limb girdles). Because of this disparity, we measured legless squamate taxa’s “True Completeness” out of 710 characters instead of the 860 we used for squamates with four limbs.

For the non-avian theropod dataset [20], we converted previously-assessed Skeletal Completeness Metric 2 (SCM2) values to CCM2 values using a character matrix assessing higher-level relationships of theropod dinosaurs [73] and a recent iteration of the Theropod Working Group (TWiG) character matrix assessing the relationships of coelurosaurs [74]. We incorporated the Mesozoic bird CCM2 dataset assessed by Brocklehurst et al. (2012) [11] as-is. All data visualization and statistical tests were carried out in R [75]. Violin plots of various partitions of non-temporal range data were visualized using the vioplot package in R [76]. Nonparametric pairwise statistical comparisons of non-temporal range CCM2 data were carried out using the Mann-Whitney U-Test and the Kolmogorov-Smirnov Test (**S2 File**). Because we performed multiple statistical comparisons among both landmasses and among depositional environments, statistical tests were run using a Bonferroni correction on the α value.

## Results

### General phylogenetic completeness of non-avian theropods, birds, and squamates

In our quantitative approach to assessing the availability of phylogenetic information in the fossil record, we use the Character Completeness Metric 2 (CCM2 [10]; see **Materials & Methods**), which measures the percentage of phylogenetic characters that can be scored for all specimens assigned to a fossil species. The phylogenetic datasets corresponding to each group in this study (**Fig 1**) contain hundreds of characters (non-avian theropods: 774 [74]; Mesozoic birds: 653 [11]; squamates: 860 this study) that reflect the variation in skeletal anatomy found therein. Non-avian theropods (**Fig 1A**) and Mesozoic birds (**Fig 1B**) have concentrations of phylogenetic characters that are more or less evenly distributed throughout the skeleton, whereas squamates (**Fig 1C**) have over two-thirds of their phylogenetic characters concentrated in the skull and mandibles. This can probably be explained by the large amount of disparity in the skulls of squamates and/or the presence of numerous legless lineages of squamates (e.g., snakes, amphisbaenians, dibamids), necessitating an emphasis on shared characters in the skull to assess higher level evolutionary relationships ([69, 70]).

The generalized distributions of preserved phylogenetic information in non-avian theropods, Mesozoic birds, and squamates across time and space reveal some noteworthy patterns (**Fig 1D**). Our squamate dataset contains the most species (n = 797) and spans the most time (Middle Triassic to Late Pleistocene), yet on the whole preserves the least amount of phylogenetic information. Mesozoic birds contain the least amount of species (n = 128) and shortest span of geologic time (Late Jurassic–Late Cretaceous), have a median CCM2 value that is not statistically significantly different from that of squamates (α = 0.05, p = 0.07535, **S2 File**), but have a relatively higher proportion of species that preserve a high amount of phylogenetic information. Non-avian theropods, as one of the most intensely-studied groups of fossil animals [20, 77, 78] are both well-sampled (n = 402) and preserve a significantly higher median CCM2 value and distribution of CCM2 scores than Mesozoic birds and squamates (all p-values<<<< 0.05, **S2 File**).

### Preservation of phylogenetic information in the Early Cretaceous Jehol Biota

**Fig 2:**
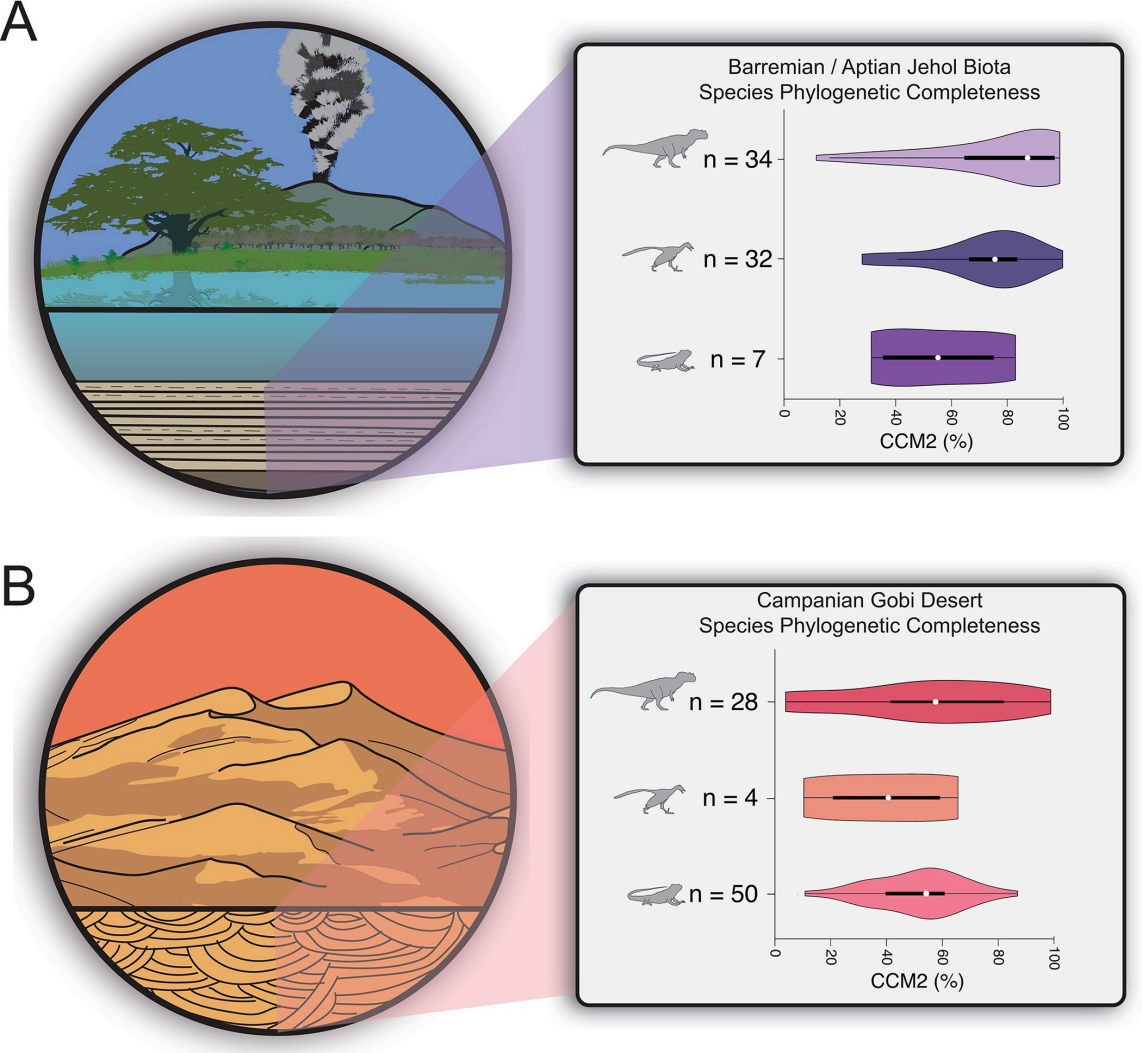
Comparisons of species phylogenetic completeness of non-avian theropod dinosaurs, birds, and squamates preserved in the lacustrine konservat-lagerstätten deposits of the Yixian and Jiufotang formations (Jehol Biota) and the aeolian lagerstätten deposits of the Djadokhta and Baruungoyot formations (Campanian Gobi Desert). **A)** *Left*: Paleoenvironmental reconstruction of the Yixian and Jiufotang Formations. *Right*: violin plots comparing distributions of species completeness (CCM2) values among non-avian theropods, birds and squamates represented in the Barremian/Aptian Jehol Biota assemblage. **B)** *Left*: Paleonvironmental reconstruction of the Djadokhta and Baruungoyot Formations. *Right*: Violin plots comparing distributions of species completeness (CCM2) values among non-avian theropods, birds and squamates represented in the Late Cretaceous (Campanian) Djadokhta and Baruungoyot Formation assemblages. *White dot*: median; *black bar*: interquartile range; *black line*: 95% confidence interval. Silhouettes traced from publicly-available renderings at www.phylopic.org.

Non-avian theropods and birds are famously well-preserved and well-represented in the Jehol Biota assemblages in the Barremian-Aptian Yixian and Jiufotang formations in the Liaoning and Inner Mongolia provinces of China (**Fig 2A**; [68, 79]). Squamates, while not as abundant as theropods (including birds), still preserve remarkably complete specimens (**Fig 2A**; [45, 46]) that reveal intriguing aspects of squamate anatomical evolution [46] and biotic interactions [47]. Side-by-side statistical comparisons of CCM2 distributions (**Fig 2A and S2 File**) of all three groups reflect a similar pattern to that observed in comparisons of their global distributions through time (**Fig 1D**). Non-avian theropods preserve a significantly higher median CCM2 value (87.28%) and distributions of CCM2 values than those observed in both birds and squamates (all p-values < 0.05, **S2 File**), and while birds preserve a higher median CCM2 value (75.57%) than squamates (55.17%) and more phylogenetic information overall, there is not a statistically significant difference between their distributions (α = 0.05; Mann-Whitney U: p = 0.09584; Kolmogorov-Smirnov: p = 0.17650, **S2 File**).

### Preservation of phylogenetic information in the Late Cretaceous (Campanian) Gobi Desert

The aeolian deposits of the late Campanian Djadokhta and Baruungoyot formations in Mongolia and China preserve one of the most iconic assemblages of theropod dinosaurs in the fossil record, including *Velociraptor* and myriad oviraptorosaurs and ornithomimosaurs [80–82]. Additionally, the Campanian Gobi Desert preserves historically important bird fossils [83–85], even though they are not as abundant as other theropods. Where the truly remarkable preservation lies is within the record of squamates, with at least 50 described species of three-dimensionally-preserved skulls and partial skeletons [35–37, 40]. Side-by-side comparisons of the distributions of CCM2 values (**Fig 2B**) among all three tetrapod groups show comparable, high levels of preservation compared to the overall fossil records (**Fig 1D**), similar to the patterns observed in the Jehol Biota (**Fig 2A**). Unlike the Jehol Biota, however, there are no statistically significant differences in the median CCM2 percentages and distribution shapes in all three surveyed Campanian Gobi groups (all p- values > 0.05, **S2 File**). In the Campanian Gobi, the non-avian theropod median CCM2 (59.91%) and the bird median CCM2 (28.25%) are significantly lower than in the Jehol, whereas the squamate median CCM2 in the Gobi (53.67%) is comparable to that seen in the Jehol (55.17%).

### Continental-scale influence of exceptional preservation on phylogenetic character data

**Fig 3:**
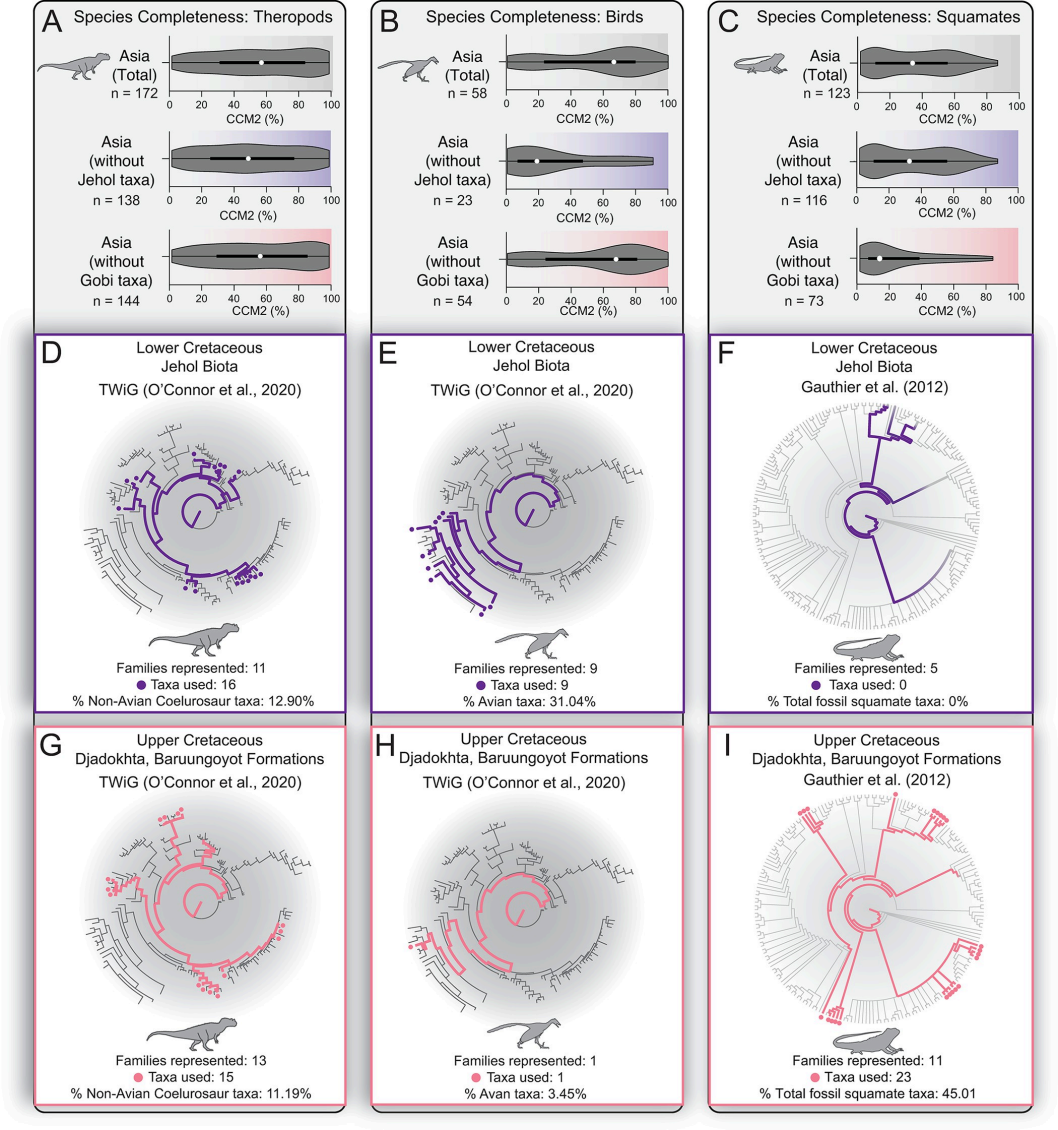
Comparisons of continental-scale differences in the effects of lagerstätten deposits (Djadokhta, Baruungoyot formations; Jehol Biota) on the preservation of phylogenetic character data. **A)** Distribution of all non-avian theropod CCM2 values in Asia (top), and distribution of CCM2 values when removing taxa from the Lower Cretaceous Jehol Biota (middle, violet background) and Campanian Gobi Desert (bottom, coral background). **B)** Distribution of all Mesozoic bird CCM2 values in Asia (top), and distribution of CCM2 values when removing taxa from the Lower Cretaceous Jehol Biota (middle, violet background) and Campanian Gobi Desert (bottom, coral background). **C)** Distribution of all fossil squamate CCM2 values in Asia (top), and distribution of CCM2 values when removing taxa from the Lower Cretaceous Jehol Biota (middle, violet background) and Campanian Gobi Desert (bottom, coral background). *White dot*: median; *black bar*: interquartile range; *black line*: 95% confidence interval. **D)** Summary of the number of non-avian theropod families represented from the Jehol Biota assemblage on the Theropod Working Group (TWiG) [74] phylogeny. **E)** Summary of the number of avian families represented from the Jehol Biota assemblage on the Theropod Working Group (TWiG) [74] phylogeny. **F)** Summary of the number of squamate families represented from the Jehol Biota assemblage on the GEA [69] phylogeny. **G)** Summary of the number of non-avian theropod families represented from the Djadokhta/Baruungoyot assemblage on the Theropod Working Group (TWiG) [74] phylogeny. **H)** Summary of the number of avian families represented from the Djadokhta/Baruungoyot assemblage on the Theropod Working Group (TWiG) [74] phylogeny. **I)** Summary of the number of squamate families represented from the Djadokhta/Baruungoyot assemblage on the GEA [69] phylogeny. Silhouettes traced from publicly-available renderings at www.phylopic.org.

To examine the effects of lagerstätten-style fossil preservation on phylogenetic information content in the fossil records of continents, we performed several pairwise statistical comparisons between median CCM2 percentages (Mann-Whitney U) and cumulative distribution (Kolmogorov-Smirnov) in the fossil records of non-avian theropods, birds and squamates in Asia with and without the Jehol and Gobi assemblages (**Fig 3A–3C**). We recovered different patterns of the influence of the Jehol Biota and Djadokhta/Baruungoyot formations on CCM2 distributions of representative groups. For non-avian theropod dinosaurs, removing taxa found in the Jehol Biota does not significantly affect the median CCM2 value and distribution shape (**Fig 3A and S2 File**). Similarly, removing Gobi desert taxa has no significant effect on the distribution of CCM2 values of taxa found in Asia (**Fig 3A and S2 File**). In fact, removing all non-avian theropod taxa found in the Jehol Biota and Campanian Gobi Desert does not significantly affect the median CCM2 value or distribution shape of species CCM2 values in Asia (**S2 File**). For Mesozoic birds, removing taxa found in the Jehol Biota from the total dataset from Asia yields a median CCM2 value and a distribution of CCM2 values that are significantly lower (**Fig 3B**). Conversely, removing Gobi desert taxa (n = 4) unsurprisingly has no significant effect on the distribution of CCM2 values of taxa found in Asia (**Fig 3B and S2 File**). The patterns observed in the continental record of Asia for squamates is the inverse of that seen in birds (**Fig 3C**). We find that by removing the squamate taxa found in the Jehol Biota (n = 7), we unsurprisingly recover a distribution of CCM2 values that are not statistically significantly different from the total dataset. If we remove the taxa found in the Gobi Desert assemblage, the median CCM2 value becomes significantly lower, and the distribution shape of CCM2 values also decreases significantly (**S2 File**).

### Representation of lagerstätten taxa in phylogenetic analyses

The patterns in CCM2 distributions and taxonomic abundance in non-avian theropods, birds and squamates found in the Jehol Biota and the Campanian Gobi Desert may play a role in how these taxa are incorporated into phylogenetic analyses (**Fig 3D–3I**). In non-avian theropods (**Fig 3D and 3G**), the taxa from Jehol Biota and Djadokhta/Baruungoyot incorporated into the Theropod Working Group (TWiG) dataset [74] are roughly equal in terms of families/lineages represented (Gobi Desert: 13; Jehol: 11) and the percentage of total taxa used in the analysis (Gobi Desert: 11.19%; Jehol: 12.90%). For the avian dataset (**Fig 3E and 3H**), 9 families and 31.04% of avian taxa used in the TWiG dataset are derived from the Jehol Biota, while only one taxon from the Djadokhta/Baruungoyot formations is incorporated into the TWiG dataset. For the squamate phylogenetic analyses (GEA [69] and SEA [70], **Fig 3F and 3I,**), no taxa from the Jehol are incorporated into the GEA analysis, while only 1 taxon, *Dalinghosaurus longidigitus*, is incorporated in the SEA analysis. Conversely, the assemblage in the Djadokhta/Baruungoyot formations contributes the most families/lineages represented (GEA: 11; SEA: 7) and the highest percentage of fossil taxa incorporated into the analysis (GEA: 45.01%; SEA: 40.91%) compared to any other assemblage in the squamate fossil record. These results illustrate, especially in the case of Mesozoic birds and squamates, that a single lagerstätte deposit can produce a disproportionate amount of fossil taxa incorporated into phylogenetic analyses relative to the global sample.

### The phylogenetic “lagerstätten effect” in a global sampling context

**Fig 4:**
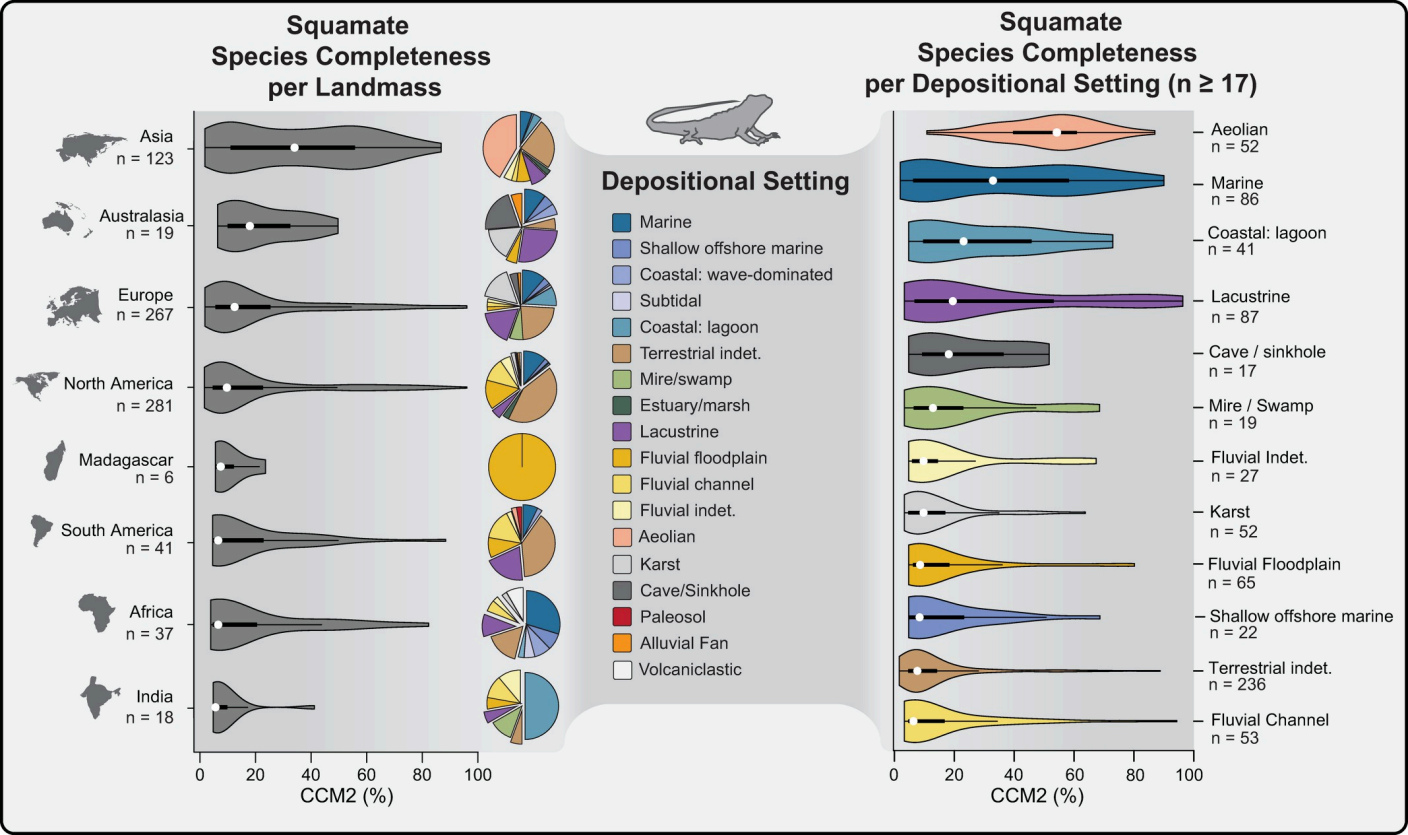
Summary of the effects of regional sampling and depositional setting on the amount of available phylogenetic information in the squamate fossil record. **Left:** violin plots of the distribution of squamate Character Completeness Metric 2 (CCM2) percentages per landmass. Not displayed: Antarctica (n = 2); Caribbean (n = 3). **Pie charts** show the relative proportions of depositional environments in which fossil squamate species are found per corresponding landmass. **Right:** violin plots of the distribution of squamate CCM2 percentages per depositional setting (right). *White dot*: median; *black bar*: interquartile range; *black line*: 95% confidence interval.

Out of landmasses that have sample sizes ≥ 6 species of non-avian theropods, Mesozoic birds, and squamates, respectively, the record from Asia contains the highest median CCM2 percentage (**Fig 4 and S1–S3 Files**). We ran an additional set of pairwise statistical tests to assess the significance of these observed differences. In the global record of phylogenetic information preserved in non-avian theropods (**Fig 1**), we find that the median CCM2 and distribution shape from Asia is significantly different from that of Africa, Europe, and South America. We also found no significant differences in the distributions found in the well-sampled theropod-bearing units of North America, as well as Australasia, India, and Madagascar, although the lack of difference between the latter three may be due to their small sample size (**S2 File**). Thus, the signal from preserved phylogenetic information in non-avian theropod assemblages in the Jehol Biota and Campanian Gobi Desert is not only lost in the well-sampled and highly complete record from Asia (see above), but is also lost in a global context due to the well-sampled, highly complete record from North America, which contributes to a fossil record that preserves a significantly higher amount of phylogenetic information relative to birds and squamates (**Fig 1D**). These results suggest that the more complete global sample of non-avian theropods is less vulnerable to a “lagerstätten effect” on phylogenetic information content. Further interrogation of this pattern is needed and warranted, but is beyond the scope of the current study.

When we remove bird taxa found in the Jehol Biota from the Asia dataset (**Fig 2B**), we recover a distribution of CCM2 values that are significantly lower, such that the median CCM2 value and distribution shape are statistically indistinguishable from the CCM2 distributions of most landmasses, except for the highly incomplete record in North America (Mann-Whitney U: p = .0081; Kolmogorov-Smirnov: p = 0.0034). This illustrates the outsized impact of this unique Jehol avian assemblage on the preservation of phylogenetic character data in Asia, which in turn strongly influences the distribution of phylogenetic information in the global fossil record of Mesozoic birds. The outsized, “lagerstätten effect” that the Jehol Biota has on the completeness of our understanding of Mesozoic bird evolution has been examined in detail in numerous studies [11, 68, 79], as has the effects of multitudes of hypoxic lacustrine lagerstätten deposits on our understanding of fossil terrestrial ecosystems [68, 86–88]. The remainder of the results section will focus on the novel findings from the squamate fossil record and the aeolian deposits of the Campanian Gobi Desert.

The left panel of **Fig 4** displays the distribution of CCM2 percentages for eight landmasses in order of increasing median value of fossil squamate species. Pairwise statistical comparisons between median CCM2 percentages (Mann-Whitney U) and cumulative distribution (Kolmogorov-Smirnov) among landmasses show no statistically significant differences, with only one notable exception in the data obtained from Asia (**Fig 4 and S2 File**). For all fossil squamate species found in Asia, the median CCM2 percentage and the cumulative distribution shape of CCM2 percentages are statistically significantly different from all other landmasses, except for Madagascar and Australasia (0.05 > p > α). The global distribution of phylogenetic information in the squamate fossil record is therefore similar to that of non-avian theropods and Mesozoic birds, with Asia preserving the highest amount of information. The key difference for squamates lies in the dominant depositional setting in which the highly complete fossils are found: aeolian dune deposits.

### Depositional setting and fossil squamate completeness

Pie charts illustrating the relative abundance of fossil squamate species found in different depositional environments are shown on the right-hand side of the violin plots of the CCM2 distributions for landmasses in **Fig 4**. The distribution of CCM2 values per depositional environment with 17 or more sampled fossil squamate species are shown on the right-hand panel of **Fig 4**. Pairwise statistical comparisons between median CCM2 percentages (Mann-Whitney U) and cumulative distribution (Kolmogorov-Smirnov) among these distributions reveal several patterns (**S2 and S3 Files**), but most importantly, the median and distribution shape of CCM2 percentages of fossil squamate species found in aeolian environments is statistically significantly different from every other surveyed environment apart from the median marine CCM2 value. The cause for this discrepancy is almost certainly due to the presence of 50 highly complete lizard species sampled from the Upper Campanian Djadokhta and Baruungoyot aeolian deposits in Mongolia and China. Indeed, if we remove these taxa from the Asia sample, the distribution shape and median value do not showcase any statistically significant differences from the rest of the sampled landmasses (all p-values > α, except for the comparison of median CCM2 of Asia and India, p = 0.00013, **S2 File**). The squamates found in the Late Cretaceous Gobi Desert demonstrate the profound effects that cases of exceptional preservation of phylogenetically-relevant characters (versus e.g., soft tissues or extreme abundance) have on fossil record completeness on continental and global scales.

### Gobi Desert squamates compared to other squamates preserved in lagerstätten deposits

**Fig 5:**
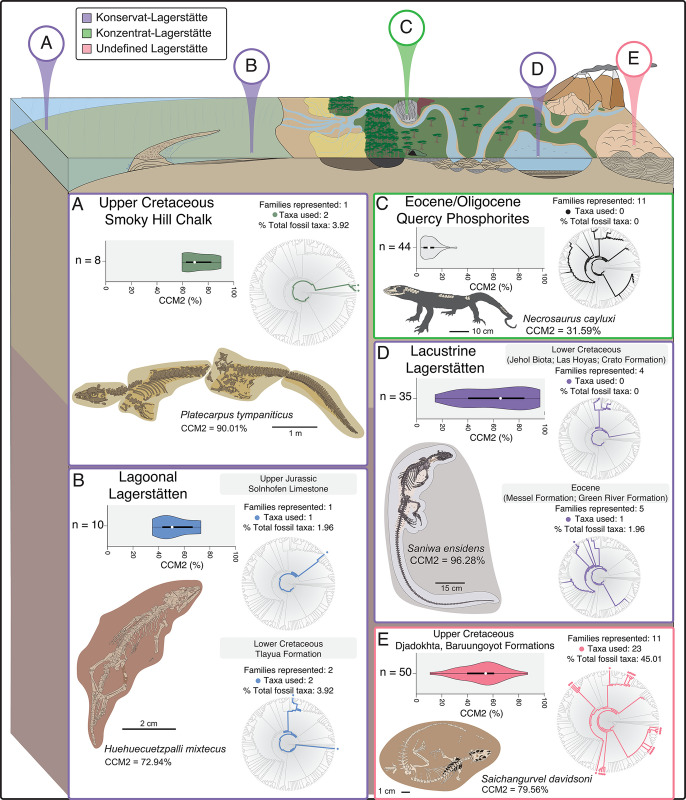
Summary of distributions of Character Completeness Metric 2 (CCM2) values for squamates found in established lagerstätten localities, with a summary of the number of families represented in localities using the Gauthier et al. (2012) [69] phylogeny. **A)** Distribution of CCM2 values and number of families of squamates found in the Upper Cretaceous Smoky Hill Chalk. Example species: *Platecarpus tympaniticus* [89]. **B)** Distribution of CCM2 values and number of families of squamates found in lagoonal lagerstätten deposits. Example species: *Huehuecuetzpalli mixtecus* [90]. **C)** Distribution of CCM2 values and number of families of squamates found in the Eocene/Oligocene Quercy Phosphorites. Example species: *Necrosaurus cayluxi* [51]. **D)** Distribution of CCM2 values and number of families of squamates found in lacustrine lagerstätten deposits. Example species: *Saniwa ensidens* [91]. **E)** Distribution of CCM2 values and number of families of squamates found in the Upper Cretaceous Djadokhta and Baruungoyot formations. Example species: *Saichangurvel davidsoni* [92].

We acknowledge that most depositional environments in **Fig 4** do not contain examples of exceptionally preserved squamate fossils. Therefore, we also compared the completeness of the Gobi Desert squamate assemblage to all other squamate assemblages found in deposits classified in some form or another as lagerstätten (**Fig 5 and S2 File**). Konservat-lagerstätten [58, 59] are characterized by the preservation of difficult to fossilize features (e.g., soft tissues) and often form under quick burial and anoxia/hypoxia associated with stagnation deposits, obrution deposits, and conservation traps such as peat or amber [60]. Konzentrat-lagerstätten [58], on the other hand, are characterized by the sheer number of fossilized specimens found in a single deposit, and form under a different set of sedimentological conditions that favor dense accumulation of fossilized remains (e.g., condensation deposits, placer deposits, and concentration traps [60]). While the Smoky Hill Chalk (**Fig 5A,** n = 8) and lagoonal lagerstätten deposits (**Fig 5B**, n = 10) do not have sufficient sample sizes of fossils for meaningful statistical comparison, we were able to compare the 50 Gobi desert squamates (**Fig 5E**) to the squamate assemblages from the Eocene/Oligocene Quercy Phosphorites in France (**Fig 5C**, Konzentrat-lagerstätte; n = 44) and squamates collectively found in Upper Cretaceous and Eocene lacustrine Konservat-lagerstätten deposits (**Fig 5D**, n = 35). The median CCM2 percentages, and distributions for these three lagerstätten types are statistically significantly different from one another (**S2 File**), with the Quercy Phosphorites representing a diverse but highly incomplete squamate assemblage, the Gobi Desert squamate assemblage being diverse and relatively complete, and the lacustrine lagerstätten representing highly complete but not as taxonomically diverse assemblages.

## Discussion

### The desert dunes of the Campanian Gobi Desert: A different kind of taphonomic anomaly?

We report significant differences in the preserved amount of phylogenetic information in the fossil record of non-avian theropods, Mesozoic birds, and squamates at the global level (**Fig 1D**) and in the Jehol lacustrine konservat-lagerstätte (**Fig 2A**). Conversely, and perhaps surprisingly, in the aeolian deposits of the Campanian Gobi Desert, we found a lack of statistically significant difference in the preservation of phylogenetic character data in squamates, non-avian theropods and birds (**Fig 2B**). Given the global context of the extreme statistical differences between the phylogenetic information preserved in the record of non-avian theropods and squamates (**Fig 1D and S1–S3 Files**), we interpret these results from the Djadokhta/Baruungoyot to be the product of uniform preservation and/or collection of phylogenetically-relevant information. This uniformity in phylogenetic completeness could be due to the unique preservation potential in aeolian sedimentary environments, such as immediate burial during large dune collapses and/or relatively quick burial during major sandstorms [38, 39]. On the other hand, the rich, 100-year history of intense sampling and research interest in the Djadokhta/Baruungoyot formations [81] could lead to the theropod, bird and squamate records reaching their maximized potential for preservation and completeness in an aeolian depositional setting.

Interestingly, the median CCM2 value and distribution of CCM2 percentages for Campanian Gobi Desert squamate species is extremely high when compared to the global distribution (p <<< 0.05, **Figs 1D, 2B and S2 File**), while there is a lack of statistical difference observed in the median CCM2 value and distribution of CCM2 percentages for Campanian Gobi Desert non-avian theropods and birds relative to their global distribution (**Figs 1D, 2B and S2 File**). This differs from the pattern seen for the Jehol (**Fig 2A and S2 File**), in which the CCM2 distributions of all three groups are significantly higher than the global distributions, demonstrating widespread elevated preservation of phylogenetic information.

Despite the differences between the Gobi/Jehol CCM2 distributions relative to the global records, it is noteworthy that the median CCM2 percentage and CCM2 distribution shape for squamates in the Jehol and Gobi Desert are not statistically significantly different from one another (**S2 File**). The small sample size of described species from Jehol might be to blame for this pattern, but even when comparing the Campanian Gobi Desert squamates to all described squamate species from lacustrine lagerstätten deposits (**Fig 5D, 5E and S2 File**), the differences in median CCM2 (Gobi: 53.67%; Lacustrine Lagersätten: 65.39%) value are not outlandishly different from one another, even if they are statistically significantly different (**S2 File**). Taken together, we interpret these results as evidence of lagerstätten-type exceptional preservation of phylogenetic information in at least the squamate fossil record from the aeolian deposits of the Djadokhta and Baruungoyot formations.

### The Gobi Desert “lagerstätten effect” in the squamate fossil record

The potential phylogenetic contributions of lagerstätten are as follows: 1) for konservat-lagerstätten, the general preservation of more complete specimens, including soft tissues, may provide additional characters not observable in non konservat-lagerstätte specimens. 2) for konzentrat-lagerstätten, the variation that results from the preservation of multiple specimens of a single species may allow for **a)** assessment of validity of phylogenetic characters and **b)** a higher probability of more complete specimens, in addition to **c)** the preservation of rare taxa.

The Gobi Desert “lagerstätten effect” on phylogenetic data in the squamate fossil record can be characterized via several patterns. **1)** Well-known konservat-lagerstätten deposits may have high preservation of squamate phylogenetic data via soft tissues, but the restricted geography and often limited temporal scope of these classic deposits likely contribute to less taxonomic abundance compared to the Djadokhta and Baruungoyot formations (**Fig 5A, 5B and 5D**); **2)** the Gobi Desert squamate assemblage is significantly more complete than Konzentrat-lagerstätten deposits like the Quercy Phosphorites, and therefore has distinctively different preservation of phylogenetic character data even if higher-level taxonomic diversity is similar **Fig 5C and 5E**); **3)** Although lacustrine lagerstätten possess the highest median CCM2 percentages and preserve fossils with the highest CCM2 percentages of the entire dataset, those deposits still contain lower overall alpha diversity of squamates, which results in fewer taxa available to use in phylogenetic analyses than in the Gobi Desert aeolian deposits (**Fig 5D and 5E**).

Thus, even when compared to squamate taxa preserved in established konservat- and konzentrat-lagerstätten deposits, the Djadokhta and Baruungoyot formations preserve squamate evolutionary information in a singular manner that differs from any other squamate bearing locality. As a result, the Djadokhta and Baruungoyot formations exert an anomalously large influence on the preservation and availability of squamate phylogenetic character data in Asia, which in turn influences our understanding of squamate evolutionary relationships through time and space, regardless of phylogenetic dataset (**Figs 3C, 3F, 3I and 5**). In the future, phylogenetic comparative analyses could test that if one removed fossil squamate taxa found in the Djadokhta and Baruungoyot formations from phylogenetic datasets, there might be a huge deficit in our understanding of divergence times of both fossil and extant squamate lineages. Even without such tests, the observations and analyses presented in this study show that the highly diverse an exceptionally complete squamate assemblage of the Djadokhta and Baruungoyot formations represents an extreme case of the “lagerstätten effect” on the completeness of the global fossil record of an organismal group.

### Distinguishing preservation signals from sampling intensity signals

**Fig 6:**
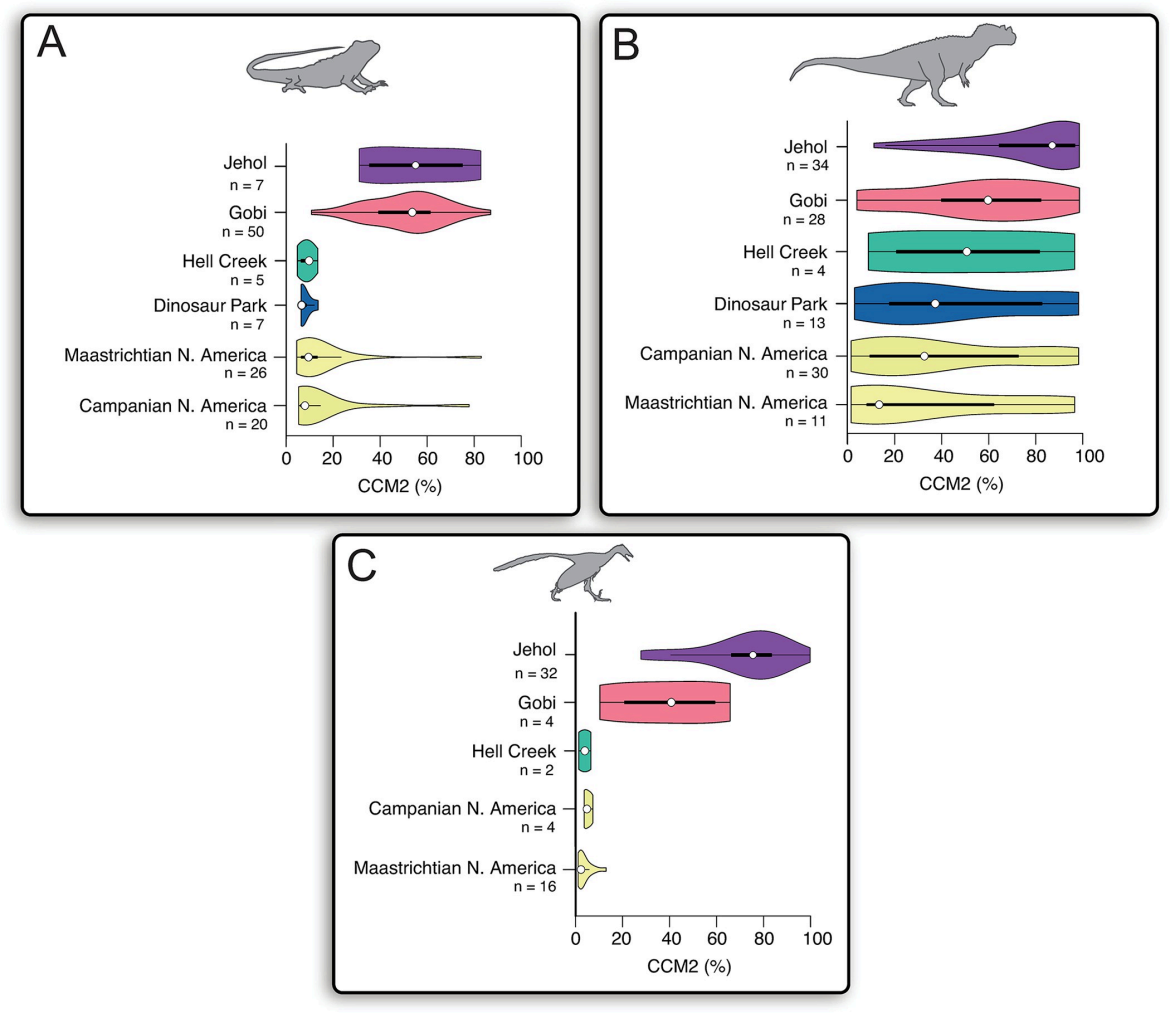
Comparison of CCM2 distributions of fossil squamate, non-avian theropod, and Mesozoic bird species from the Early Cretaceous Jehol/ Late Cretaceous Gobi to heavily-sampled Late Cretaceous units in North America (Maastrichtian Hell Creek Formation; Campanian Dinosaur Park Formation), as well as all paracontemporaneous taxa from heavily sampled Campanian and Maastrichtian terrestrial deposits of North America. **A)** Comparisons for squamates. **B)** Comparisons for non-avian theropod dinosaurs. **C)** Comparisons for Mesozoic birds. *White dot*: median; *black bar*: interquartile range; *black line*: 95% confidence interval.

To scrutinize whether the patterns we observe in the Late Cretaceous Gobi and Early Cretaceous Jehol are simply products of sampling intensity, we compared the distributions of CCM2 scores of non-avian theropods, Mesozoic birds, and squamates in these deposits to two intensely-sampled North American geological units that preserve our study groups: The Maastrichtian Hell Creek Formation [93, 94] and the Campanian Dinosaur Park Formation [95, 96] (**Fig 6**). While the sample size of each taxonomic group from these two geologic units (n ≤ 13) are too small for meaningful statistical comparison to the Jehol and Late Cretaceous Gobi, we observe that all median CCM2 percentages are substantially lower in the Hell Creek and Dinosaur Park formations than in either the Jehol or Late Cretaceous Gobi. Additionally, apart from Hell Creek non-avian theropods (n = 4, **Fig 6B**), all CCM2 distributions are skewed towards lower completeness percentages.

Because Upper Cretaceous terrestrial sedimentary sequences in North America comprise many of the most intensely sampled horizons on the planet [93, 97, 98], we also compared the Late Cretaceous Gobi and Jehol CCM2 distributions to the Campanian and Maastrichtian fossil records of our three study groups across North America (**Fig 6**). Nonparametric pairwise statistical comparisons (Mann-Whitney U-Test, Kolmogorov-Smirnov Test) show that Campanian and Maastrichtian North American squamate median CCM2 values are significantly lower than either the Jehol or Gobi medians and distribution shapes are significantly different than the Jehol or Gobi (all p-values < **α**, **Fig 6A and S2 File**). In non-avian theropods (**Fig 6B**), we find that the Jehol has a statistically-significantly higher median CCM2 and a statistically-significantly different distribution shape than the Campanian and Maastrichtian of North America. We found no statistically significant differences between the median CCM2 percentages and distribution shapes of non-avian theropods found in the Late Cretaceous Gobi Desert and those found in the Campanian and Maastrichtian of North America. However, the median CCM2 percentage for non-avian theropods found in the Gobi is higher, and does contain a greater proportion of highly complete non-avian theropods than in either the Campanian or Maastrichtian of North America (**Fig 6B**). For Mesozoic birds (**Fig 6C**), we found that Jehol and Gobi have statistically significantly higher median CCM2 percentages and statistically significantly different distribution shapes than the Maastrichtian of North America. Our sample sizes for bird fossils found in terrestrial sediments in the Campanian (n = 4) is still too small for meaningful statistical comparison.

Overall, the patterns observed and tested here suggest that the high amount of phylogenetic information preserved in the Jehol and Late Cretaceous Gobi cannot be attributed alone to sampling intensity. In the fossil record of non-avian theropods, even though there is a lack of statistically significant differences between the Gobi and Late Cretaceous deposits of North America, the Djadokhta and Baruungoyot Formations still preserve an assemblage of species that possess higher quantities of phylogenetic information. In the less complete fossil records of Mesozoic birds and squamates, the extreme differences we observe between lagerstätten deposits (conventional: Jehol; unconventional: Gobi) and the Late Cretaceous Western Interior of North America illustrate that unique taphonomic windows and/or heightened alpha diversity of these taxonomic groups during deposition could be better explanations for the relatively high amounts of phylogenetic information, rather than increased sample size. For squamates, this case study aligns with what we observe in comparisons of CCM2 distributions among different lagerstätten deposits that contain squamates (**Fig 5**), in that taphonomy and depositional setting appear to be primary controls on the completeness of the fossil record and the amount of phylogenetic information preserved.

### The “lagerstätten effect” on taxon selection in phylogenetic analyses: Supply vs. demand

We have shown that the preservation of phylogenetic information among different groups in a fossil assemblage can be both non-uniform (as seen in the Jehol Biota, **Fig 2A**) and uniform (as seen in the Campanian Gobi Desert, **Fig 2B**). We have also shown that the continental (**Fig 3A–3C**) and global (**Fig 4 and S2, S3 Files**) effects of exceptional preservation of phylogenetic information in a fossil assemblage can be different depending on the quality of the fossil record of individual constituent groups (**Fig 1**). Our results also show that there can be differing effects on taxon selection in phylogenetic analyses (**Fig 3D–3I**) by exceptionally-preserved fossil assemblage constituents from a given geological unit (or units, as in the Jehol and Campanian Gobi) depending on the quality of their respective fossil records.

In non-avian theropods, which preserve by far the highest amount of phylogenetic data of the three groups and showcase more complete records on multiple continents, the Jehol and Campanian Gobi Desert contribute nearly equally to the total number of taxa used in the TWiG dataset (**Fig 3D and 3G**). The presence of numerous, highly phylogenetically-complete taxa, both in Asia outside of the Jehol/Campanian Gobi Desert assemblages and in North America suggests that there is a high-quality supply of theropod taxa from the global dataset, such that there is less demand to incorporate a larger portion of the 62 non-avian theropod taxa described from the Jehol and the Campanian Gobi Desert into broad phylogenetic analyses. Therefore, interpretations of the phylogenetic relationships of non-avian theropod dinosaurs may be less vulnerable to biases related to the “lagerstätten effect” than in Mesozoic birds and squamates.

The global preservation of phylogenetic information in the fossil record of Mesozoic birds is significantly poorer than that of non-avian theropods (**Fig 1D**). This means that there is proportionally a less high-quality supply of bird fossils to assess phylogenetic relationships, therefore increasing the demand on the taxonomically and phylogenetically diverse Jehol bird assemblage to provide structure to the Mesozoic bird tree of life. This bias is apparent with 31.04% of the avian portion of the TWiG dataset being made up of Jehol taxa (**Fig 3E**), by far the largest contribution of any Mesozoic bird assemblage. The Campanian Gobi Desert avian assemblage is represented in the TWiG dataset by a single taxon, *Gobipteryx*, reflecting the lack of “lagerstätten effect” generated from the small but relatively complete bird taxa therein. These results corroborate previous assessments of the outsized effect that the diverse assemblage of fossil birds in the Jehol exerts over the Mesozoic avian fossil record [11, 68].

Despite having the largest sample size (n = 797) and spanning the widest range of time (Middle Triassic to Late Pleistocene) of the three surveyed groups, the fossil record of squamates contains a distribution of CCM2 values with the lowest median value (11.38%, **Fig 1D**) and a high concentration of species with CCM2 scores below 20% (522 out of 797 taxa). Similarly to Mesozoic birds, this means that the squamate fossil record contains a relatively small supply of more phylogenetically-complete fossils to incorporate into phylogenetic analyses, thus increasing demand on deposits of exceptional preservation to fill out the structure of the squamate tree of life in geologic time (**Figs 3F, 3I and 5**). Even though there can be an abundance of taxa and remarkable diversity of species in other lagerstätten deposits containing squamates (**Fig 5**), the unique combination of fossil lizard completeness and phylogenetic diversity from the Campanian Gobi Desert meets the demand of filling gaps in a largely incomplete fossil record by providing structure to numerous branches from disparate portions of squamate phylogenetic trees (**Figs 3I and 5E**). Thus, in terms of preserved phylogenetic information content, the Campanian Gobi Desert represents an aeolian lagerstätten deposit that profoundly effects the global picture of squamate fossil record completeness (**Fig 4**), and the structure of squamate evolutionary relationships (**Figs 3I and 5**).

### Quantifying the “lagerstätten effect” via a phylogenetic lens

Our study proposes a new criterion, phylogenetic information content, through which to consider the impact of lagerstätten on our understanding of evolutionary history. Comparing the amount of phylogenetic information in the fossil records of non-avian theropod dinosaurs, Mesozoic birds, and squamates reveals the potential for higher resilience to the “lagerstätten effect” in the more complete record of non-avian theropods, as well as corroborating previous observations of how much our understanding of the Mesozoic bird fossil record is filtered through the diverse Jehol assemblage. But most strikingly, in surveying the completeness of the global fossil record of squamates, we found that the assemblage of 50 highly complete species of lizard from the Upper Cretaceous Djadokhta and Baruungoyot formations of the Gobi Desert exert an anomalously large influence over the availability of phylogenetic character data, as well as taxon selection in phylogenetic analyses. Our unexpected findings from aeolian-dominant facies also show that the potential to preserve a high quantity and quality of evolutionary information is not necessarily restricted to traditional lagerstätten-style depositional settings. Quantifying the “lagerstätten effect” through this phylogenetic lens expands our capacity to maximize the extraordinary wealth of existing paleobiological information available in the rock record. But perhaps more critically, assessing the evolutionary value of exceptional preservation helps to identify gaps in the fossil record that we can prioritize in future sampling endeavors, improving our understanding of biological patterns through time and space.

## Supporting information

S1 File Data used in this study. Excel workbook of fossil occurrence data and CCM2 scores used in this study.
(XLSX)

S2 File Statistical tests. Excel workbook of results of statistical tests run for this study.
(XLSX)

S3 File Supporting information. Document containing supplementary discussion, supplementary **S1-S7 Figs**, and SI references.
(DOCX)
