# Supplementary material for: Quantifying the effects of exceptional fossil preservation on the global availability of phylogenetic data in deep time
Source: PLoS One. 2024 Feb 14;19(2):e0297637. doi: 10.1371/journal.pone.0297637 (PMC10866489; doi:10.1371/journal.pone.0297637)
Supplement: S3 File — Document containing supplementary discussion, supplementary S1-S7 Figs, and SI references. (DOCX) [file pone.0297637.s003.docx]

***PLOS ONE***

**Supporting Information for**

**Quantifying the effects of exceptional fossil preservation on the global availability of phylogenetic data in deep time**

C. Henrik Woolley^1,2^*, David J. Bottjer^2^, Frank A. Corsetti^2^, Nathan D. Smith^1^

^1^ Dinosaur Institute, Natural History Museum of Los Angeles County, 900 Exposition Blvd, Los Angeles, California, USA 90007

^2^ Department of Earth Sciences, University of Southern California, 3651 Trousdale Pkwy, ZHS 119, Los Angeles, California, USA 90089

*Corresponding Author: C. Henrik Woolley.

**Email:**  chwoolle@usc.edu

**This PDF file includes:**

Supporting text

Figures S1 to S7

SI References

**Other supporting materials for this manuscript include the following:**

Datasets S1 to S2

Supporting Information Text

**Depositional setting and completeness**

Pairwise statistical comparisons between median CCM2 percentages (Mann-Whitney U) and cumulative distribution (Kolmogorov-Smirnov) among these distributions reveal several patterns. First, the median and distribution shape of the CCM2 values from lacustrine environments differ from those seen in fluvial channel and karst environments. Second, the median and distribution shape of CCM2 values in karst environments also differs from those of marine and coastal lagoon depositional settings. Third, the median and distribution shape of CCM2 values in marine depositional environments are statistically significantly different from those found in all three fluvial depositional categories (fluvial floodplain, fluvial channel, fluvial indet.).

**Gobi Desert Lizards Compared to Other Lizards**

Fossil preservation can be dependent not only on depositional environment, but also body plan. Because only lizards have been described from the Djadokhta/Baruungoyot formations, it is important to consider their collective completeness compared alongside lizard fossils from other depositional settings with high completeness without the squamates with extremely different body plans (snakes, mosasaurs, amphisbaenians). If we compare the completeness of lizards found in the Late Cretaceous Gobi Desert to lizards found across geologic time in lacustrine and lagoonal environments (**Fig. S6**) we observe statistically significant differences in median CCM2 ( Gobi + Lacustrine: α = 0.05; p = 0.0022; Gobi + Lagoon: α = 0.05; p = 0.0002) and distribution shape (Gobi + Lacustrine:α = 0.05; p = 0.000019; Gobi + Lagoon: α = 0.05; p = 0.0017). These results show that even though we can expect the best physical preservation and most phylogenetically complete lizards (e.g., *Saniwa ensidens* in lacustrine settings with CCM2 = 96.28%; *Huehuecuetzpalli mixtecus* in lagoonal settings with CCM2 = 72.94%) in low-energy, potentially hypoxic or anoxic depositional settings, there can be a high average amount of phylogenetic information preserved in settings that do not meet those criteria (median CCM2 for Gobi desert: 54.65%; median CCM2 for lacustrine: 30.31%; median CCM2 for lagoonal: 35.08%).

**Supplementary Figures**

**
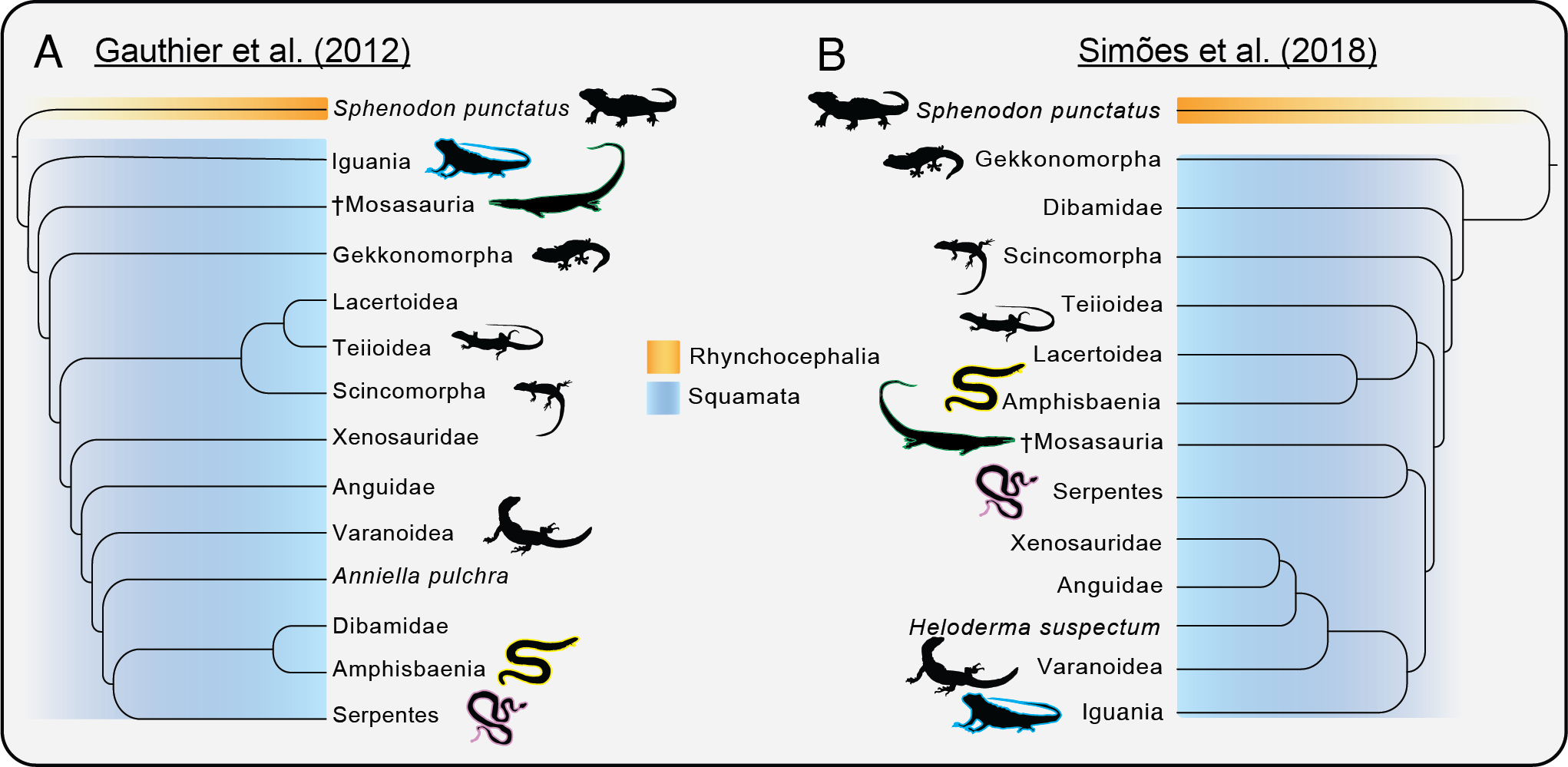
**

**Fig. S1**. Summary of morphology-based hypotheses of squamate evolutionary relationships, whose character datasets were used for the Character Completeness Metric 2 (CCM2) in this study. A) Gauthier et al. (2012) hypothesis. B) Simões et al. (2018) hypothesis. Silhouettes with colored outlines indicate major squamate groups whose phylogenetic positions differ significantly among the two hypotheses. All silhouettes traced from publicly available images at [www.phylopic.org](http://www.phylopic.org).

**
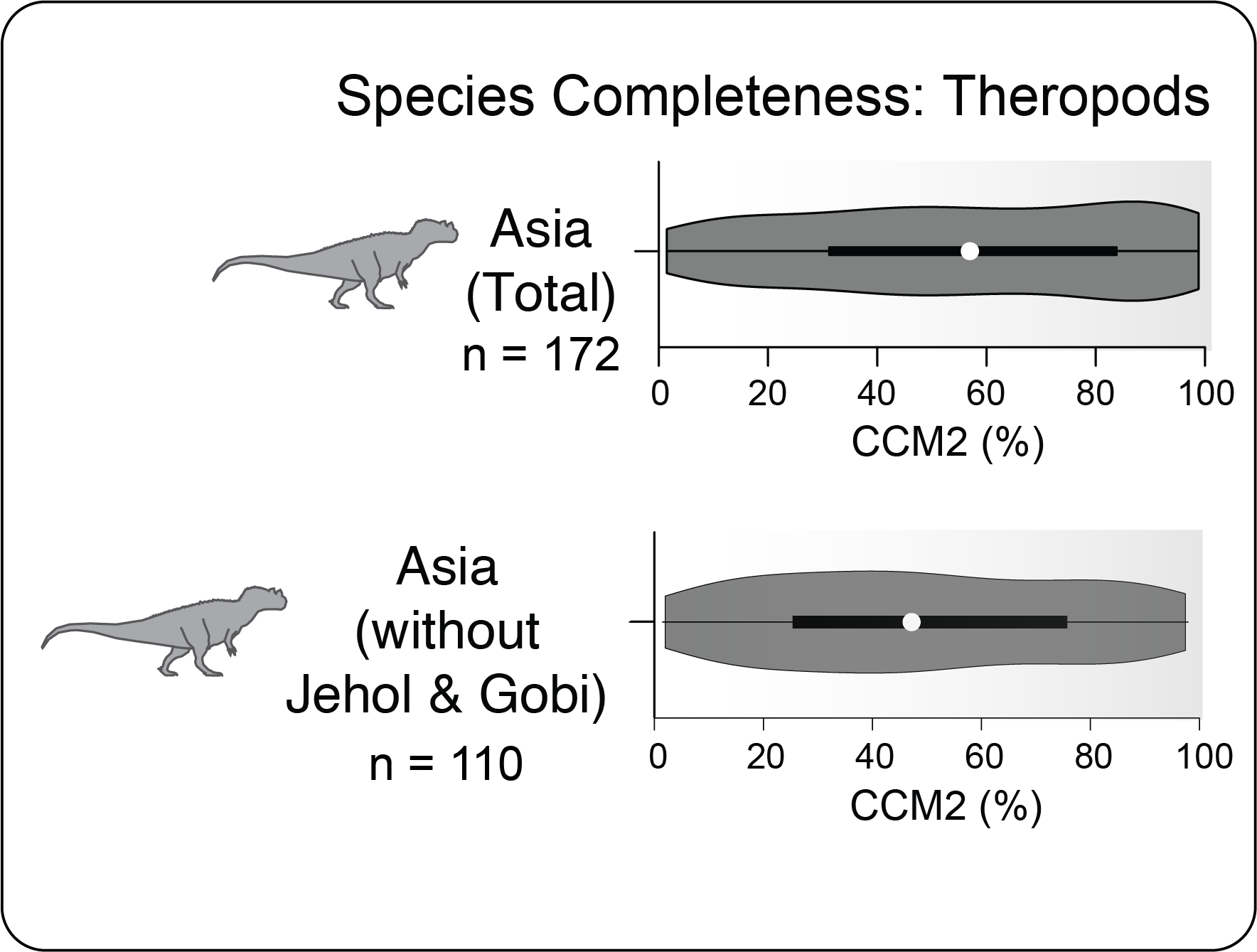
**

**Fig. S2**. Distribution of Character Completeness Metric 2 (CCM2) in non-avian theropod dinosaurs from the total Asia dataset (top) and without the 62 surveyed taxa from the Jehol Biota and Campanian Gobi Desert (bottom). *White dot*: median; *black bar*: interquartile range; *black line*: 95% confidence interval.

**
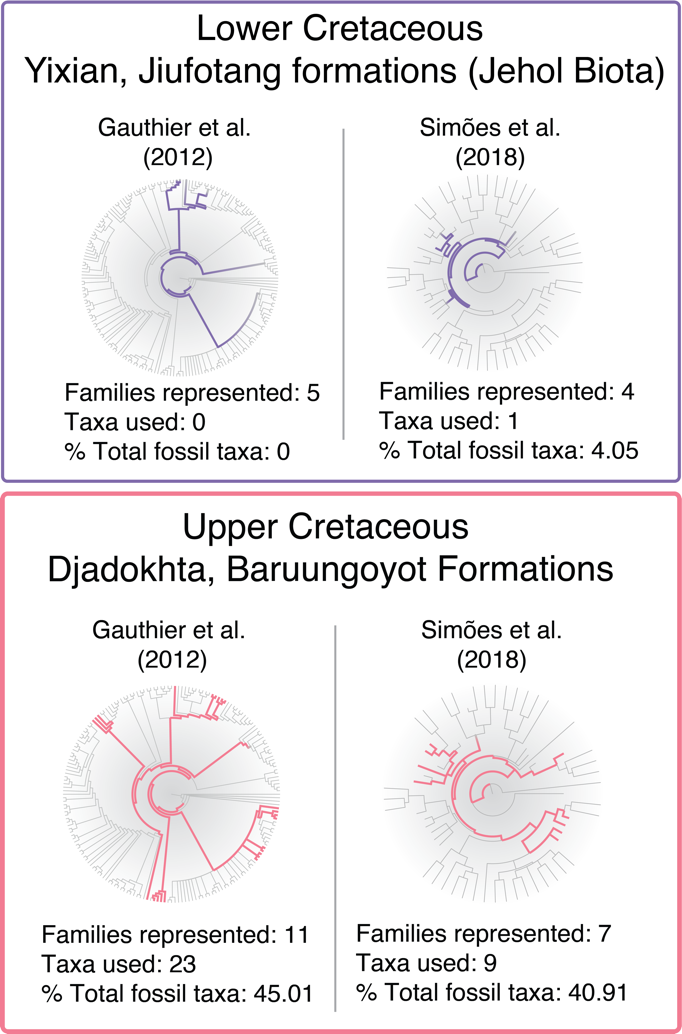
**

**Fig. S3.** Comparisons of squamate families represented in the Gauthier et al. (2012) and Simões et al. (2018) datasets from the Jehol Biota (top) and Campanian Gobi Desert (bottom).


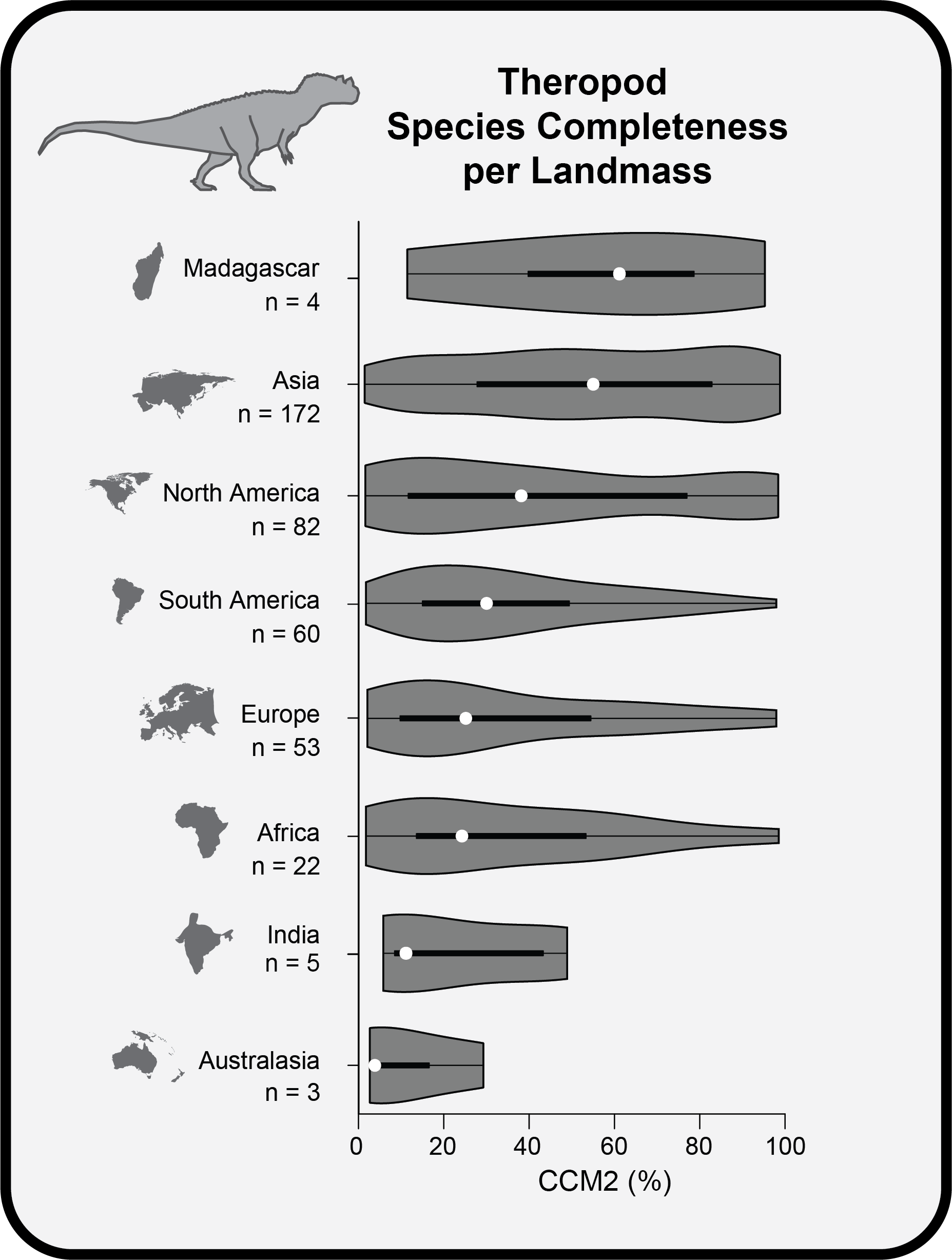


**Fig. S4**. Violin plots of the distribution of non-avian theropod CCM2 values per sampled landmass. *White dot*: median; *black bar*: interquartile range; *black line*: 95% confidence interval.


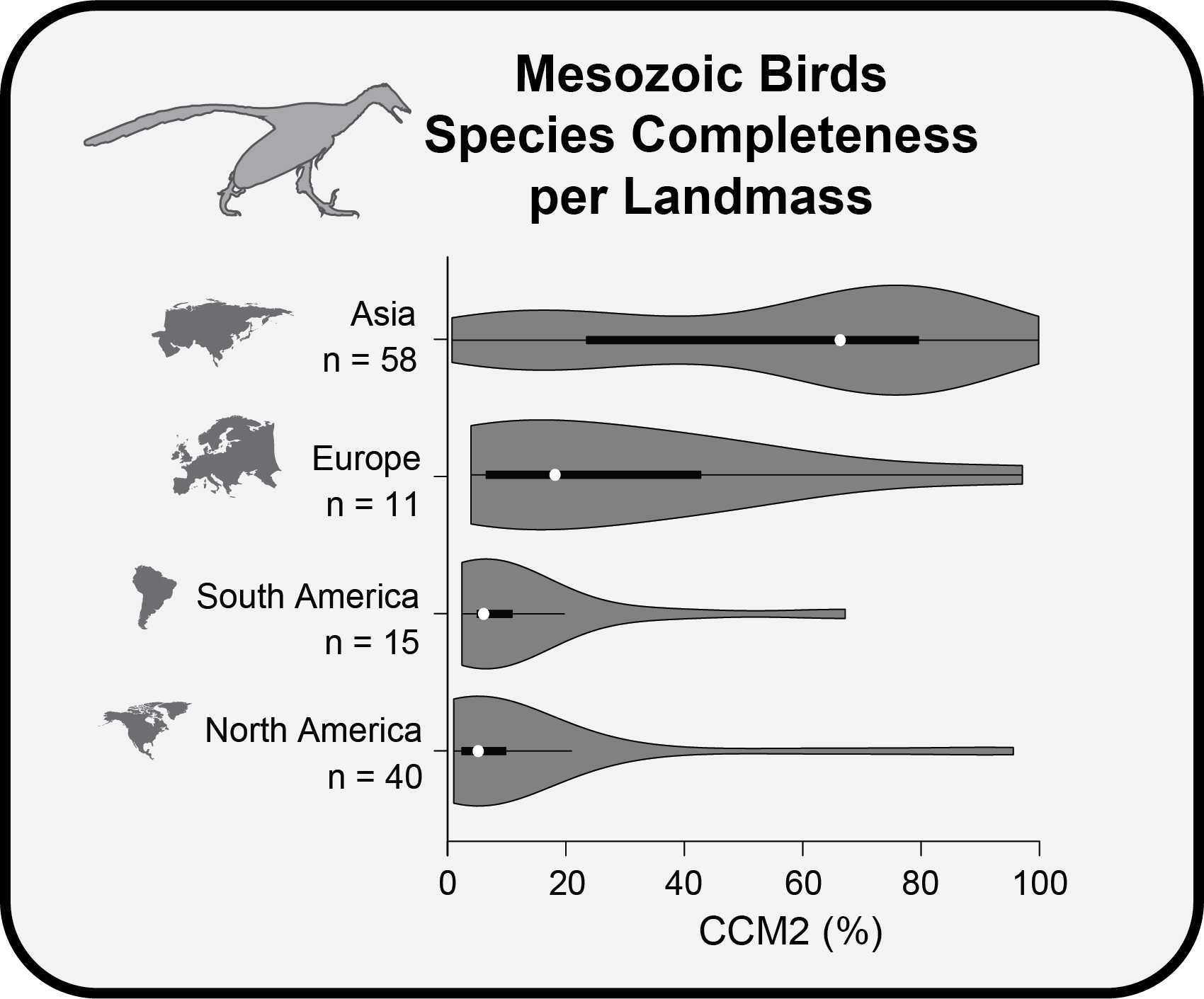


**Fig. S5**. Violin plots of the distribution of Mesozoic bird CCM2 values per sampled landmass. Not pictured: Antarctica (n = 2); Australasia (n = 1); Madagascar (n = 1). *White dot*: median; *black bar*: interquartile range; *black line*: 95% confidence interval.


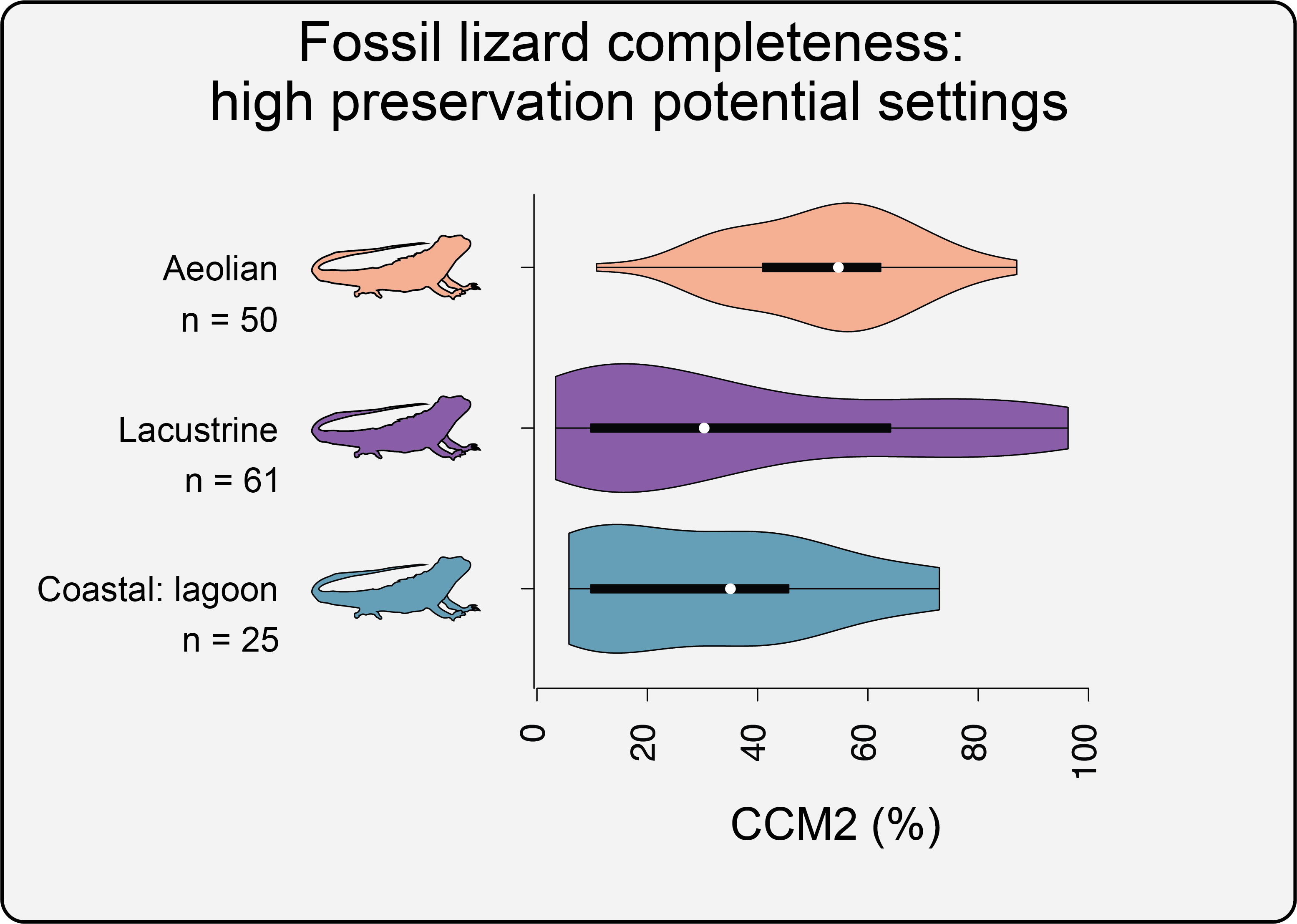


**Fig. S6**. Comparisons of distributions of fossil lizard CCM2 values from high-preservation potential depositional environments. *White dot*: median; *black bar*: interquartile range; *black line*: 95% confidence interval.


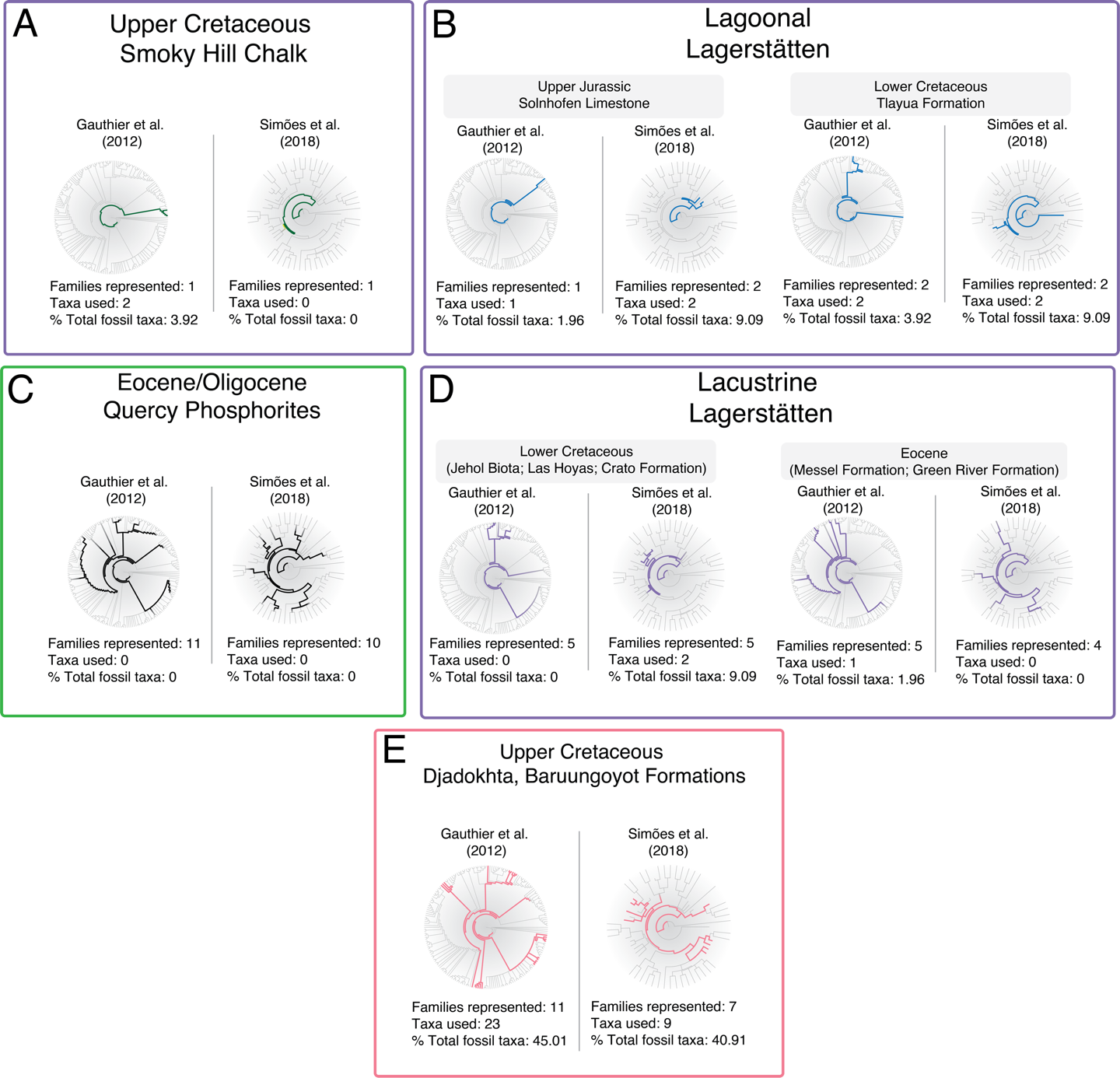


**Fig. S7**. Comparisons of squamate families represented in the Gauthier et al. (2012) and Simões et al. (2018) datasets from each lagerstätten deposit surveyed in this study. **A**. Smoky Hill Chalk. **B**. Lagoonal lagerstätten deposits. **C**. Quercy Phosphorites. **D**. Lacustrine lagerstätten deposits. **E**. Djadokhta and Baruungoyot Formations.

**SI Data**

**Supplementary Data S1**. Excel spreadsheet of taxon, specimen, and locality information from the Paleobiology Database and from primary literature.

**Supplementary Data S2**. Excel workbook of statistical tests performed in this study.

**SI References**

***Triassic Squamate References***

1. S. Renesto, R. Posenato, A new lepidosauromorph reptile from the Middle Triassic of the Dolomites (Northern Italy). *Rivista Italiana di Paleontologia e Stratigrafia* **109** (2003).

2. T. R. Simões *et al.*, The origin of squamates revealed by a Middle Triassic lizard from the Italian Alps. *Nature* **557**, 706-709 (2018).

***Jurassic Squamate References***

1. M. W. Caldwell, R. L. Nydam, A. Palci, S. Apesteguía, The oldest known snakes from the Middle Jurassic-Lower Cretaceous provide insights on snake evolution. *Nature communications* **6**, 1-11 (2015).

2. M. Cocude-Michel, Les Sauriens des calcaires lithographiques de Bavière, d’âge portlandien inférieur. *Bulletin de la Société Géologique de France*, 707-710 (1960).

3. J. L. Conrad, A new lizard (Squamata) was the last meal of *Compsognathus* (Theropoda: Dinosauria) and is a holotype in a holotype. *Zoological Journal of the Linnean Society* **183**, 584-634 (2018).

4. L. Dong, Y. Wang, L. Mou, G. Zhang, S. E. Evans, A new Jurassic lizard from China. *Geodiversitas* **41**, 623-641 (2019).

5. S. Evans, Crown group lizards (Reptilia, Squamata) from the middle Jurassic of the British Isles. *Palaeontographica Abteilung A-Stuttgart-* **250**, 123-154 (1998).

6. S. E. Evans, G. Prasad, B. Manhas, Fossil lizards from the Jurassic Kota formation of India. *Journal of Vertebrate Paleontology* **22**, 299-312 (2002).

7. N. J. Mateer, Osteology of the Jurassic Lizard *Ardeosaurus brevipes* (Meyer). *Palaeontology* **25**, 461-469 (1982).

8. T. R. Simões (2018) Diapsid Phylogeny and the Origin and Early Evolution of Squamates. in *Biological Sciences* (University of Alberta, Edmonton), p 415.

9. T. R. Simões, M. W. Caldwell, R. L. Nydam, P. Jiménez-Huidobro, Osteology, phylogeny, and functional morphology of two Jurassic lizard species and the early evolution of scansoriality in geckoes. *Zoological Journal of the Linnean Society* **180**, 216-241 (2017).

***Early Cretaceous Squamate References***

1. E. Arnold, D. Azar, I. Ineich, A. Nel, The oldest reptile in amber: a 120 million year old lizard from Lebanon. *Journal of Zoology* **258**, 7-10 (2002).

2. J. S. Bittencourt, T. R. Simões, M. W. Caldwell, M. C. Langer, Discovery of the oldest South American fossil lizard illustrates the cosmopolitanism of early South American squamates. *Communications biology* **3**, 1-11 (2020).

3. A. Bolet, S. E. Evans, A new lizard from the Early Cretaceous of Catalonia (Spain), and the Mesozoic lizards of the Iberian Peninsula. *Cretaceous Research* **31**, 447-457 (2010).

4. A. Bolet, S. E. Evans, A tiny lizard (Lepidosauria, Squamata) from the Lower Cretaceous of Spain. *Palaeontology* **55**, 491-500 (2012).

5. M. W. Caldwell *et al.*, *Tetrapodophis amplectus* is not a snake: re-assessment of the osteology, phylogeny and functional morphology of an Early Cretaceous dolichosaurid lizard. *Journal of Systematic Palaeontology* **19**, 893-952 (2021).

6. J. L. Conrad, J. D. Daza, Naming and rediagnosing the Cretaceous gekkonomorph (Reptilia, Squamata) from Öösh (Övörkhangai, Mongolia). *Journal of Vertebrate Paleontology* **35**, e980891 (2015).

7. J. D. Daza, V. R. Alifanov, A. M. Bauer, A redescription and phylogenetic reinterpretation of the fossil lizard *Hoburogekko suchanovi* Alifanov, 1989 (Squamata, Gekkota), from the Early Cretaceous of Mongolia. *Journal of Vertebrate Paleontology* **32**, 1303-1312 (2012).

8. L. Dong, Y. Wang, S. E. Evans, A new lizard (Reptilia: Squamata) from the Lower Cretaceous Yixian Formation of China, with a taxonomic revision of Yabeinosaurus. *Cretaceous Research* **72**, 161-171 (2017).

9. R. Estes, "Sauria terrestria, Amphisbaenia" in Handbuch der Paläoherpetologie/Encyclopedia of Paleoherpetology*,* P. Wellnhofer, Ed. (Gustav Fischer Verlag, Stuttgart; New York, 1983), chap. 10A.

10. S. Evans, L. Barbadillo, An unusual lizard (Reptilia: Squamata) from the Early Cretaceous of Las Hoyas, Spain. *Zoological Journal of the Linnean Society* **124**, 235-265 (1998).

11. S. Evans, L. Barbadillo, A short-limbed lizard from the Lower Cretaceous of Spain. *Special Papers in Palaeontology* **60**, 73-85 (1999).

12. S. Evans, B. Searle, Lepidosaurian reptiles from the Purbeck limestone Group of Dorset, southern England. *Special Papers in Palaeontology* **68**, 145-160 (2002).

13. S. E. Evans, J. Barbadillo, Early Cretaceous lizards from Las Hoyas, Spain. *Zoological Journal of the Linnean Society* **119**, 23-49 (1997).

14. S. E. Evans, L. J. Barbadillo, The Early Cretaceous lizards of Montsec (Catalonia, Spain). *Treballs del Museu de Geologia de Barcelona*, 5-13 (1996).

15. S. E. Evans, L. J. Barbadillo, The lizard *Rubiessaurus* Gómez Pallerola, 1979 from the Lower Cretaceous of Catalonia (Montsec, Lleida, Spain). *Treballs del Museu de Geologia de Barcelona*, 5-10 (1998).

16. S. E. Evans, D. C. Chure, Paramacellodid lizard skulls from the Jurassic Morrison Formation at Dinosaur national monument, Utah. *Journal of Vertebrate Paleontology* **18**, 99-114 (1998).

17. S. E. Evans, M. E. Jones, R. Matsumoto, A new lizard skull from the Purbeck Limestone Group (Lower Cretaceous) of England. *Bulletin de la Société géologique de France* **183**, 517-524 (2012).

18. S. E. Evans, M. Manabe, Early Cretaceous lizards from the Okurodani Formation of Japan. *Geobios* **32**, 889-899 (1999).

19. S. E. Evans, M. Manabe, An early herbivorous lizard from the Lower Cretaceous of Japan. *Palaeontology* **51**, 487-498 (2008).

20. S. E. Evans, M. Manabe, M. Noro, S. Isaji, M. Yamaguchi, A long‐bodied lizard from the Lower Cretaceous of Japan. *Palaeontology* **49**, 1143-1165 (2006).

21. S. E. Evans, R. Matsumoto, An assemblage of lizards from the Early Cretaceous of Japan. *Palaeontologia Electronica* **18** (2015).

22. S. E. Evans, P. Raia, C. Barbera, New lizards and rhynchocephalians from the Lower Cretaceous of southern Italy. *Acta Palaeontologica Polonica* **49** (2004).

23. S. E. Evans, P. Raia, C. Barbera, The lower Cretaceous lizard genus *Chometokadmon* from Italy. *Cretaceous Research* **27**, 673-683 (2006).

24. S. E. Evans, Y. Wang, The early cretaceous lizard *Dalinghosaurus* from China. *Acta Palaeontologica Polonica* **50**, 725-742 (2005).

25. S. E. Evans, Y. Wang, A new lizard (Reptilia: Squamata) with exquisite preservation of soft tissue from the Lower Cretaceous of Inner Mongolia, China. *Journal of Systematic Palaeontology* **8**, 81-95 (2010).

26. S. E. Evans, Y. Wang, New material of the Early Cretaceous lizard *Yabeinosaurus* from China. *Cretaceous Research* **34**, 48-60 (2012).

27. S. E. Evans, Y. Wang, C. Li, The Early Cretaceous lizard genus *Yabeinosaurus* from China: resolving an enigma. *Journal of Systematic Palaeontology* **3**, 319-335 (2005).

28. S. E. Evans, Y. Yabumoto, A lizard from the Early Cretaceous Crato Formation, Araripe Basin, Brazil. *Neues Jahrbuch fur Geologie und Palaontologie Monatshefte*, 349-364 (1998).

29. R. Hoffstetter, Un serpent terrestre dans le Crétacé inférieur du Sahara. *Bulletin de la Société géologique de France* **7**, 897-902 (1959).

30. R. Hoffstetter, Coup d’œil sur les sauriens (=lacertiliens) des couches de Purbeck (Jurassique Supérieur d’Angleterre). *Colloques Internationaux du Centre National de la Recherche Scientifique* **163**, 349-371 (1967).

31. A. Houssaye *et al.*, A new varanoid squamate from the Early Cretaceous (Barremian–Aptian) of Burgos, Spain. *Cretaceous Research* **41**, 127-135 (2013).

32. T. Ikeda, H. Ota, H. Saegusa, A new fossil lizard from the Lower Cretaceous Sasayama Group of Hyogo prefecture, western Honshu, Japan. *Journal of Vertebrate Paleontology* **35**, e885032 (2015).

33. G. Keqin, C. Zhengwu, A new lizard from the Lower Cretaceous of Shandong, China. *Journal of Vertebrate Paleontology* **19**, 456-465 (1999).

34. J. Li, A new lizard from Late Jurassic of Subei, Gansu. *Vertebrata PalAsiatica* **23**, 13-18 (1985).

35. P.-P. Li, K.-Q. Gao, L.-H. Hou, X. Xu, A gliding lizard from the Early Cretaceous of China. *Proceedings of the National Academy of Sciences* **104**, 5507-5509 (2007).

36. D. M. Martill, H. Tischlinger, N. R. Longrich, A four-legged snake from the Early Cretaceous of Gondwana. *Science* **349**, 416-419 (2015).

37. R. L. Nydam, Polyglyphanodontinae (Squamata: Teiidae) from the medial and Late Cretaceous: New records from Utah, U.S.A. and Baja California del Norte, Mexico. *Vertebrate Paleontology in Utah* **99**, 303 (1999).

38. R. L. Nydam, A new taxon of helodermatid-like lizard from the Albian–Cenomanian of Utah. *Journal of vertebrate Paleontology* **20**, 285-294 (2000).

39. R. L. Nydam, Lizards of the Mussentuchit local fauna (Albian–Cenomanian boundary) and comments on the evolution of the Cretaceous lizard fauna of North America. *Journal of Vertebrate Paleontology* **22**, 645-660 (2002).

40. R. L. Nydam, R. L. Cifelli, Lizards from the Lower Cretaceous (Aptian–Albian) Antlers and Cloverly Formations. *Journal of Vertebrate Paleontology* **22**, 286-298 (2002).

41. R. L. Nydam, R. L. Cifelli, A new teiid lizard from the Cedar Mountain Formation (Albian–Cenomanian boundary) of Utah. *Journal of Vertebrate Paleontology* **22**, 276-285 (2002).

42. J. O’Connor *et al.*, *Microraptor* with ingested lizard suggests non-specialized digestive function. *Current Biology* **29**, 2423-2429. e2422 (2019).

43. D. R. Prothero, R. Estes, Late Jurassic lizards from Como Bluff, Wyoming and their palaeobiogeographic significance. *Nature* **286**, 484-486 (1980).

44. V.-H. Reynoso, *Huehuecuetzpalli mixtecus* gen. et sp. nov: a basal squamate (Reptilia) from the Early Cretaceous of Tepexi de Rodríguez, Central México. *Philosophical Transactions of the Royal Society of London. Series B: Biological Sciences* **353**, 477-500 (1998).

45. V.-H. Reynoso, G. Callison, A new scincomorph lizard from the Early Cretaceous of Puebla, México. *Zoological Journal of the Linnean Society* **130**, 183-212 (2000).

46. A. Richter, M. Wuttke, Analysing the taphonomy of Mesozoic lizard aggregates from Uña (eastern Spain) by X-ray controlled decay experiments. *Palaeobiodiversity and Palaeoenvironments* **92**, 5-28 (2012).

47. T. Shikama, *Teilhardosaurus* and *Endotherium*, new Jurassic Reptilia and Mammalia from the Husin coal-field, south Manchuria. *Proceedings of the Japan Academy* **23**, 76-84 (1947).

48. J. Shu'an, A new early cretaceous lizard with well–preserved scale impressions from western liaoning, China. *Progress in Natural Science* **15**, 162-168 (2005).

49. T. R. Simões, Redescription of *Tijubina pontei*, an Early Cretaceous lizard (Reptilia; Squamata) from the Crato Formation of Brazil. *Anais da Academia Brasileira de Ciências* **84**, 79-94 (2012).

50. T. R. Simões, M. W. Caldwell, A. W. Kellner, A new Early Cretaceous lizard species from Brazil, and the phylogenetic position of the oldest known South American squamates. *Journal of Systematic Palaeontology* **13**, 601-614 (2015).

***Cenomanian-Santonian Squamate References***

1. A. Albino, J. D. Carrillo-Briceño, J. M. Neenan, An enigmatic aquatic snake from the Cenomanian of Northern South America. *PeerJ* **4**, e2027 (2016).

2. M. T. Antunes, *O Neocretácico e o Cenozóico do litoral de Angola* (Junta de Investigações do Ultramar, Lisbon, 1964).

3. S. Apesteguía, J. D. Daza, T. R. Simões, J. C. Rage, The first iguanian lizard from the Mesozoic of Africa. *Royal Society Open Science* **3**, 160462 (2016).

4. S. Apesteguía, H. Zaher, A Cretaceous terrestrial snake with robust hindlimbs and a sacrum. *Nature* **440**, 1037-1040 (2006).

5. N. Bardet, X. P. Suberbiola, The basal mosasaurid *Halisaurus sternbergii* from the Late Cretaceous of Kansas (North America): a review of the Uppsala type specimen. *Comptes Rendus de l'Académie des Sciences-Series IIA-Earth and Planetary Science* **332**, 395-402 (2001).

6. N. Bardet, X. P. Suberbiola, N.-E. Jalil, A new mosasauroid (Squamata) from the Late Cretaceous (Turonian) of Morocco. *Comptes Rendus Palevol* **2**, 607-616 (2003).

7. G. Bell, M. Polcyn, *Dallasaurus turneri*, a new primitive mosasauroid from the Middle Turonian of Texas and comments on the phylogeny of Mosasauridae (Squamata). *Netherlands Journal of Geosciences* **84**, 177-194 (2005).

8. S. J. Bolkay, A new snakelike reptile from the Lower Cretaceous (Neocomian) of Bilek-Selista (East Hercegovina). *Glasnika Zemaljskog Mujeza u Bosni I Hercegovini* **37**, 125-136 (1925).

9. M. W. Caldwell, Description and phylogenetic relationships of a new species of *Coniasaurus* Owen, 1850 (Squamata). *Journal of Vertebrate Paleontology* **19**, 438-455 (1999).

10. M. W. Caldwell, On the aquatic squamate *Dolichosaurus longicollis* Owen, 1850 (Cenomanian, Upper Cretaceous), and the evolution of elongate necks in squamates. *Journal of Vertebrate Paleontology* **20**, 720-735 (2001).

11. M. W. Caldwell, A. Albino, Exceptionally preserved skeletons of the Cretaceous snake *Dinilysia patagonica* Woodward, 1901. *Journal of Vertebrate Paleontology* **22**, 861-866 (2003).

12. M. W. Caldwell, R. L. Carroll, H. Kaiser, The pectoral girdle and forelimb of *Carsosaurus marchesetti* (Aigialosauridae), with a preliminary phylogenetic analysis of mosasauroids and varanoids. *Journal of Vertebrate Paleontology* **15**, 516-531 (1995).

13. M. W. Caldwell, J. A. Cooper, Redescription, palaeobiogeography and palaeoecology of *Coniasaurus crassidens* Owen, 1850 (Squamata) from the Lower Chalk (Cretaceous; Cenomanian) of Southeastern England. *Zoological Journal of the Linnean Society* **127**, 423-452 (1999).

14. M. W. Caldwell, T. Konishi, I. Obata, K. Muramoto, A new species of *Taniwhasaurus* (Mosasauridae, Tylosaurinae) from the upper santonian-lower campanian (Upper Cretaceous) of Hokkaido, Japan. *Journal of Vertebrate Paleontology* **28**, 339-348 (2008).

15. M. W. Caldwell, M. S. Lee, A snake with legs from the marine Cretaceous of the Middle East. *Nature* **386**, 705-709 (1997).

16. M. W. Caldwell, M. S. Lee, Reevaluation of the Cretaceous marine lizard *Acteosaurus crassicostatus* Calligaris, 1993. *Journal of Paleontology* **78**, 617-619 (2004).

17. M. W. Caldwell, A. Palci, A new basal mosasauroid from the Cenomanian (U. Cretaceous) of Slovenia with a review of mosasauroid phylogeny and evolution. *Journal of Vertebrate Paleontology* **27**, 863-880 (2007).

18. M. W. Caldwell, A. Palci, A new species of marine ophidiomorph lizard, *Adriosaurus skrbinensis*, from the Upper Cretaceous of Slovenia. *Journal of Vertebrate Paleontology* **30**, 747-755 (2010).

19. C. DalSasso, G. Pinna, *Aphanizocnemus libanensis n. gen. n. sp., a new dolichosaur (Reptilia, Varanoidea) from the Upper Cretaceous of Lebanon* (Società Italiana di Scienze Naturali, 1997).

20. J. D. Daza *et al.*, An enigmatic miniaturized and attenuate whole lizard from the Mid-Cretaceous amber of Myanmar. *Breviora* **563**, 1-18 (2018).

21. A. R. Dutchak, M. W. Caldwell, Redescription of *Aigialosaurus dalmaticus* Kramberger, 1892, a Cenomanian mosasauroid lizard from Hvar Island, Croatia. *Canadian Journal of Earth Sciences* **43**, 1821-1834 (2006).

22. A. R. Dutchak, M. W. Caldwell, A redescription of *Aigialosaurus* (= *Opetiosaurus*) *bucchichi* (Kornhuber, 1901)(Squamata: Aigialosauridae) with comments on mosasauroid systematics. *Journal of Vertebrate Paleontology* **29**, 437-452 (2009).

23. K. Gao, R. Fox, New teiid lizards from the Upper Cretaceous Oldman Formation (Judithian) of southeastern Alberta, Canada, with a review of the Cretaceous record of teiids. *Annals of the Carnegie Museum* **60**, 145-162 (1991).

24. K. Gao, R. C. Fox, *Taxonomy and evolution of Late Cretaceous lizards (Reptilia: Squamata) from western Canada* (Carnegie Museum of Natural History, 1996).

25. A. Haber, M. Polcyn, A new marine varanoid from the Cenomanian of the Middle East. *Netherlands Journal of Geosciences* **84**, 247-255 (2005).

26. A. S. Hsiou, A. M. Albino, M. A. Medeiros, R. A. Santos, The oldest Brazilian snakes from the Cenomanian (early Late Cretaceous). *Acta Palaeontologica Polonica* **59**, 635-642 (2013).

27. C. G. Klein, N. R. Longrich, N. Ibrahim, S. Zouhri, D. M. Martill, A new basal snake from the mid-Cretaceous of Morocco. *Cretaceous Research* **72**, 134-141 (2017).

28. T. Konishi, M. W. Caldwell, New specimens of *Platecarpus planifrons* (Cope, 1874)(Squamata: Mosasauridae) and a revised taxonomy of the genus. *Journal of Vertebrate Paleontology* **27**, 59-72 (2007).

29. M. Lee, M. Caldwell, J. Scanlon, A second primitive marine snake: *Pachyophis woodwardi* from the Cretaceous of Bosnia-Herzegovina. *Journal of Zoology* **248**, 509-520 (1999).

30. M. S. Lee, M. W. Caldwell, *Adriosaurus* and the affinities of mosasaurs, dolichosaurs, and snakes. *Journal of Paleontology* **74**, 915-937 (2000).

31. M. S. Lee, J. D. Scanlon, The Cretaceous marine squamate *Mesoleptos* and the origin of snakes. *Bulletin of the Natural History Museum: Zoology Series* **68**, 131-142 (2002).

32. J. Lindgren, M. J. Everhart, M. W. Caldwell, Three-dimensionally preserved integument reveals hydrodynamic adaptations in the extinct marine lizard *Ectenosaurus* (Reptilia, Mosasauridae). *PLoS One* **6**, e27343 (2011).

33. J. Lindgren, M. Siverson, *Halisaurus sternbergi*, a small mosasaur with an intercontinental distribution. *Journal of Paleontology* **79**, 763-773 (2005).

34. L. Makádi, A new polyglyphanodontine lizard (Squamata: Borioteiioidea) from the late cretaceous Iharkút locality (Santonian, Hungary). *Cretaceous Research* **46**, 166-176 (2013).

35. L. Makádi (2013) The first known chamopsiid lizard (Squamata) from the upper cretaceous of Europe (Csehbánya Formation; Hungary, Bakony Mts). in *Annales de Paléontologie* (Elsevier), pp 261-274.

36. L. Makádi, M. W. Caldwell, A. Ősi, The first freshwater mosasauroid (Upper Cretaceous, Hungary) and a new clade of basal mosasauroids. *PLoS One* **7**, e51781 (2012).

37. L. Makádi, R. L. Nydam, A new durophagous scincomorphan lizard genus from the Late Cretaceous Iharkút locality (Hungary, Bakony Mts). *Paläontologische Zeitschrift* **89**, 925-941 (2015).

38. O. Mateus *et al.*, Cretaceous amniotes from Angola: dinosaurs, pterosaurs, mosasaurs, plesiosaurs, and turtles. *V Jornadas Internacionales sobre Paleontología de Dinosaurios y su Entorno* (2012).

39. M. C. Mekarski, D. Japundžić, K. Krizmanić, M. W. Caldwell, Description of a new basal mosasauroid from the Late Cretaceous of Croatia, with comments on the evolution of the mosasauroid forelimb. *Journal of Vertebrate Paleontology* **39**, e1577872 (2019).

40. A. Nessov, V. I. Zhegallo, A. O. Averianov (1998) A new locality of Late Cretaceous snakes, mammals and other vertebrates in Africa (western Libya). in *Annales de Paléontologie* (Elsevier), pp 265-274.

41. E. L. Nicholls, D. Meckert, Marine reptiles from the Nanaimo Group (Upper Cretaceous) of Vancouver Island. *Canadian Journal of Earth Sciences* **39**, 1591-1603 (2002).

42. R. L. Nydam, "Lizards and Snakes from the Cenomanian through Campanian of Southern Utah: Filling the Gap in the Fossil Record of Squamata from the Late Cretaceous of the Western Interior of North America" in At the Top of the Grand Staircase: the Late Cretaceous of Southern Utah*,* A. Titus, M. Loewen, Eds. (Indiana University Press Bloomington, Indiana, 2013), pp. 370-423.

43. R. L. Nydam, *Dakotaseps* gen. nov., a replacement name for the lizard genus *Dakotasaurus* Nydam 2013, a junior homonym of the ichnotaxon *Dakotasaurus* Branson and Mehl 1932. *Zootaxa* **3900**, 150-150 (2014).

44. R. L. Nydam, J. G. Eaton, J. Sankey, New taxa of transversely-toothed lizards (Squamata: Scincomorpha) and new information on the evolutionary history of “teiids”. *Journal of Paleontology* **81**, 538-549 (2007).

45. R. L. Nydam, B. M. Fitzpatrick, The occurrence of *Contogenys*-like lizards in the Late Cretaceous and Early Tertiary of the Western Interior of the USA. *Journal of Vertebrate Paleontology* **29**, 677-701 (2009).

46. A. Palci, M. W. Caldwell, Vestigial forelimbs and axial elongation in a 95 million-year-old non-snake squamate. *Journal of Vertebrate Paleontology* **27**, 1-7 (2007).

47. A. Palci, M. W. Caldwell, C. A. Papazzoni, A new genus and subfamily of mosasaurs from the Upper Cretaceous of northern Italy. *Journal of Vertebrate Paleontology* **33**, 599-612 (2013).

48. S. E. Pierce, M. W. Caldwell, Redescription and phylogenetic position of the Adriatic (Upper Cretaceous; Cenomanian) dolichosaur *Pontosaurus* *lesinensis* (Kornhuber, 1873). *Journal of Vertebrate Paleontology* **24**, 373-386 (2004).

49. M. Polcyn, G. Bell, *Russellosaurus coheni* n. gen., n. sp., a 92 million-year-old mosasaur from Texas (USA), and the definition of the parafamily Russellosaurina. *Netherlands Journal of Geosciences* **84**, 321-333 (2005).

50. M. J. Polcyn, M. J. Everhart (2008) Description and phylogenetic analysis of a new species of *Selmasaurus* (Mosasauridae: Plioplatecarpinae) from the Niobrara Chalk of western Kansas. in *Proceedings of the Second Mosasaur Meeting* (Fort Hayes Studies, Special), pp 13-28.

51. J.-C. Rage, F. Escuillié, Un nouveau serpent bipède du Cénomanien (Crétacé). Implications phylétiques. *Comptes Rendus de l'Académie des Sciences-Series IIA-Earth and Planetary Science* **330**, 513-520 (2000).

52. J.-C. Rage, R. Vullo, D. Néraudeau, The mid-Cretaceous snake *Simoliophis rochebrunei* Sauvage, 1880 (Squamata: Ophidia) from its type area (Charentes, southwestern France): Redescription, distribution, and palaeoecology. *Cretaceous Research* **58**, 234-253 (2016).

53. J. C. Rage, D. Néraudeau, A new pachyostotic squamate reptile from the Cenomanian of France. *Palaeontology* **47**, 1195-1210 (2004).

54. D. A. Russell, Systematics and morphology of American mosasaurs. *Yale University Peabody Museum of Natural History Bulletin* **23**, 1-241 (1967).

55. D. A. Russell, The Vertebrate Fauna of the Selma Formation of Alabama: Part VII The Mosasaurs. *Fieldiana: Geology Memoirs* **3**, 365-380 (1970).

56. D. A. Russell, A new species of *Globidens* from South Dakota, and a review of the globidentine mosasaurs. *Fieldiana Geology* **33**, 235-256 (1975).

57. H. G. Seeley, On Remains of a small Lizard from the Neocomian Rocks of Comén, near Trieste preserved in the Geological Museum of the University of Vienna. *Quarterly Journal of the Geological Society* **37**, 52-56 (1881).

58. T. R. Simões, E. Wilner, M. W. Caldwell, L. C. Weinschütz, A. W. Kellner, A stem acrodontan lizard in the Cretaceous of Brazil revises early lizard evolution in Gondwana. *Nature Communications* **6**, 1-8 (2015).

59. K. T. Smith, M.-C. Buchy, A new aigialosaur (Squamata: Anguimorpha) with soft tissue remains from the Upper Cretaceous of Nuevo León, Mexico. *Journal of Vertebrate Paleontology* **28**, 85-94 (2008).

60. E. Tchernov, O. Rieppel, H. Zaher, M. J. Polcyn, L. L. Jacobs, A fossil snake with limbs. *Science* **287**, 2010-2012 (2000).

61. R. Vullo, A new species of *Lapparentophis* from the mid-Cretaceous Kem Kem beds, Morocco, with remarks on the distribution of lapparentophiid snakes. *Comptes Rendus Palevol* **18**, 765-770 (2019).

62. R. Vullo, J.-C. Rage, The first Gondwanan borioteiioid lizard and the mid-Cretaceous dispersal event between North America and Africa. *The Science of Nature* **105**, 1-8 (2018).

63. K. R. Wright, S. W. Shannon, *Selmasaurus rosselli*, a new plioplatecarpine mosasaur (Squamata, Mosasauridae) from Alabama. *Journal of Vertebrate Paleontology* **8**, 102-107 (1988).

64. L. Xing *et al.*, A mid-Cretaceous embryonic-to-neonate snake in amber from Myanmar. *Science Advances* **4**, eaat5042 (2018).

***Campanian Squamate References***

1. A. Albino (1986) Nuevos Boidae Madtsoiinae en el Cretácico tardío de Patagonia (Formación Los Alamitos, Río Negro, Argentina). in *Actas IV Congreso Argentino de Paleontología y Bioestratigrafía* (Mendoza), pp 15-21.

2. A. M. Albino, Una nueva serpiente (Reptilia) en el Cretácico superior de Patagonia, Argentina. *Pesquisas* **21**, 58-63 (1994).

3. V. Alifanov, New lizards of the family Macrocephalosauridae (Sauria) from the Upper Cretaceous of Mongolia, critical remarks on the systematics of the Teiidae (sensu Estes, 1983). *Paleontological Journal* **27**, 70-90 (1993).

4. V. Alifanov, *Desertiguana gobiensis* gen. et sp. nov., a new lizard (Phrynosomatidae, Iguanomorpha) from the Upper Cretaceous of Mongolia. *Paleontological Journal* **47**, 417-424 (2013).

5. M. Borsuk-Bialynicka, Anguimorphans and related lizards from the Late Cretaceous of the Gobi Desert, Mongolia. *Palaeontologia Polonica* **46**, 5-105 (1984).

6. M. Borsuk-Bialynicka, *Globaura venusta* gen et sp. n and *Eoxanta lacertifrons* gen. et sp. n.-non-teiid lacertoids from the Late Cretaceous of Mongolia. *Acta Palaeontologica Polonica* **33** (1988).

7. M. Borsuk-Bialynicka, *Gobekko cretacicus* gen. et sp. n., a new gekkonid lizard from the Cretaceous of the Gobi Desert. *Acta Palaeontologica Polonica* **35** (1990).

8. M. Borsuk-Bialynicka, V. R. Alifanov, First Asiatic ‘iguanid’ lizards in the Late Cretaceous of Mongolia. *Acta Palaeontologica Polonica* **36**, 325-342 (1991).

9. M. Borsuk-Bialynicka, S. M. Moody, Priscagaminae, a new subfamily of the Agamidae (Sauria) from the Late Cretaceous of the Gobi Desert. *Acta Palaeontologica Polonica* **29** (1984).

10. T. S. Bullard, M. W. Caldwell, Redescription and rediagnosis of the tylosaurine mosasaur *Hainosaurus pembinensis* Nicholls, 1988, as *Tylosaurus pembinensis* (Nicholls, 1988). *Journal of Vertebrate Paleontology* **30**, 416-426 (2010).

11. M. W. Caldwell, R. Holmes, G. L. Bell Jr, J. Wiffen, An unusual tylosaurine mosasaur from New Zealand: a new skull of *Taniwhasaurus oweni* (Lower Haumurian; Upper Cretaceous). *Journal of Vertebrate Paleontology* **25**, 393-401 (2005).

12. P. Christiansen, N. Bonde, A new species of gigantic mosasaur from the Late Cretaceous of Israel. *Journal of Vertebrate Paleontology* **22**, 629-644 (2002).

13. J. L. Conrad, M. A. Norell, A complete Late Cretaceous iguanian (Squamata, Reptilia) from the Gobi and identification of a new iguanian clade. *American Museum Novitates* **2007**, 1-47 (2007).

14. R. S. Cuthbertson, R. B. Holmes, A new species of *Plioplatecarpus* (Mosasauridae, Plioplatecarpinae) from the Bearpaw Formation (Campanian, Upper Cretaceous) of Montana, USA. *Journal of Vertebrate Paleontology* **35**, e922980 (2015).

15. R. S. Cuthbertson, J. C. Mallon, N. E. Campione, R. B. Holmes, A new species of mosasaur (Squamata: Mosasauridae) from the Pierre Shale (lower Campanian) of Manitoba. *Canadian Journal of Earth Sciences* **44**, 593-606 (2007).

16. D. G. DeMar Jr, J. L. Conrad, J. J. Head, D. J. Varricchio, G. P. Wilson, A new Late Cretaceous iguanomorph from North America and the origin of New World Pleurodonta (Squamata, Iguania). *Proceedings of the Royal Society B: Biological Sciences* **284**, 20161902 (2017).

17. R. K. Denton Jr, R. C. O'Neill, *Prototeius stageri*, gen. et sp. nov., a new teiid lizard from the Upper Cretaceous Marshalltown Formation of New Jersey, with a preliminary phylogenetic revision of the Teiidae. *Journal of Vertebrate Paleontology* **15**, 235-253 (1995).

18. M. Everhart, *Tylosaurus kansasensis*, a new species of tylosaurine (Squamata, Mosasauridae) from the Niobrara Chalk of western Kansas, USA. *Netherlands Journal of Geosciences* **84**, 231-240 (2005).

19. K. Gao, R. Fox, New teiid lizards from the Upper Cretaceous Oldman Formation (Judithian) of southeastern Alberta, Canada, with a review of the Cretaceous record of teiids. *Annals of the Carnegie Museum* **60**, 145-162 (1991).

20. K. Gao, R. C. Fox, Taxonomy and evolution of Late Cretaceous lizards (Reptilia: Squamata) from western Canada. *Bulletin of the Carnegie Museum of Natural History* **33**, 1-107 (1996).

21. K. Gao, L. Hou, Iguanians from the Upper Cretaceous Djadochta Formation, Gobi Desert, China. *Journal of Vertebrate Paleontology* **15**, 57-78 (1995).

22. K. Gao, L. Hou, Systematics and taxonomic diversity of squamates from the Upper Cretaceous Djadochta Formation, Bayan Mandahu, Gobi Desert, People's Republic of China. *Canadian Journal of Earth Sciences* **33**, 578-598 (1996).

23. K. Gao, M. Norell, M. S. U. Akademi, M.-A. M. P. Project, Taxonomic revision of *Carusia* (Reptilia, Squamata) from the late Cretaceous of the Gobi Desert and phylogenetic relationships of anguimorphan lizards. American Museum novitates; no. 3230. (1998).

24. K. Gao, M. A. Norell, Taxonomic composition and systematics of Late Cretaceous lizard assemblages from Ukhaa Tolgod and adjacent localities, Mongolian Gobi Desert. *Bulletin of the American Museum of Natural History* **2000**, 1-118 (2000).

25. A. Gaudry, *Les pythonomorphes de France* (Baudry, 1892).

26. C. W. Gilmore, A new mosasauroid reptile from the Cretaceous of Alabama. *Proceedings of the United States National Museum* **41**, 479-484 (1912).

27. C. W. Gilmore, Fossil lizards of Mongolia. *Bulletin of the American Museum of Natural History* **81**, 361-385 (1943).

28. R. O. Gómez, A. M. Báez, G. W. Rougier, An anilioid snake from the Upper Cretaceous of northern Patagonia. *Cretaceous Research* **29**, 481-488 (2008).

29. D. Grigoriev, Redescription of *Prognathodon lutugini* (Squamata, Mosasauridae). *Труды Зоологического института РАН* **317**, 246-261 (2013).

30. P. Jiménez-Huidobro, M. W. Caldwell, I. Paparella, T. S. Bullard, A new species of tylosaurine mosasaur from the upper Campanian Bearpaw Formation of Saskatchewan, Canada. *Journal of Systematic Palaeontology* **17**, 849-864 (2019).

31. M. Kearney, The phylogenetic position of *Sineoamphisbaena hexatabularis* reexamined. *Journal of Vertebrate Paleontology* **23**, 394-403 (2003).

32. T. Konishi, M. W. Caldwell, Two new plioplatecarpine (Squamata, Mosasauridae) genera from the Upper Cretaceous of North America, and a global phylogenetic analysis of plioplatecarpines. *Journal of Vertebrate Paleontology* **31**, 754-783 (2011).

33. T. Konishi, M. W. Caldwell, G. L. Bell Jr, Redescription of the holotype of *Platecarpus tympaniticus* Cope, 1869 (Mosasauridae: Plioplatecarpinae), and its implications for the alpha taxonomy of the genus. *Journal of Vertebrate Paleontology* **30**, 1410-1421 (2010).

34. T. Konishi, J. Lindgren, M. W. Caldwell, L. Chiappe, *Platecarpus tympaniticus* (Squamata, Mosasauridae): osteology of an exceptionally preserved specimen and its insights into the acquisition of a streamlined body shape in mosasaurs. *Journal of Vertebrate Paleontology* **32**, 1313-1327 (2012).

35. T. Konishi, M. G. Newbrey, M. W. Caldwell, A small, exquisitely preserved specimen of *Mosasaurus missouriensis* (Squamata, Mosasauridae) from the upper Campanian of the Bearpaw Formation, western Canada, and the first stomach contents for the genus. *Journal of Vertebrate Paleontology* **34**, 802-819 (2014).

36. M. C. Langer, Gilmoreteiidae new family and *Gilmoreteius* new genus (Squamata, Scincomorpha): replacement names for Macrocephalosauridae Sulimski, 1975 and *Macrocephalosaurus* Gilmore, 1943. *Comun. Mus. Ciênc. Tecnol. PUCRS, Sér Zool. Porto Alegre* **11**, 13-18 (1998).

37. J. Lindgren, M. Siverson, *Tylosaurus ivoensis*: a giant mosasaur from the early Campanian of Sweden. *Earth and Environmental Science Transactions of the Royal Society of Edinburgh* **93**, 73-93 (2002).

38. T. Lingham-Soliar, Mosasaurs from the upper Cretaceous of Niger. *Palaeontology* **34**, 653-670 (1991).

39. J. R. Lively, Redescription and phylogenetic assessment of *‘Prognathodon’ stadtmani*: implications for Globidensini monophyly and character homology in Mosasaurinae. *Journal of Vertebrate Paleontology* **40**, e1784183 (2020).

40. N. R. Longrich, A new species of *Pluridens* (Mosasauridae: Halisaurinae) from the upper Campanian of Southern Nigeria. *Cretaceous Research* **64**, 36-44 (2016).

41. J. E. Martin, "A North American *Hainosaurus* (Squamata: Mosasauridae) from the Late Cretaceous of southern South Dakota" in The Geology and Paleontology of the Late Cretaceous Marine Deposits of the Dakotas*,* J. E. Martin, Parris, David C., Ed. (Geological Society of America, 2007), vol. 427, pp. 0.

42. J. E. Martin, M. Fernández, The synonymy of the Late Cretaceous mosasaur (Squamata) genus *Lakumasaurus* from Antarctica with *Taniwhasaurus* from New Zealand and its bearing upon faunal similarity within the Weddellian Province. *Geological Journal* **42**, 203-211 (2007).

43. J. E. Martin, J. E. Martin, D. C. Parris, "A new species of the durophagous mosasaur *Globidens* (Squamata: Mosasauridae) from the Late Cretaceous Pierre Shale Group of central South Dakota, USA" in The Geology and Paleontology of the Late Cretaceous Marine Deposits of the Dakotas. (Geological Society of America, 2007), vol. 427, pp. 0.

44. W. R. Nava, A. G. Martinelli, A new squamate lizard from the Upper Cretaceous Adamantina Formation (Bauru Group), São Paulo State, Brazil. *Anais da Academia Brasileira de Ciências* **83**, 291-299 (2011).

45. M. Norell, K. Gao, M. S. U. Akademi, Braincase and phylogenetic relationships of *Estesia mongoliensis* from the late Cretaceous of the Gobi Desert and the recognition of a new clade of lizards. American Museum novitates; no. 3211. (1997).

46. M. A. Norell, K. Gao, J. Conrad, A new platynotan lizard (Diapsida: Squamata) from the Late Cretaceous Gobi Desert (Ömnögov), Mongolia. *American Museum Novitates* **2008**, 1-22 (2008).

47. M. A. Norell, M. J. MacKenna, M. J. Novacek, *Estesia mongoliensis, a new fossil varanoid from the Late Cretaceous Barun Goyot Formation of Mongolia* (American Museum of Natural History, 1992).

48. R. L. Nydam, "Polyglyphanodontinae (Squamata: Teiidae) from the Medial and Late Cretaceous: new taxa from Utah, USA and Baja California del Norte, Mexico" in Vertebrate Paleontology in Utah*,* D. D. Gillette, Ed. (Utah Geological Survey, Salt Lake City, 1999), vol. 99, pp. 303-317.

49. R. L. Nydam, "Lizards and Snakes from the Cenomanian through Campanian of Southern Utah: Filling the Gap in the Fossil Record of Squamata from the Late Cretaceous of the Western Interior of North America" in At the Top of the Grand Staircase: the Late Cretaceous of Southern Utah*,* A. Titus, M. Loewen, Eds. (Indiana University Press Bloomington, Indiana, 2013), pp. 370-423.

50. R. L. Nydam, M. W. Caldwell, F. Fanti, Borioteiioidean lizard skulls from Kleskun Hill (Wapiti Formation; upper Campanian), west-central Alberta, Canada. *Journal of Vertebrate Paleontology* **30**, 1090-1099 (2010).

51. R. L. Nydam, J. G. Eaton, J. Sankey, New taxa of transversely-toothed lizards (Squamata: Scincomorpha) and new information on the evolutionary history of “teiids”. *Journal of Paleontology* **81**, 538-549 (2007).

52. R. L. Nydam, B. M. Fitzpatrick, The occurrence of *Contogenys*-like lizards in the Late Cretaceous and Early Tertiary of the Western Interior of the USA. *Journal of Vertebrate Paleontology* **29**, 677-701 (2009).

53. R. L. Nydam, T. B. Rowe, R. L. Cifelli, Lizards and snakes of the Terlingua Local Fauna (late Campanian), Aguja Formation, Texas, with comments on the distribution of paracontemporaneous squamates throughout the Western Interior of North America. *Journal of Vertebrate Paleontology* **33**, 1081-1099 (2013).

54. R. L. Nydam, G. E. Voci, Teiid-like scincomorphan lizards from the Late Cretaceous (Campanian) of southern Utah. *Journal of Herpetology* **41**, 211-219 (2007).

55. I. Paparella, A. Palci, U. Nicosia, M. W. Caldwell, A new fossil marine lizard with soft tissues from the Late Cretaceous of southern Italy. *Royal Society open science* **5**, 172411 (2018).

56. M. a. E. d. Páramo-Fonseca, *Eonatator coellensis* nov. sp. (Squamata: Mosasauridae), nueva especie del Cretácico Superior de Colombia. *La Revista de la Academia Colombiana de Ciencias* **37**, 499-518 (2013).

57. J.-Y. Park, S. E. Evans, M. Huh, The first lizard fossil (Reptilia: Squamata) from the Mesozoic of South Korea. *Cretaceous Research* **55**, 292-302 (2015).

58. J.-C. Rage, Les Madtsoiidae (Reptilia, Serpentes) du Crétacé supérieur d’Europe: témoins gondwaniens d’une dispersion transtéthysienne. *Comptes-Rendus de l’Académie des Sciences de Paris* **322**, 603-608 (1996).

59. J. C. Rage, Werner, C., Mid-Cretaceous (Cenomanian) snakes from Wadi Abu Hashim, Sudan: the earliest snake assemblage. *Palaeontologia Africana* **35**, 85-110 (1999).

60. D. A. Russell, Systematics and morphology of American mosasaurs. *Yale University Peabody Museum of Natural History Bulletin* **23**, 1-241 (1967).

61. H. G. Seeley, The Reptile Fauna of the Gosau Formation preserved in the Geological Museum of the University of Vienna: With a Note on the Geological Horizon of the Fossils at Neue Welt, west of Wiener Neustadt, by Edw. Suess, Ph. D., FMGS, &c., Professor of Geology in the University of Vienna, &c. *Quarterly Journal of the Geological Society* **37**, 620-706 (1881).

62. A. Sulimski, *Adamisaurus magnidentatus* n. gen., n. sp.(Sauria) from the Upper Cretaceous of Mongolia. *Palaeontologia Polonica* **27**, 33-40 (1972).

63. A. Sulimski, Macrocephalosauridae and Polyglyphanodontidae (Sauria) from the Late Cretaceous of Mongolia. *Paleontologia Polonica* **33**, 25-102 (1975).

64. A. Sulimski, A new Cretaceous scincomorph lizard from Mongolia. *Paleontologia Polonica* **46**, 143-155 (1984).

65. S. P. Welles, D. R. Gregg, Late Cretaceous marine reptiles of New Zealand. *Records of the Canterbury Museum* **9**, 1-111 (1971).

66. J. Wiffen, *Moanasaurus*, a new genus of marine reptile (Family Mosasauridae) from the Upper Cretaceous of North Island, New Zealand. *New Zealand Journal of Geology and Geophysics* **23**, 507-528 (1980).

67. X.-c. Wu *et al.*, Oldest known amphisbaenian from the Upper Cretaceous of Chinese Inner Mongolia. *Nature* **366**, 57-59 (1993).

68. H.-Y. Yi, M. A. Norell, New materials of *Estesia mongoliensis* (Squamata: Anguimorpha) and the evolution of venom grooves in lizards. *American Museum Novitates* **2013**, 1-31 (2013).

***Maastrichtian Squamate References***

1. N. Bardet, The mosasaur collections of the Muséum National d’Histoire Naturelle of Paris. *Bulletin de la Société géologique de France* **183**, 35-53 (2012).

2. N. Bardet, X. Pereda Suberbiola, M. Iarochene, B. Bouya, M. Amaghzaz, A new species of *Halisaurus*. from the Late Cretaceous phosphates of Morocco, and the phylogenetical relationships of the Halisaurinae (Squamata: Mosasauridae). *Zoological Journal of the Linnean Society* **143**, 447-472 (2005).

3. N. Bardet, X. P. Suberbiola, M. Iarochène, M. Amalik, B. Bouya, Durophagous Mosasauridae (Squamata) from the Upper Cretaceous phosphates of Morocco, with description of a new species of Globidens. *Netherlands Journal of Geosciences* **84**, 167-175 (2005).

4. N. Bardet *et al.*, *Mosasaurus beaugei* Arambourg, 1952 (Squamata, Mosasauridae) from the late Cretaceous phosphates of Morocco. *Geobios* **37**, 315-324 (2004).

5. V. A. Codrea, M. Venczel, A. Solomon, A new family of teiioid lizards from the Upper Cretaceous of Romania with notes on the evolutionary history of early teiioids. *Zoological Journal of the Linnean Society* **181**, 385-399 (2017).

6. L. Dollo, Première note sur les mosasauriens de Mesvin. *Bulletin de la Société belge de Géologie, de Paléontologie et d'Hydrologie* **3**, 271-304 (1889).

7. R. W. Dortangs *et al.*, A large new mosasaur from the Upper Cretaceous of The Netherlands. *Netherlands Journal of Geosciences* **81**, 1-8 (2002).

8. R. Estes, Fossil vertebrates from the Lance Formation, Easten Wyoming. *University of California Publications in Geological Sciences* **49**, 1-187 (1964).

9. R. Estes, *Handbuch Der Paläoherpetologie: Sauria Terrestria, Amphisbaenia. Teil 10A. Part 10A* (Lubrecht & Cramer Limited, 1983).

10. R. Estes, L. I. Price, Iguanid lizard from the Upper Cretaceous of Brazil. *Science* **180**, 748-751 (1973).

11. A. Folie, V. Codrea, New lissamphibians and squamates from the Maastrichtian of Hateg Basin, Romania. *Acta Palaeontologica Polonica* **50** (2005).

12. M. A. Fontana (2014) A Redescription and Phylogenetic Analysis of the Cretaceous Fossil Lizard *Polyglyphanodon sternbergi* Gilmore, 1940. (The George Washington University).

13. K. Gao, R. C. Fox, Taxonomy and evolution of Late Cretaceous lizards (Reptilia: Squamata) from western Canada. *Bulletin of the Carnegie Museum of Natural History* **33**, 1-107 (1996).

14. C. W. Gilmore, *Fossil lizards of North America* (U.S. G.P.O., Washington, 1928).

15. C. W. Gilmore, New fossil lizards from the Upper Cretaceous of Utah. *Smithsonian Miscellaneous Collections* (1940).

16. C. W. Gilmore, Osteology of *Polyglyphanodon*, an Upper Cretaceous lizard from Utah. *Proceedings of the United States National Museum* (1942).

17. C. W. Gilmore, Osteology of Upper Cretaceous lizards from Utah, with a description of a new species. *Proceedings of the United States National Museum* (1943).

18. R. O. Gómez, F. F. Garberoglio, G. W. Rougier, A new Late Cretaceous snake from Patagonia: Phylogeny and trends in body size evolution of madtsoiid snakes. *Comptes Rendus Palevol* **18**, 771-781 (2019).

19. A. Houssaye *et al.*, A review of *Pachyvaranus crassispondylus* Arambourg, 1952, a pachyostotic marine squamate from the latest Cretaceous phosphates of Morocco and Syria. *Geological Magazine* **148**, 237-249 (2011).

20. P. Jiménez-Huidobro, M. W. Caldwell, Reassessment and reassignment of the early Maastrichtian mosasaur *Hainosaurus bernardi* Dollo, 1885, to *Tylosaurus* Marsh, 1872. *Journal of Vertebrate Paleontology* **36**, e1096275 (2016).

21. T. Konishi, M. W. Caldwell, T. Nishimura, K. Sakurai, K. Tanoue, A new halisaurine mosasaur (Squamata: Halisaurinae) from Japan: the first record in the western Pacific realm and the first documented insights into binocular vision in mosasaurs. *Journal of Systematic Palaeontology* **14**, 809-839 (2016).

22. D. W. Krause, S. E. Evans, K.-Q. Gao, First definitive record of Mesozoic lizards from Madagascar. *Journal of Vertebrate Paleontology* **23**, 842-856 (2003).

23. T. C. Laduke, D. W. Krause, J. D. Scanlon, N. J. Kley, A late cretaceous (Maastrichtian) snake assemblage from the Maevarano formation, Mahajanga basin, Madagascar. *Journal of Vertebrate Paleontology* **30**, 109-138 (2010).

24. A. R. LeBlanc, M. W. Caldwell, N. Bardet, A new mosasaurine from the Maastrichtian (Upper Cretaceous) phosphates of Morocco and its implications for mosasaurine systematics. *Journal of Vertebrate Paleontology* **32**, 82-104 (2012).

25. J. Lindgren, Cranial osteology of the giant mosasaur *Plesiotylosaurus* (Squamata, Mosasauridae). *Journal of Paleontology* **83**, 448-456 (2009).

26. J. Lindgren, M. W. Caldwell, J. W. Jagt, New data on the postcranial anatomy of the California mosasaur *Plotosaurus bennisoni* (Camp, 1942)(Upper Cretaceous: Maastrichtian), and the taxonomic status of *P. tuckeri* (Camp, 1942). *Journal of Vertebrate Paleontology* **28**, 1043-1054 (2008).

27. T. Lingham-Soliar, The first description of *Halisaurus* (Reptilia Mosasauridae) from Europe, from the Upper Cretaceous of Belgium. *Bulletin de l'Institut Royal des Sciences Naturelles de Belgique, Sciences de la Terre* **66**, 129-136 (1996).

28. T. Lingham-Soliar, A new mosasaur *Pluridens walkeri* from the Upper Cretaceous, Maastrichtian of the Iullemmeden Basin, southwest Niger. *Journal of Vertebrate Paleontology* **18**, 709-717 (1998).

29. N. R. Longrich, B.-A. S. Bhullar, J. A. Gauthier, Mass extinction of lizards and snakes at the Cretaceous–Paleogene boundary. *Proceedings of the National Academy of Sciences* **109**, 21396-21401 (2012).

30. N. R. Longrich, B.-A. S. Bhullar, J. A. Gauthier, A transitional snake from the Late Cretaceous period of North America. *Nature* **488**, 205-208 (2012).

31. J.-c. Lü, S.-a. Ji, Z.-m. Dong, X.-c. Wu, An Upper Cretaceous lizard with a lower temporal arcade. *Naturwissenschaften* **95**, 663-669 (2008).

32. O. C. Marsh, Notice of new reptiles from the Laramie Formation. *American Journal of Science* **3**, 449-453 (1892).

33. J.-y. Mo, X. Xu, S. E. Evans, A large predatory lizard (Platynota, Squamata) from the Late Cretaceous of South China. *Journal of Systematic Palaeontology* **10**, 333-339 (2012).

34. D. M. Mohabey, J. J. Head, J. A. Wilson, A new species of the snake Madtsoia from the Upper Cretaceous of India and its paleobiogeographic implications. *Journal of Vertebrate Paleontology* **31**, 588-595 (2011).

35. R. L. Nydam, M. W. Caldwell, F. Fanti, Borioteiioidean lizard skulls from Kleskun Hill (Wapiti Formation; upper Campanian), west-central Alberta, Canada. *Journal of Vertebrate Paleontology* **30**, 1090-1099 (2010).

36. R. L. Nydam, R. L. Cifelli, New data on the dentition of the scincomorphan lizard *Polyglyphanodon sternbergi*. *Acta Palaeontologica Polonica* **50** (2005).

37. R. L. Nydam, B. M. Fitzpatrick, The occurrence of *Contogenys*-like lizards in the Late Cretaceous and Early Tertiary of the Western Interior of the USA. *Journal of Vertebrate Paleontology* **29**, 677-701 (2009).

38. R. L. Nydam, J. A. Gauthier, J. J. Chiment, The mammal-like teeth of the Late Cretaceous lizard *Peneteius aquilonius* Estes 1969 (Squamata, Teiidae). *Journal of Vertebrate Paleontology* **20**, 628-631 (2000).

39. R. L. Nydam, G. E. Voci, Teiid-like scincomorphan lizards from the Late Cretaceous (Campanian) of southern Utah. *Journal of Herpetology* **41**, 211-219 (2007).

40. R. A. Otero, S. Soto-Acuna, D. Rubilar-Rogers, C. S. Gutstein, *Kaikaifilu hervei* gen. et sp. nov., a new large mosasaur (Squamata, Mosasauridae) from the upper Maastrichtian of Antarctica. *Cretaceous Research* **70**, 209-225 (2017).

41. A. C. Pritchard, J. A. McCartney, D. W. Krause, N. J. Kley, New snakes from the Upper Cretaceous (Maastrichtian) Maevarano Formation, Mahajanga Basin, Madagascar. *Journal of Vertebrate Paleontology* **34**, 1080-1093 (2014).

42. J.-C. Rage, G. V. PRASAD, New snakes from the late Cretaceous (Maastrichtian) of Naskal, India. *Neues Jahrbuch für Geologie und Paläontologie. Abhandlungen* **187**, 83-97 (1992).

43. D. A. Russell, Systematics and morphology of American mosasaurs. *Yale University Peabody Museum of Natural History Bulletin* **23**, 1-241 (1967).

44. A. Schulp, N. Bardet, B. Bouya, A new species of the durophagous mosasaur *Carinodens* (Squamata, Mosasauridae) and additional material of *Carinodens belgicus* from the Maastrichtian phosphates of Morocco. *Netherlands Journal of Geosciences* **88**, 161-167 (2009).

45. A. S. Schulp, Feeding the mechanical mosasaur: what did *Carinodens* eat? *Netherlands Journal of Geosciences* **84**, 345-357 (2005).

46. A. S. Schulp, M. J. Polcyn, O. Mateus, L. L. Jacobs, M. L. Morais (2008) A new species of *Prognathodon* (Squamata, Mosasauridae) from the Maastrichtian of Angola, and the affinities of the mosasaur genus *Liodon*. in *Proceedings of the Second Mosasaur Meeting* (Fort Hays State University Fort Hays), pp 1-12.

47. T. Soliar, The mosasaur *Goronyosaurus* from the upper cretaceous of Sokoto state, Nigeria. *Palaeontology* **31**, 747-762 (1988).

48. H. P. Street, M. W. Caldwell, Rediagnosis and redescription of *Mosasaurus hoffmannii* (Squamata: Mosasauridae) and an assessment of species assigned to the genus Mosasaurus. *Geological Magazine* **154**, 521-557 (2017).

49. Ş. Vasile, Z. Csiki-Sava, M. Venczel, A new madtsoiid snake from the Upper Cretaceous of the Haţeg Basin, western Romania. *Journal of Vertebrate Paleontology* **33**, 1100-1119 (2013).

50. M. Venczel, V. A. Codrea, A new teiid lizard from the Late Cretaceous of the Haţeg Basin, Romania and its phylogenetic and palaeobiogeographical relationships. *Journal of Systematic Palaeontology* **14**, 219-237 (2016).

51. J. A. Wilson, D. M. Mohabey, S. E. Peters, J. J. Head, Predation upon hatchling dinosaurs by a new snake from the Late Cretaceous of India. *PLoS biology* **8**, e1000322 (2010).

52. L. Xu *et al.*, A New Lizard (Lepidosauria: Squamata) from the Upper Cretaceous of Henan, China. *Acta Geologica Sinica‐English Edition* **88**, 1041-1050 (2014).

***Paleocene Squamate References***

1. A. M. Albino, S. Brizuela, An overview of the South American fossil squamates. *The Anatomical Record* **297**, 349-368 (2014).

2. V. Alifanov, A New Platynotan Lizard (Parasaniwidae, Anguimorpha) from the Late Paleocene of Southern Mongolia. *Paleontological Journal* **52**, 1432-1435 (2018).

3. M. Augé, J.-C. Rage (2006) Herpetofaunas from the upper Paleocene and lower Eocene of Morocco. in *Annales de Paléontologie* (Elsevier), pp 235-253.

4. M. L. Augé, Évolution des lézards du Paléogène en Europe. *Mémoires du Muséum national d'histoire naturelle* **192**, 3-369 (2005).

5. A. O. Averianov, Paleogene sea snakes from the eastern part of Tethys. *Russian Journal of Herpetology* **4**, 128-142 (1997).

6. E. D. Cope, On some Cretaceous reptilia. *Proceedings of the Academy of Natural Sciences of Philadelphia*, 233-242 (1868).

7. E. D. Cope, First addition to the fauna of the Puerco Eocene. *Proceedings of the American Philosophical Society* **20**, 545-563 (1883).

8. L. Dong, S. E. Evans, Y. Wang, Taxonomic revision of lizards from the Paleocene deposits of the Qianshan Basin, Anhui, China. *Vertebrata PalAsiatica* **54**, 243-268 (2016).

9. A. Folie, B. Sigé, T. Smith, A new scincomorph lizard from the Palaeocene of Belgium and the origin of Scincoidea in Europe. *Naturwissenschaften* **92**, 542-546 (2005).

10. A. Folie, R. Smith, T. Smith, New amphisbaenian lizards from the Early Paleogene of Europe and their implications for the early evolution of modern amphisbaenians. *Geologica Belgica* (2013).

11. J. A. Gauthier, Fossil xenosaurid and anguid lizards from the early Eocene Wasatch Formation, southeast Wyoming, and a revision of the Anguioidea. *Rocky Mountain Geology* **21**, 7-54 (1982).

12. C. W. Gilmore, *Fossil lizards of North America* (U.S. G.P.O., Washington, 1928).

13. C. W. Gilmore, Fossil Snakes of North America. *Geological Society of America, Special Papers* **9**, 1-96 (1938).

14. C. W. Gilmore, Paleocene faunas of the Polecat Bench Formation, Park County, Wyoming Part II. Lizards. *Proceedings of the American Philosophical Society*, 159-167 (1942).

15. J. J. Head *et al.*, Giant boid snake from the Palaeocene neotropics reveals hotter past equatorial temperatures. *Nature* **457**, 715-717 (2009).

16. N. R. Longrich, J. Vinther, R. A. Pyron, D. Pisani, J. A. Gauthier, Biogeography of worm lizards (Amphisbaenia) driven by end-Cretaceous mass extinction. *Proceedings of the Royal Society B: Biological Sciences* **282**, 20143034 (2015).

17. J. A. McCartney, E. M. Roberts, L. Tapanila, M. A. O'Leary, Large palaeophiid and nigerophiid snakes from Paleogene Trans-Saharan Seaway deposits of Mali. *Acta Palaeontologica Polonica* **63**, 207-220 (2018).

18. R. L. Nydam, B. M. Fitzpatrick, The occurrence of *Contogenys*-like lizards in the Late Cretaceous and Early Tertiary of the Western Interior of the USA. *Journal of Vertebrate Paleontology* **29**, 677-701 (2009).

19. D. Parmley, G. R. Case, Palaeopheid snakes from the Gulf Coastal region of North America. *Journal of Vertebrate Paleontology* **8**, 334-339 (1988).

20. J. C. Rage, Fossil snakes from the Palaeocene of São José de Itaboraí, Brazil. Part I. Madtsoiidae, Aniliidae. *Palaeovertebrata* **27**, 109-144 (1998).

21. J. C. Rage, Fossil snakes from the Palaeocene of São José de Itaboraí, Brazil. Part II. Boidae. *Palaeovertebrata* **30**, 111-150 (2001).

22. J. C. Rage, Fossil snakes from the Palaeocene of São José de Itaboraí, Brazil. Part III. Ungaliophiinae, booids incertae sedis, and Caenophidia. Summary, update and discussion of the snake fauna from the locality. *Palaeovertebrata* **36**, 37-73 (2008).

23. A. Scanferla, H. Zaher, F. E. Novas, C. de Muizon, R. Céspedes, A new snake skull from the Paleocene of Bolivia sheds light on the evolution of macrostomatans. *PLoS One* **8**, e57583 (2013).

24. R. M. Sullivan, Fossil lizards from Swain Quarry" Fort Union Formation," middle Paleocene (Torrejonian), Carbon County, Wyoming. *Journal of Paleontology*, 996-1010 (1982).

25. R. M. Sullivan, A new middle Paleocene (Torrejonian) rhineurid amphisbaenian, *Plesiorhineura tsentasi* new genus, new species, from the San Juan Basin, New Mexico. *Journal of Paleontology*, 1481-1485 (1985).

26. R. M. Sullivan, A new fossil anguid lizard *Parodaxosaurus sanjuanensis*, new genus, new species from the Middle Paleocene (Torrejonian) Nacimiento Formation, San Juan Basin, New Mexico. *Journal of herpetology* **20**, 109-111 (1986).

27. R. M. Sullivan, Paleocene Caudata and Squamata from Gidley and Silberling quarries, Montana. *Journal of Vertebrate Paleontology* **11**, 293-301 (1991).

28. R. M. Sullivan, S. G. Lucas, *Palaeoscincosaurus middletoni*, new genus and species (Squamata:? Scincidae) from the early Paleocene (Puercan) Denver Formation, Colorado. *Journal of Vertebrate Paleontology* **16**, 666-672 (1996).

***Eocene Squamate References***

1. A. M. Albino, Snakes from the Paleocene and Eocene of Patagonia (Argentina): paleoecology and coevolution with mammals. *Historical Biology* **7**, 51-69 (1993).

2. A. M. Albino, New macrostomatan snake from the Paleogene of northwestern Argentina. *Geobios* **51**, 175-179 (2018).

3. V. Alifanov, New acrodont lizards (Lacertilia) from the Middle Eocene of southern Mongolia. *Paleontological Journal* **43**, 675-685 (2009).

4. V. Alifanov, Lizards of the family Arretosauridae Gilmore, 1943 (Iguanomorpha, Iguania) from the Paleogene of Mongolia. *Paleontological Journal* **46**, 412-420 (2012).

5. W. Auffenberg, *Anomalophis bolcensis (Massalongo), a new genus of fossil snake from the Italian Eocene*, Breviora (Museum of Comparative Zoology, Cambridge, 1959), vol. 114.

6. M. Augé, La faune de Lacertilia (Reptilia, Squamata) de l’Éocène inférieur de Prémontré (Bassin de Paris, France). *Geodiversitas* **25** (2003).

7. M. Augé, Past and present distribution of iguanid lizards. *Arquivos do Museu Nacional, Rio de Janeiro* **65**, 403-416 (2007).

8. M. Augé, R. Smith, Les Agamidae (Reptilia, Squamata) du Paleogene d'Europe occidentale. *Belgian Journal of Zoology* **127**, 123-138 (1997).

9. M. Augé, R. Smith, Nouveaux Lacertidae (Reptilia, Squamata) de l'Eocène inférieur européen. *Belgian Journal of Zoology* **132**, 3-16 (2002).

10. M. Augé, R. M. Sullivan, A new genus, *Paraplacosauriops* (Squamata, Anguidae, Glyptosaurinae), from the Eocene of France. *Journal of Vertebrate Paleontology* **26**, 133-137 (2006).

11. M. L. Augé, Évolution des lézards du Paléogène en Europe. *Mémoires du Muséum national d'histoire naturelle* **192**, 3-369 (2005).

12. M. L. Augé, Amphisbaenians from the European Eocene: a biogeographical review. *Palaeobiodiversity and Palaeoenvironments* **92**, 425-443 (2012).

13. A. O. Averianov, Paleogene sea snakes from the eastern part of Tethys. *Russian Journal of Herpetology* **4**, 128-142 (1997).

14. S. Bajpai, J. J. Head, An early Eocene palaeopheid snake from Vastan lignite mine, Gujarat, India. *Gondwana Geological Magazine* **22**, 85-90 (2007).

15. S. Baszio, *Messelophis variatus* n. gen. n. sp. from the Eocene of Messel: a tropidopheine snake with affinities to Erycinae (Boidae). *Courier Forschungsinstitut Senckenberg* **252**, 47-66 (2004).

16. A. M. Bauer, W. Böhme, W. Weitschat, An Early Eocene gecko from Baltic amber and its implications for the evolution of gecko adhesion. *Journal of Zoology* **265**, 327-332 (2005).

17. D. S. Berman, *Hyporhina tertia*, new species (Reptilia: Amphisbaenia), from the Early Oligocene (Chadronian) White River Formation of Wyoming. *Annals of the Carnegie Museum* **44**, 1-10 (1972).

18. D. S. Berman, *Spathorhynchus fossorium*, a middle Eocene amphisbaenian (Reptilia) from Wyoming. *Copeia*, 704-721 (1973).

19. D. S. Berman, *Spathorhynchus natronicus*, a new species of rhineurid amphisbaenian (Reptilia) from the early Oligocene of Wyoming. *Journal of Paleontology* **51**, 986-991 (1977).

20. A. Bolet, M. Augé, A new miniaturized lizard from the late Eocene of France and Spain. *The Anatomical Record* **297**, 505-515 (2014).

21. A. Bolet, J. D. Daza, New genus and species names for the Eocene lizard *Cadurcogekko* *rugosus* Augé, 2005. *Zootaxa* **3985**, 265-274 (2015).

22. B. H. Brattstrom, New snakes and lizards from the Eocene of California. *Journal of Paleontology* **29**, 145-149 (1955).

23. A. Čerňanský, M. L. Augé, J.-C. Rage, A complete mandible of a new amphisbaenian reptile (Squamata, Amphisbaenia) from the late Middle Eocene (Bartonian, MP 16) of France. *Journal of Vertebrate Paleontology* **35**, e902379 (2015).

24. J. L. Conrad, An Eocene shinisaurid (Reptilia, Squamata) from Wyoming, USA. *Journal of Vertebrate Paleontology* **26**, 113-126 (2006).

25. J. L. Conrad, A new Eocene casquehead lizard (Reptilia, Corytophanidae) from North America. *PLoS One* **10**, e0127900 (2015).

26. J. L. Conrad, O. Rieppel, L. Grande, A Green River (Eocene) polychrotid (Squamata: Reptilia) and a re-examination of iguanian systematics. *Journal of Paleontology* **81**, 1365-1373 (2007).

27. E. D. Cope, On some Cretaceous reptilia. *Proceedings of the Academy of Natural Sciences of Philadelphia*, 233-242 (1868).

28. E. D. Cope, Third account of new Vertebrata from the Bridger Eocene of Wyoming Territory. *Proceedings of the American Philosophical Society* **12**, 469-472 (1871).

29. I. G. Danilov, A. O. Averianov, A new species of *Calamagras* Cope, 1873 (Serpentes, Boidae, Erycinae) from the early Eocene of Kirghizia. *Geodiversitas* **21**, 85-91 (1999).

30. A. T. de Rochebrune, *Révision des ophidiens fossiles du Muséum d'Histoire naturelle*, Nouvelles Archives du Muséum, Pt. III (G. Masson, Paris, 1880).

31. L. Dollo, *Saniwa orsmaelensis*. Varanide nouveau du Landénien supérieur d'Orsmael (Brabant). *Bulletin de la Société Belge de Géologie, Paléontologie, et Hydrologie* **33**, 76-82 (1923).

32. O. E. Donadio, Un nuevo lacertilio (Squamata, Sauria, Teiidae) de la formación Lumbrera (eoceno temprano) provincia de Salta, Argentina. *Ameghiniana* **22**, 221-228 (1985).

33. R. Estes, *Handbuch Der Paläoherpetologie: Sauria Terrestria, Amphisbaenia. Teil 10A. Part 10A* (Lubrecht & Cramer Limited, 1983).

34. H. Filhol, Recherches sur les Phosphorites du Quercy. Étude des fossiles qu’on y rencontre et spécialement des mammifères. Pt. II. *Annales des Sciences Géologiques* **8**, 1-340 (1877).

35. A. Folie, R. Smith, T. Smith, New amphisbaenian lizards from the Early Paleogene of Europe and their implications for the early evolution of modern amphisbaenians. *Geologica Belgica* (2013).

36. C. Fries, C. W. Hibbard, D. H. Dunkle, Early Cenozoic vertebrates in the red conglomerate at Guanajuato, Mexico. *Smithsonian Miscellaneous Collections* (1955).

37. J. A. Gauthier, Fossil xenosaurid and anguid lizards from the early Eocene Wasatch Formation, southeast Wyoming, and a revision of the Anguioidea. *Rocky Mountain Geology* **21**, 7-54 (1982).

38. G. L. Georgalis, T. M. Scheyer, A new species of *Palaeopython* (Serpentes) and other extinct squamates from the Eocene of Dielsdorf (Zurich, Switzerland). *Swiss Journal of Geosciences* **112**, 383-417 (2019).

39. P. Gervais, *Zoologie et paléontologie françaises (animaux vertébrés) : ou nouvelles recherches sur les animaux vivants et fossiles de la France* (Arthus Bertrand, Paris, 1848).

40. C. W. Gilmore, Fossil Snakes of North America. *Geological Society of America, Special Papers* **9**, 1-96 (1938).

41. C. W. Gilmore, Paleocene faunas of the Polecat Bench Formation, Park County, Wyoming Part II. Lizards. *Proceedings of the American Philosophical Society*, 159-167 (1942).

42. C. W. Gilmore, Fossil lizards of Mongolia. *Bulletin of the American Museum of Natural History* **81**, 361-385 (1943).

43. C. W. Gilmore, G. L. Jepsen, A new Eocene lizard from Wyoming. *Journal of Paleontology* **19**, 30-34 (1945).

44. J. J. Head, A South American snake lineage from the Eocene Greenhouse of North America and a reappraisal of the fossil record of “anilioid” snakes. *Geobios* **66**, 55-65 (2021).

45. J. J. Head, G. F. Gunnell, P. A. Holroyd, J. H. Hutchison, R. L. Ciochon, Giant lizards occupied herbivorous mammalian ecospace during the Paleogene greenhouse in Southeast Asia. *Proceedings of the Royal Society B: Biological Sciences* **280**, 20130665 (2013).

46. M. K. Hecht, P. O. McGrew, G. G. Simpson, A new xantusiid lizard from the Eocene of Wyoming. *American Museum Novitates*, 1-8 (1956).

47. R. Hoffstetter, Un serpent marin du genre *Pterosphenus* (*P. sheppardi* nov. sp.) dans l'Eocene superieur de l'Equateur (Amerique du Sud). *Bulletin de la Société geologique de France* **6**, 45-50 (1958).

48. R. Hoffstetter, J.-C. Rage, Les Erycinae fossiles de France (Serpentes, Boidea) compréhension et histoire de la sous-famille. *Annales de Paléontologie (Vertébrés)* **58**, 81-124 (1972).

49. A. Holman, A new genus of diminutive boid snake from the Upper Eocene of Hordle Cliff, Hampshire, England. *Tertiary Research* **17**, 11-14 (1996).

50. J. A. Holman, Herpetofauna of the Calf Creek local fauna (lower Oligocene: Cypress Hills formation) of Saskatchewan. *Canadian Journal of Earth Sciences* **9**, 1612-1631 (1972).

51. J. A. Holman, Upper Eocene snakes (Reptilia, Serpentes) from Georgia. *Journal of Herpetology* **11**, 141-145 (1977).

52. J. A. Holman, *Palaeophis casei*, new species, a tiny palaeophid snake from the early Eocene of Mississippi. *Journal of Vertebrate Paleontology* **2**, 163-166 (1982).

53. J. A. Holman, A new genus of primitive colubroid snake from the upper Eocene, Isle of Wight, England. *Tertiary Research* **14**, 151-154 (1993).

54. J. A. Holman, G. R. Case, Reptiles from the Eocene Tallahatta Formation of Alabama. *Journal of Vertebrate Paleontology* **8**, 328-333 (1988).

55. J. A. Holman, D. L. Harrison, A new genus of small boid snake from Upper Eocene of Hordle Cliff, Hampshire, England. *Acta Zoologica Cracoviensia* **41** (1998).

56. J. A. Holman, D. L Harrison, D. J Ward, Late Eocene snakes from the Headon Hill Formation, southern England. *Cainozoic Research* **5**, 51-62 (2005).

57. A. Houssaye *et al.*, New highlights about the enigmatic marine snake *Palaeophis* *maghrebianus* (Palaeophiidae; Palaeophiinae) from the Ypresian (Lower Eocene) phosphates of Morocco. *Palaeontology* **56**, 647-661 (2013).

58. G. Keqin, D. Dashzeveg, New lizards from the Middle Eocene Mergen Formation, Mongolian Gobi Desert. *Paläontologische Zeitschrift* **73**, 327-335 (1999).

59. J. Klembara, B. Green, Anguimorph lizards (Squamata, Anguimorpha) from the middle and late Eocene of the Hampshire basin of southern England. *Journal of Systematic Palaeontology* **8**, 97-129 (2010).

60. O. Kühn, Die Placosauriden und Angiuden aus dem mittleren Eozän des Geiseltales. *Nova Acta Ieopoldina* **8**, 461-486 (1940).

61. O. Kuhn, Weitere Lacertilier, insbesondere Iguanidae aus dem Eozän des Geiseltales. *Paläontologische Zeitschrift* **23**, 360-367 (1944).

62. J. Leidy, *Contributions to the extinct vertebrate fauna of the western territories* (US Government Printing Office, 1873), vol. 1.

63. J. Li, Fossil reptiles from Hetaoyuan Formation, Xichuan, Henan. *Vertebrata Palasiatica* **29**, 190-203 (1991).

64. J. Li, Fossil reptiles from Zhaili Member, Hedi Formation, Yuanqu, Shanxi. *Vertebrata PalAsiatica* **29**, 276-285 (1991).

65. F. A. Lucas, A new snake from the Eocene of Alabama. *Proceedings of the United States National Museum* **21**, 637-638 (1898).

66. O. C. Marsh, ART. XL.--Description of a new and gigantic fossil Serpent (*Dinophis* *grandis*), from the Tertiary of New Jersey. *American Journal of Science and Arts (1820-1879)* **48**, 397 (1869).

67. O. C. Marsh, Description of some new Fossil Serpents, from the Tertiary Deposits of Wyoming. *American Journal of Science and Arts* **1**, 322-329 (1871).

68. O. C. Marsh, Preliminary Description of New Tertiary Reptiles. *American Journal of Science and Arts (1820-1879)* **4**, 298 (1872).

69. J. A. McCartney, E. R. Seiffert, A late Eocene snake fauna from the Fayum Depression, Egypt. *Journal of Vertebrate Paleontology* **36**, e1029580 (2016).

70. P. O. McGrew *et al.*, The geology and paleontology of the Elk Mountain and Tabernacle Butte area, Wyoming. Bulletin of the AMNH; v. 117, article 3. *Bulletin of the American Museum of Natural History* **117**, 117-176 (1959).

71. C. A. Meszoely, North American Fossil Anguid Lizards. *Bulletin of the Museum of Comparative Zoology* **139**, 87-149 (1970).

72. C. A. Meszoely, R. Estes, H. Haubold, Eocene anguid lizards from Europe and a revision of the genus *Xestops*. *Herpetologica*, 156-166 (1978).

73. J. Müller, Osteology and relationships of *Eolacerta* *robusta*, a lizard from the Middle Eocene of Germany (Reptilia, Squamata). *Journal of Vertebrate Paleontology* **21**, 261-278 (2001).

74. J. Müller *et al.*, Eocene lizard from Germany reveals amphisbaenian origins. *Nature* **473**, 364-367 (2011).

75. R. Owen, *A History of British Fossil Reptiles* (Cassell limited, London, 1849), vol. 3.

76. D. Parmley, G. R. Case, Palaeopheid snakes from the Gulf Coastal region of North America. *Journal of Vertebrate Paleontology* **8**, 334-339 (1988).

77. D. Parmley, M. DeVore, Palaeopheid snakes from the Late Eocene Hardie Mine local fauna of central Georgia. *Southeastern Naturalist* **4**, 703-722 (2005).

78. G. V. Prasad, S. Bajpai, Agamid lizards from the early Eocene of western India: oldest Cenozoic lizards from South Asia. *Palaeontologia Electronica* **11**, 1-20 (2008).

79. J.-C. Rage, Les serpents du phosphorites de Quercy. *Palaeovertebrata* **6**, 274-303 (1974).

80. J.-C. Rage, Un Caenophidien primitif (Reptilia, Serpentes) dans l’Eocène inferieur. *Compte Rendu Sommaire des Séances de la Société Géologique de France* **2**, 46-47 (1975).

81. J.-C. Rage, Un serpent marin nouveau de l'Eocene de Belgique: Le probleme des Serpents marins du Paleogene. *Comptes Rendus Hebdomadaires des Séances de l’Académie des Sciences, Série D* **291**, 469-471 (1980).

82. J.-C. Rage, The oldest known colubrid snakes. The state of the art. *Acta Zoologica Cracoviensia* **31**, 11-27 (1988).

83. J.-C. Rage, The lower vertebrates from the Eocene and Oligocene of the Phosphorites du Quercy (France): an overview. *Strata* **13**, 161-173 (2006).

84. J.-C. Rage, M. Augé, Squamate reptiles from the middle Eocene of Lissieu (France). A landmark in the middle Eocene of Europe. *Geobios* **43**, 253-268 (2010).

85. J.-C. Rage, S. Bajpai, J. G. Thewissen, B. N. Tiwari, Early Eocene snakes from Kutch, Western India, with a review of the Palaeophiidae. *Geodiversitas* **25**, 695-716 (2003).

86. J.-C. Rage *et al.*, A diverse snake fauna from the early Eocene of Vastan Lignite Mine, Gujarat, India. *Acta Palaeontologica Polonica* **53**, 391-403 (2008).

87. J. C. Rage, Présence de *Dunnophis* (Reptilia, Serpentes) dans l'Eocène et l'Oligocène européen. *Compte Rendu sommaire des séances de la Société Géologique de France* **1973**, 76-78 (1973).

88. J. C. Rage, An erycine snake (Boidae) of the genus *Calamagras* from the French lower Eocene, with comments on the phylogeny of the Erycinae. *Herpetologica*, 459-463 (1977).

89. J. C. Rage, *Palaeophis colossaeus* nov. sp. (Ie plus grand Seprent connu?) de l'Eocène du Mali et Ie problème du genre chez les Palaeopheinae. *Comptes Rendus de l’Académie des Sciences de Paris* **2**, 1741-1744 (1983).

90. R. S. Rana *et al.*, High diversity of acrodontan lizards in the Early Eocene Vastan Lignite Mine of India. *Geologica Belgica* (2013).

91. O. Rieppel, L. Grande, The anatomy of the fossil varanid lizard *Saniwa ensidens* Leidy, 1870, based on a newly discovred complete skeleton. *Journal of Paleontology* **81**, 643-665 (2007).

92. J. P. Rio, P. D. Mannion, The osteology of the giant snake *Gigantophis garstini* from the upper Eocene of North Africa and its bearing on the phylogenetic relationships and biogeography of Madtsoiidae. *Journal of Vertebrate Paleontology* **37**, e1347179 (2017).

93. A. Sahni, V. C. Srivastava, Eocene rodents and associated reptiles from the Subathu Formation of northwestern India. *Journal of Paleontology*, 922-928 (1976).

94. A. Scanferla, K. T. Smith, S. F. Schaal, Revision of the cranial anatomy and phylogenetic relationships of the Eocene minute boas *Messelophis variatus* and *Messelophis ermannorum* (Serpentes, Booidea). *Zoological Journal of the Linnean Society* **176**, 182-206 (2016).

95. J. Scanlon, Australia’s oldest known snakes: *Patagoniophis*, *Alamitophis*, and cf. *Madtsoia* (Squamata: Madtsoiidae) from the Eocene of Queensland. *Memoirs of the Queensland Museum* **51**, 215-235 (2005).

96. S. Schaal, S. Baszio, *Messelophis ermannorum* n. sp., eine neue Zwergboa (Serpentes: Boidae: Tropidopheinae) aus dem Mittel-Eozän von Messel. *Courier Forschungsinstitut Senckenberg* **252**, 67-77 (2004).

97. R. A. Schatzinger (1975) Later Eocene (Uintan) lizards from the greater San Diego area, California. (San Diego State University, Department of Geological Sciences).

98. R. A. Schatzinger, New species of *Palaeoxantusia* (Reptilia: Sauria) from the Uintan (Eocene) of San Diego Co., California. *Journal of Paleontology*, 460-471 (1980).

99. G. G. Simpson, A new fossil snake from the *Notostylops* beds of Patagonia. *Bulletin of the American Museum of Natural History* **67**, 1-22 (1933).

100. K. T. Smith, A diverse new assemblage of Late Eocene squamates (Reptilia) from the Chadron formation of North Dakota, USA. *Palaeontologia Electronica* **9**, 1-44 (2006).

101. K. T. Smith, A new lizard assemblage from the earliest Eocene (zone Wa0) of the Bighorn Basin, Wyoming, USA: biogeography during the warmest interval of the Cenozoic. *Journal of Systematic Palaeontology* **7**, 299-358 (2009).

102. K. T. Smith, The long‐term history of dispersal among lizards in the early Eocene: new evidence from a microvertebrate assemblage in the Bighorn Basin of Wyoming, USA. *Palaeontology* **54**, 1243-1270 (2011).

103. K. T. Smith, The evolution of mid-latitude faunas during the Eocene: late Eocene lizards of the Medicine Pole Hills reconsidered. *Bulletin of the Peabody Museum of Natural History* **52**, 3-105 (2011).

104. K. T. Smith, New constraints on the evolution of the snake clades Ungaliophiinae, Loxocemidae and Colubridae (Serpentes), with comments on the fossil history of erycine boids in North America. *Zoologischer Anzeiger-A Journal of Comparative Zoology* **252**, 157-182 (2013).

105. K. T. Smith, J. A. Gauthier, Early Eocene lizards of the Wasatch Formation near Bitter Creek, Wyoming: diversity and paleoenvironment during an interval of global warming. *Bulletin of the Peabody Museum of Natural History* **54**, 135-230 (2013).

106. K. T. Smith, J. Habersetzer, The anatomy, phylogenetic relationships, and autecology of the carnivorous lizard “*Saniwa*” *feisti* Stritzke, 1983 from the Eocene of Messel, Germany. *Comptes Rendus Palevol* **20**, 441-506 (2021).

107. K. T. Smith, A. Scanferla, Fossil snake preserving three trophic levels and evidence for an ontogenetic dietary shift. *Palaeobiodiversity and Palaeoenvironments* **96**, 589-599 (2016).

108. T. Smith *et al.*, New early Eocene vertebrate assemblage from western India reveals a mixed fauna of European and Gondwana affinities. *Geoscience Frontiers* **7**, 969-1001 (2016).

109. M. R. Stocker, E. C. Kirk, The first amphisbaenians from Texas, with notes on other squamates from the middle Eocene Purple Bench locality. *Journal of Vertebrate Paleontology* **36**, e1094081 (2016).

110. R. M. Sullivan, Revision of the Paleogene genus *Glyptosaurus* (Reptilia, Anguidae). *Bulletin of the American Museum of Natural History* **163**, 1-72 (1979).

111. R. M. Sullivan, M. Augé, Redescription of the holotype of *Placosaurus rugosus* Gervais 1848–1852 (Squamata, Anguidae, Glyptosaurinae) from the Eocene of France and a revision of the genus. *Journal of Vertebrate Paleontology* **26**, 127-132 (2006).

112. R. M. Sullivan, M. Augé, E. Wille, R. Smith, A new glyptosaurine lizard from the earliest Eocene of Dormaal, Belgium. *Bulletin de la Société géologique de France* **183**, 627-633 (2012).

113. R. M. Sullivan, L. Dong, *Stenoplacosaurus mongoliensis*, a new generic name for *Placosaurus* *mongoliensis* (Anguidae, Glyptosaurinae) from the Shara Murun Formation, Nei Mongol (Inner Mongolia), China. *Fossil Record* **6**, 691-694 (2018).

114. R. M. Sullivan, S. G. Lucas, Fossil Squamata from the San José Formation, early Eocene, San Juan Basin, New Mexico. *Journal of Paleontology*, 631-639 (1988).

115. S. Weber, *Ornatocephalus metzleri* gen. et spec. nov. (Lacertilia, Scincoidea) – Taxonomy and Paleobiology of a basal scincoid lizard from the Messel Formation (Middle Eocene: basal Lutetian, Geiseltalium), Germany. *Abhandlungen der Senckenbergischen Naturforschenden Gesellschaft* **561**, 1-159 (2004).

116. R. Weems, Reptile remains from the Fisher/Sullivan site. *Virginia Division of Mineral Resources Publication* **152**, 101-121 (1999).

117. J. W. Westgate, J. F. Ward, The giant aquatic snake *Pterosphenus* *schucherti* (Palaeophidae) in Arkansas and Mississippi. *Journal of Vertebrate Paleontology* **1**, 161-164 (1981).

118. D. Zhi-Ming, A new species of *Tinosaurus* from Lushih, Honan. *Vertebrata PalAsiatica* **9**, 79-82 (1965).

119. J.-P. Zonneveld, G. F. Gunnell, W. S. Bartels, Early Eocene fossil vertebrates from the southwestern Green River Basin, Lincoln and Uinta Counties, Wyoming. *Journal of Vertebrate Paleontology* **20**, 369-386 (2000).

***Oligocene Squamate References***

1. M. Augé, R. Smith, An assemblage of early Oligocene lizards (Squamata) from the locality of Boutersem (Belgium), with comments on the Eocene-Oligocene transition. *Zoological Journal of the Linnean Society* **155**, 148-170 (2009).

2. M. L. Augé, Une espéce nouvelle ď*Ophisaurus* (Lacertilia, Anguidae) de ľOligocène des phosphorites du Quercy. révision de la sous-famille des Anguinae. *Paläontologische Zeitschrift* **66**, 159-175 (1992).

3. M. L. Augé, Évolution des lézards du Paléogène en Europe. *Mémoires du Muséum national d'histoire naturelle* **192**, 3-369 (2005).

4. G. Baur, The discovery of Miocene amphisbaenians. *American Naturalist* **27**, 998-999 (1893).

5. D. S. Berman, A new amphisbaenian (Reptilia: Amphisbaenia) from the Oligocene-Miocene John Day Formation, Oregon. *Journal of Paleontology*, 165-174 (1976).

6. M. Böhme, Ectothermic vertebrates (Teleostei, Allocaudata, Urodela, Anura, Testudines, Choristodera, Crocodylia, Squamata) from the Upper Oligocene of Oberleichtersbach (Northern Bavaria, Germany). *Courier Forschungsinstitut Senckenberg* **260**, 161-183 (2008).

7. A. Čerňanský, The first potential fossil record of a dibamid reptile (Squamata: Dibamidae): a new taxon from the early Oligocene of Central Mongolia. *Zoological Journal of the Linnean Society* **187**, 782-799 (2019).

8. A. Čerňanský, M. L. Auge, New species of the genus *Plesiolacerta* (Squamata: Lacertidae) from the upper Oligocene (MP28) of Southern Germany and a revision of the type species *Plesiolacerta lydekkeri*. *Palaeontology* **56**, 79-94 (2013).

9. A. Čerňanský, J. Klembara, J. Műller, The new rare record of the late Oligocene lizards and amphisbaenians from Germany and its impact on our knowledge of the European terminal Palaeogene. *Palaeobiodiversity and Palaeoenvironments* **96**, 559-587 (2016).

10. E. D. Cope, *Synopsis of new Vertebrata from the Tertiary of Colorado: obtained during the summer of 1873* (US Government Printing Office, 1873).

11. R. Estes, *Handbuch Der Paläoherpetologie: Sauria Terrestria, Amphisbaenia. Teil 10A. Part 10A* (Lubrecht & Cramer Limited, 1983).

12. H. Filhol, *Recherches sur les phosphorites du Quercy: étude des fossiles qu'on y rencontre et spécialement des mammifères* (G. Masson, 1877), vol. 2.

13. C. W. Gilmore, *Fossil lizards of North America* (U.S. G.P.O., Washington, 1928).

14. C. W. Gilmore, Fossil Snakes of North America. *Geological Society of America, Special Papers* **9**, 1-96 (1938).

15. C. W. Gilmore, Fossil lizards of Mongolia. *Bulletin of the American Museum of Natural History* **81**, 361-385 (1943).

16. J. A. Holman, Snakes of the Gering Formation (Lower Miocene) of Nebraska. *Herpetologica*, 88-94 (1976).

17. J. A. Holman, A herpetofauna from an eastern extension of the Harrison Formation (early Miocene: Arikareean), Cherry County, Nebraska. *Journal of Vertebrate Paleontology* **1**, 49-56 (1981).

18. J. A. Holman, *Geringophis* (Serpentes: Boidae) from the Middle Oligocene of Nebraska. *Herpetologica*, 489-492 (1982).

19. J. A. Holman, A new species of *Helagras* (Serpentes) from the Middle Oligocene of Nebraska. *Journal of Herpetology* **17**, 417-419 (1983).

20. J. A. Holman, *Texasophis galbreathi*, new species, the earliest New World colubrid snake. *Journal of Vertebrate Paleontology* **3**, 223-225 (1984).

21. J. A. Holman, Early Oligocene [Whitneyan] snakes from Florida [USA], the second oldest colubrid snakes in the North America. *Acta zoologica cracoviensia* **42** (1999).

22. J. A. Holman, D. L. Harrison, Early Oligocene [Whitneyan] snakes from Florida [USA]: remaining boids, indeterminate colubroids, summary and discussion of the I-75 Local Fauna snakes. *Acta zoologica cracoviensia* **44** (2001).

23. J. E. Martin, M. N. Hutchinson, R. Meredith, J. A. Case, N. S. Pledge, The oldest genus of scincid lizard (Squamata) from the Tertiary Etadunna Formation of South Australia. *Journal of Herpetology* **38**, 180-187 (2004).

24. J. A. McCartney, N. J. Stevens, P. M. O’Connor, The earliest Colubroid-dominated snake fauna from Africa: perspectives from the Late Oligocene Nsungwe Formation of southwestern Tanzania. *PLoS One* **9**, e90415 (2014).

25. D. C. Parris, J. A. Holman, An Oligocene snake from a coprolite. *Herpetologica*, 258-264 (1978).

26. J.-C. Rage, Les serpents du phosphorites de Quercy. *Palaeovertebrata* **6**, 274-303 (1974).

27. M. S. Stevens (1977) Further study of Castolon Local Fauna (early Miocene) Big Bend National Park, Texas. (Texas Memorial Museum, The University of Texas at Austin).

28. R. M. Sullivan, *Parophisaurus pawneensis* (Gilmore, 1928) new genus of anguid lizard from the Middle Oligocene of North America. *Journal of Herpetology*, 115-133 (1987).

29. Z. Szyndlar, Oligocene snakes of southern Germany. *Journal of Vertebrate Paleontology* **14**, 24-37 (1994).

30. Z. Szyndlar, W. Böhme, Redescriprion of *Tropidonotus atavus* von Meyer, 1855 from the Upper Oligocene of Rott (Germany) and its allocation to *Rottophis* gen. nov.(Serpentes, Boidae). *Palaeontographica Abteilung A*, 145-161 (1996).

31. Z. Szyndlar, J. C. Rage, *Non-erycine Booidea from the Oligocene and Miocene of Europe* (Inst. of systematics a. evolution of animals, Pol. acad. of sciences, 2003).

32. Z. Szyndlar, R. Smith, J.-C. Rage, A new dwarf boa (Serpentes, Booidea,‘Tropidophiidae’) from the Early Oligocene of Belgium: a case of the isolation of Western European snake faunas. *Zoological Journal of the Linnean Society* **152**, 393-406 (2008).

33. E. H. Taylor, *Concerning Oligocene amphisbaenid reptiles* (University of Kansas, 1951).

34. M. Vianey-Liaud *et al.*, A new early Late Oligocene (MP 26) continental vertebrate fauna from Saint-Privat-des-Vieux (Alès Basin, Gard, Southern France). *Geodiversitas* **36**, 565-622 (2014).

***Miocene Squamate References***

1. A. M. Albino, Snakes from the Miocene of Patagonia (Argentina) Part I: the Booidea. *Neues Jahrbuch für Geologie und Paläontologie-Abhandlungen* **199**, 417-434 (1996).

2. W. Auffenberg, The fossil snakes of Florida: Tulane Studies in Zoology, v. 10. (1963).

3. W. Auffenberg, C. C. Mook, C. S. Williams, A new genus of colubrid snake from the Upper Miocene of North America. American Museum novitates; no. 1874. (1958).

4. F. Bachmayer, Z. Szyndlar, Ophidians (Reptilia: Serpentes) from the Kohfidisch fissures of Burgenland, Austria. *Annalen des Naturhistorischen Museums in Wien* **87**, 79-100 (1985).

5. F. Bachmayer, Z. Szyndlar, A second contribution to the ophidian fauna (Reptilia: Serpentes) of Kohfidisch, Austria. *Ann. Naturhist. Mus. Wien* **88**, 25-39 (1987).

6. M. Böhme, Ectothermic vertebrates (Actinopterygii, Allocaudata, Urodela, Anura, Crocodylia, Squamata) from the Miocene of Sandelzhausen (Germany, Bavaria) and their implications for environment reconstruction and palaeoclimate. *Paläontologische Zeitschrift* **84**, 3-41 (2010).

7. W. Böhme, Erstfund eines fossilen Kugelfingergeckos (Sauria: Gekkonidae: Sphaerodactylinae) aus Dominikanischem Bernstein (OligozaÈn von Hispaniola, Antillen). *Salamandra (Frankfurt am Main)* **20**, 212-220 (1984).

8. A. Bolet *et al.*, An amphisbaenian skull from the European Miocene and the evolution of Mediterranean worm lizards. *PLoS One* **9**, e98082 (2014).

9. S. J. Bolkay, *Additions to the fossil herpetology of Hungary from the Pannonian and Praeglacial periode* (Buchdruckerei des Franklin-Verein, 1913).

10. A. Čerňanský, A revision of chamaeleonids from the Lower Miocene of the Czech Republic with description of a new species of Chamaeleo (Squamata, Chamaeleonidae). *Geobios* **43**, 605-613 (2010).

11. A. Čerňanský, J. D. Daza, A. M. Bauer, Geckos from the middle Miocene of Devínska Nová Ves (Slovakia): new material and a review of the previous record. *Swiss Journal of Geosciences* **111**, 183-190 (2018).

12. A. Čerňanský, J. Klembara, K. T. Smith, Fossil lizard from central Europe resolves the origin of large body size and herbivory in giant Canary Island lacertids. *Zoological Journal of the Linnean Society* **176**, 861-877 (2016).

13. A. Čerňanský, J.-C. Rage, J. Klembara, The Early Miocene squamates of Amöneburg (Germany): the first stages of modern squamates in Europe. *Journal of Systematic Palaeontology* **13**, 97-128 (2015).

14. A. Čerňanský, E. Syromyatnikova, E. Kovalenko, K. Podurets, A. Kaloyan, The Key to Understanding the European Miocene Chalcides (Squamata, Scincidae) Comes from Asia: The Lizards of the East Siberian Tagay Locality (Baikal Lake) in Russia. *The Anatomical Record* **303**, 1901-1934 (2020).

15. A. Charig, C. Gans, Two new amphisbaenians from the Lower Miocene of Kenya. *Bulletin of the British Museum, Natural History. Geology* **46**, 19-36 (1990).

16. L. M. Clos, A new species of *Varanus* (Reptilia: Sauria) from the Miocene of Kenya. *Journal of Vertebrate Paleontology* **15**, 254-267 (1995).

17. J. D. Daza, A. M. Bauer, A new amber-embedded sphaerodactyl gecko from Hispaniola, with comments on morphological synapomorphies of the Sphaerodactylidae. *Breviora* **529**, 1-28 (2012).

18. J. D. Daza, A. M. Bauer, E. D. Snively, On the fossil record of the Gekkota. *The Anatomical Record* **297**, 433-462 (2014).

19. E. Douglass, New vertebrates from the Montana Tertiary: Carnegie Museum Annals, v. 2. (1903).

20. R. Estes, A new gerrhonotine lizard from the Pliocene of California. *Copeia*, 676-680 (1963).

21. R. Estes, *Handbuch Der Paläoherpetologie: Sauria Terrestria, Amphisbaenia. Teil 10A. Part 10A* (Lubrecht & Cramer Limited, 1983).

22. G. Georgalis, A. Villa, I. Martin, D. Vasilyan, M. Delfino, Fossil amphibians and reptiles from the Neogene locality of Maramena (Greece), the most diverse European herpetofauna at the Miocene/Pliocene transition boundary. (2019).

23. C. W. Gilmore, Fossil Snakes of North America. *Geological Society of America, Special Papers* **9**, 1-96 (1938).

24. L. Ginsburg, La faune miocène de Sansan et son environnement. *Mémoires du Muséum national d'histoire naturelle (1993)* **183** (2000).

25. R. Hoffstetter, Les serpents du Néogène du Pakistan (couches des Siwaliks). *Bulletin de la Société géologique de France* **7**, 467-474 (1964).

26. R. Hoffstetter, R. JC, Le gisement de vertébrés miocènes de La Venta (Colombie) et sa faune de serpents. (1977).

27. J. A. Holman, Fossil snakes from the Valentine Formation of Nebraska. *Copeia*, 631-637 (1964).

28. J. A. Holman, Herpetofauna of the Wood Mountain Formation (Upper Miocene) of Saskatchewan. *Canadian Journal of Earth Sciences* **7**, 1317-1325 (1970).

29. J. A. Holman, Reptiles of the Egelhoff local fauna (Upper Miocene) of Nebraska. (1973).

30. J. A. Holman, New amphibians and reptiles from the Norden Bridge fauna (upper Miocene) of Nebraska. *Michigan Academician* **6**, 149-163 (1973).

31. J. A. Holman, Herpetofauna of the WaKeeney local fauna (lower Pliocene: Clarendonian) of Trego County, Kansas. *University of Michigan Papers on Paleontology* **12**, 49-66 (1975).

32. J. A. Holman, Snakes of the Split Rock formation (middle Miocene), central Wyoming. *Herpetologica*, 419-426 (1976).

33. J. A. Holman, Snakes from the Rosebud formation (middle Miocene) of South Dakota. *Herpetologica*, 41-48 (1976).

34. J. A. Holman, Additional snakes from the Miocene of western Nebraska. *Herpetologica*, 443-446 (1977).

35. J. A. Holman, Amphibians and reptiles from the Gulf Coast Miocene of Texas. *Herpetologica*, 391-403 (1977).

36. J. A. Holman, New herpetological species and records from the Norden Bridge fauna (Miocene: Late Barstovian) of Nebraska. (1982).

37. J. A. Holman, Texasophis (Reptilia: Serpentes), an Addition to the Miocene (Clarendonian) of North America. *Copeia*, 660-661 (1984).

38. J. A. Holman, Reptiles of the lower Miocene (Hemingfordian) Pollack Farm Fossil Site, Delaware. *Geology and Paleontology of the Lower Miocene Pollack Farm Fossil Site, Delaware. Delaware Geological Survey Special Publication* **21**, 141-147 (1998).

39. A. S. Hsiou, A. M. Albino, J. Ferigolo, Reappraisal of the South American Miocene snakes of the genus *Colombophis*, with description of a new species. *Acta Palaeontologica Polonica* **55**, 365-379 (2010).

40. M. Hutchinson, The first fossil pygopod (Squamata, Gekkota), and a review of mandibular variation in living species. *Memoirs-Queensland Museum* **41**, 355-366 (1997).

41. M. Ivanov, The oldest known Miocene snake fauna from Central Europe: Merkur-North locality, Czech Republic. *Acta Palaeontologica Polonica* **47** (2002).

42. M. Ivanov, M. Ruta, J. Klembara, M. Böhme, A new species of *Varanus* (Anguimorpha: Varanidae) from the early Miocene of the Czech Republic, and its relationships and palaeoecology. *Journal of Systematic Palaeontology* **16**, 767-797 (2018).

43. J. Klembara, A new anguimorph lizard from the Lower Miocene of North‐West Bohemia, Czech Republic. *Palaeontology* **51**, 81-94 (2008).

44. J. Klembara, A new species of *Pseudopus* (Squamata, Anguidae) from the early Miocene of Northwest Bohemia (Czech Republic). *Journal of Vertebrate Paleontology* **32**, 854-866 (2012).

45. J. Klembara, New finds of anguines (Squamata, Anguidae) from the Early Miocene of Northwest Bohemia (Czech Republic). *Paläontologische Zeitschrift* **89**, 171-195 (2015).

46. J. Klembara, M. BÖHME, M. Rummel, Revision of the anguine lizard *Pseudopus laurillardi* (Squamata, Anguidae) from the Miocene of Europe, with comments on paleoecology. *Journal of Paleontology* **84**, 159-196 (2010).

47. J. Klembara, M. Rummel, New material of *Ophisaurus*, *Anguis* and *Pseudopus* (Squamata, Anguidae, Anguinae) from the Miocene of the Czech Republic and Germany and systematic revision and palaeobiogeography of the Cenozoic Anguinae. *Geological Magazine* **155**, 20-44 (2018).

48. J. D. Lazell Jr, An *Anolis* (Sauria, Iguanidae) in amber. *Journal of Paleontology*, 379-382 (1965).

49. J. Müller, Eine neue art der echten eidechsen (Reptilia: Lacertilia: Lacertidae) aus dem Unteren Miozän von Poncenat, Frankreich. *Mainzer Geowissenschaftliche Mitteilungen* **25**, 79-88 (1996).

50. J. Müller, A new fossil species of *Euleptes* from the early Miocene of Montaigu, France (Reptilia, Gekkonidae). *Amphibia-reptilia* **22**, 341-348 (2001).

51. M. A. Norell, K. De Queiroz, The earliest iguanine lizard (Reptilia: Squamata) and its bearing on iguanine phylogeny. *American Museum Novitates* (1991).

52. D. Parmley, Early Hemphillian (late Miocene) snakes from the Higgins local fauna of Lipscomb County, Texas. *Journal of Vertebrate Paleontology* **8**, 322-327 (1988).

53. D. Parmley, J. A. Holman, Hemphillian (late Miocene) snakes from Nebraska, with comments on Arikareean through Blancan snakes of midcontinental North America. *Journal of Vertebrate Paleontology* **15**, 79-95 (1995).

54. D. Parmley, K. B. Hunter, Fossil snakes of the Clarendonian (late Miocene) Pratt Slide local fauna of Nebraska, with the description of a new natricine colubrid. *Journal of Herpetology* **44**, 526-543 (2010).

55. J. A. Peters, A fossil snake of the genus *Heterodon* from the Pliocene of Kansas. *Journal of Paleontology*, 328-331 (1953).

56. A. B. Quadros, P. Chafrat, H. Zaher, A new teiid lizard of the genus *Callopistes* Gravenhorst, 1838 (Squamata, Teiidae), from the Lower Miocene of Argentina. *Journal of Vertebrate Paleontology* **38**, (1)-(18) (2018).

57. J.-C. Rage, Les Squamates du Miocène de Beni Mellal, Maroc. *Géologie méditerranéenne* **3**, 57-69 (1976).

58. J.-C. Rage, S. S. Gupta, G. V. Prasad, Amphibians and squamates from the Neogene Siwalik beds of Jammu and Kashmir, India. *PalZ* **75**, 197-205 (2001).

59. J.-C. Rage, J. A. Holman, Des Serpents (Reptilia, Squamata) de type Nord-Américain dans le Miocène français. Évolution parallèle ou dispersion? *Geobios* **17**, 89-104 (1984).

60. J.-C. Rage, Z. Szyndlar, West Palearctic cobras of the genus *Naja* (Serpentes: Elapidae): interrelationships among extinct and extant species. *Amphibia-Reptilia* **11**, 385-400 (1990).

61. Z. Roček, Lizards (Reptilia: Sauria) from the Lower Miocene locality Dolnice (Bohemia, Czechoslovakia). *Rozpravy Československé Akademie věd. Řada matematických a přírodních věd* **93** (1984).

62. J. D. Scanlon, A new large madtsoiid snake from the Miocene of the Northern Territory. *Beagle: Records of the Museums and Art Galleries of the Northern Territory, The* **9**, 49-59 (1992).

63. J. D. Scanlon, *Nanowana* gen. nov., small madtsoiid snakes from the Miocene of Riversleigh: sympatric species with divergently specialised dentition. *Memoirs of the Queensland Museum* **41**, 393-412 (1997).

64. J. D. Scanlon, M. S. Lee, The Pleistocene serpent *Wonambi* and the early evolution of snakes. *Nature* **403**, 416-420 (2000).

65. J. D. Scanlon, M. S. Lee, M. Archer, Mid-Tertiary elapid snakes (Squamata, Colubroidea) from Riversleigh, northern Australia: early steps in a continent-wide adaptive radiation. *Geobios* **36**, 573-601 (2003).

66. H.-H. Schleich, Die mittelmiozäne Fossil-Lagerstätte Sandelzhausen. 13. *Chamaeleo bavaricus* sp. nov., ein neuer Nachweis aus dem Jungtertiär Süddeutschlands. *Mitteilungen der Bayerischen Staatssammlung für Paläontologie und Historische Geologie*, 77-81 (1983).

67. H. H. Schleich, Neue Reptilienfunde aus dem Tertiär Deutschlands 8. *Palaeoblanus tobieni* n. gen., n. sp. neue Doppelschleichen aus dem Tertiär Deutschlands. *Paläontologische Zeitschrift* **62**, 95-105 (1988).

68. M. Smith, M. Plane, Pythonine snakes (Boidae) from the Miocene of Australia. *BMR Journal of Australian Geology & Geophysics* **9**, 191-195 (1984).

69. Z. Szyndlar, Ophidian fauna (Reptilia, Serpentes) from the uppermost Miocene of Algora (Spain). *Estudios Geológicos (Madrid)* **41**, 447-465 (1985).

70. Z. Szyndlar, Snakes from the lower Miocene locality of Dolnice (Czechoslovakia). *Journal of Vertebrate Paleontology* **7**, 55-71 (1987).

71. Z. Szyndlar, J. C. Rage, *Non-erycine Booidea from the Oligocene and Miocene of Europe* (Inst. of systematics a. evolution of animals, Pol. acad. of sciences, 2003).

72. Z. Szyndlar, H. H. Schleich, Description of Miocene Snakes from Petersbuch 2 with Comments on the Lower and Middle Miocene Ophidian Faunas of Southern Germany: Stuttgarter Beiträge zur Naturkunde: Reihe B/Geologie und: Paläontologie. (1993).

73. Z. Szyndlar, G. Zerova, Miocene snake fauna from Cherevichnoie (Ukraine, USSR), with description of a new species of Vipera. *Neues Jahrbuch für Geologie und Paläontologie. Abhandlungen* **184**, 87-99 (1992).

74. Z. Szyndlar, G. A. Zerova, Neogene Cobras of the Genus *Naja* (Serpentes: Elapidae) of East Europe/Neogene Kobras der Gattung *Naja* (Serpentes: Elapidae) aus Osteuropa. *Annalen des Naturhistorischen Museums in Wien. Serie A für Mineralogie und Petrographie, Geologie und Paläontologie, Anthropologie und Prähistorie*, 53-61 (1987).

75. K. M. Thorn, M. N. Hutchinson, M. Archer, M. S. Lee, A new scincid lizard from the Miocene of northern Australia, and the evolutionary history of social skinks (Scincidae: Egerniinae). *Journal of Vertebrate Paleontology* **39**, e1577873 (2019).

76. J. W. Twente, Pliocene lizards from Kansas. *Copeia* **1952**, 70-73 (1952).

77. P. Vanzolini, Fossil snakes and lizards from the lower Miocene of Florida. *Journal of Paleontology*, 452-457 (1952).

78. M. Venczel, Late Miocene snakes from Polgárdi (Hungary). *Acta zoologica cracoviensia* **37**, 1-29 (1994).

79. M. Venczel, Late Miocene snakes [Reptilia: Serpentes] from Polgardi [Hungary]: a second contribution. *Acta zoologica cracoviensia* **41** (1998).

80. M. Venczel, E. Stiuca, Late middle Miocene amphibians and squamate reptiles from Taut, Romania. *Geodiversitas* **30**, 731-763 (2008).

81. A. Villa *et al.*, Revision of *Varanus marathonensis* (Squamata, Varanidae) based on historical and new material: morphology, systematics, and paleobiogeography of the European monitor lizards. *PloS one* **13**, e0207719 (2018).

82. D. A. Yatkola, Mid-Miocene lizards from western Nebraska. *Copeia* **1976**, 645-654 (1976).

83. G. Zerova, *Vipera (Daboia) ukrainica* - a new viper (Serpentes; Viperidae) from the Middle Sarmatian (Upper Miocene) of the Ukraine. *Neues Jahrbuch für Geologie und Paläontologie. Abhandlungen* **184**, 235-249 (1992).

***Plio-Pleistocene Squamate References***

1. S. Bailon, An Aniliid (Reptilia, Serpentes) in the Late European Pliocene. *Comptes Rendus de l’Académie des Sciences-Series II* **306**, 1255-1258 (1988).

2. S. Bailon, Amphibiens et reptiles du Pliocène terminal d’Ahl al Oughlam (Casablanca, Maroc). *Geodiversitas* **22**, 539-558 (2000).

3. S. Bailon, M. Auge, Un nouveau genre, *Ragesaurus* (Squamata, Anguidae, Anguinae), du Pléistocène inférieur des îles Medas (Catalogne, Espagne). *Bulletin de la Société géologique de France* **183**, 683-688 (2012).

4. S. Bailon, J. Garcia-Porta, J. Quintana-Cardona, Première découverte de Viperidae (Reptilia, Serpentes) dans les îles Baléares (Espagne): des vipères du Néogène de Minorque. Description d’une nouvelle espèce du Pliocène. *Comptes Rendus Palevol* **1**, 227-234 (2002).

5. C. Bochaton, S. Bailon, A new fossil species of *Boa* Linnaeus, 1758 (Squamata, Boidae), from the Pleistocene of Marie-Galante Island (French West Indies). *Journal of Vertebrate Paleontology* **38**, e1462829 (2018).

6. B. H. Brattstrom, Records of Pleistocene reptiles from California. *Copeia* **1953**, 174-179 (1953).

7. B. H. Brattstrom, Pliocene and Pleistocene amphibians and reptiles from southeastern Arizona. *Journal of Paleontology*, 150-154 (1955).

8. R. Estes, *Handbuch Der Paläoherpetologie: Sauria Terrestria, Amphisbaenia. Teil 10A. Part 10A* (Lubrecht & Cramer Limited, 1983).

9. C. Gans, R. Montero, Two new fossil amphisbaenids (Reptilia: Squamata) from the Pleistocene of Lagoa Santa (Minas Gerais, Brasil). *Steenstrupia* **24**, 9-22 (1998).

10. C. W. Gilmore, Fossil Snakes of North America. *Geological Society of America, Special Papers* **9**, 1-96 (1938).

11. J. J. Head, P. M. Barrett, E. J. Rayfield, Neurocranial osteology and systematic relationships of *Varanus* (*Megalania*) *prisca* Owen, 1859 (Squamata: Varanidae). *Zoological Journal of the Linnean Society* **155**, 445-457 (2009).

12. J. Holman, Herpetofauna of Ladds Quarry. *National Geographic Research* **1**, 423-436 (1985).

13. M. N. Hutchinson, J. D. Scanlon, New and Unusual Plio-Pleistocene Lizard (Reptilia: Scincidae) from Wellington Caves, New South Wales, Australia. *Journal of Herpetology* **43**, 139-147 (2009).

14. P. A. Meylan, *The squamate reptiles of the Inglis IA fauna (Irvingtonian: Citrus County, Florida)* (University of Florida, 1982).

15. M. Młynarski, Die jungpliozäne Reptilienfauna von Rebielice Królewskie, Polen. *Senckenbergiana biologica* **45**, 325-347 (1964).

16. M. Norrel, Late Cenozoic lizards of the Anza Borrego Desert, California. Contributions in Science. *Natural History Museum of Los Angeles County* **414**, 1-31 (1989).

17. R. Owen, II. Description of some remains of a gigantic land-lizard (*Megalania* *prisca*, Ow.) from Australia. *Proceedings of the Royal Society of London*, 273-273 (1859).

18. J. D. Scanlon, B. S. Mackness, A new giant python from the Pliocene Bluff Downs Local Fauna of northeastern Queensland. *Alcheringa* **25**, 425-437 (2001).

19. M. J. Smith, Small fossil vertebrates from Victoria Cave, Naracoorte, South Australia. IV. Reptiles. (1976).

20. Z. Szyndlar, Fossil snakes from Poland. *Acta zoologica cracoviensia* **28** (1984).
